# A systematic review of genome-wide association studies for pain, nociception, neuropathy, and pain treatment responses

**DOI:** 10.1097/j.pain.0000000000002910

**Published:** 2023-05-05

**Authors:** Song Li, Annika Brimmers, Regina L.M. van Boekel, Kris C.P. Vissers, Marieke J.H. Coenen

**Affiliations:** a Department of Human Genetics, Radboud Institute for Health Sciences, Radboud University Medical Center, Nijmegen, the Netherlands. Coenen is now with the Department of Clinical Chemistry, Erasmus Medical Center, Rotterdam, the Netherlands; b Department of Anesthesiology, Pain and Palliative Medicine, Radboud University Medical Center, Nijmegen, the Netherlands

**Keywords:** Pain, Nociception, Neuralgia, Genome-wide association studies, Systematic review

## Abstract

Pain is the leading cause of disability worldwide, imposing an enormous burden on personal health and society. Pain is a multifactorial and multidimensional problem. Currently, there is (some) evidence that genetic factors could partially explain individual susceptibility to pain and interpersonal differences in pain treatment response. To better understand the underlying genetic mechanisms of pain, we systematically reviewed and summarized genome-wide association studies (GWASes) investigating the associations between genetic variants and pain/pain-related phenotypes in humans. We reviewed 57 full-text articles and identified 30 loci reported in more than 1 study. To check whether genes described in this review are associated with (other) pain phenotypes, we searched 2 pain genetic databases, Human Pain Genetics Database and Mouse Pain Genetics Database. Six GWAS-identified genes/loci were also reported in those databases, mainly involved in neurological functions and inflammation. These findings demonstrate an important contribution of genetic factors to the risk of pain and pain-related phenotypes. However, replication studies with consistent phenotype definitions and sufficient statistical power are required to validate these pain-associated genes further. Our review also highlights the need for bioinformatic tools to elucidate the function of identified genes/loci. We believe that a better understanding of the genetic background of pain will shed light on the underlying biological mechanisms of pain and benefit patients by improving the clinical management of pain.

## Abstract 2

Supplemental Digital Content is Available in the Text.

## 1. Introduction

Pain, especially chronic pain, is a condition that greatly impacts the quality of life. The prevalence of chronic pain in adults is approximately 20%,^4,147^ and it increases with age.^31^ Chronic painful conditions are the leading causes of years lived with disability,^40^ and they can contribute to the development of other health conditions, such as disability, depression, and sleep disturbances.^4^ Besides the burden for patients, chronic pain can have enormous socioeconomic consequences directly or indirectly, eg, because of absenteeism. For example, the total costs associated with only low back pain in European countries are estimated to be 0.1% to 2% of gross domestic product.^100,141^

Pain is a subjective experience with very heterogeneous presentations. Pain can be acute or chronic; acute pain is usually associated with tissue damage and generally eases with the healing of tissue,^131^ whereas chronic pain persists or recurs for more than 3 months.^131^ Chronic pain can develop without a clear etiology or pathophysiology (chronic primary pain, eg, fibromyalgia) or secondary to an underlying disease (chronic secondary pain, eg, chronic pain associated with osteoarthritis). There is a distinction between nociceptive pain (pain from ongoing tissue inflammation or damage) and neuropathic pain (pain caused by nerve damage). More recently, the term nociplastic pain has been proposed to describe the clinically and psychophysically altered nociception that cannot directly be linked to nociceptive or neuropathic pain.^33^ Nociplastic pain is defined as *pain that arises from altered nociception despite no clear evidence of actual or threatened tissue damage causing the activation of peripheral nociceptors or evidence for disease or lesion of the somatosensory system causing the pain* (International Association for the Study of Pain [IASP] terminology). Examples of nociplastic pain are fibromyalgia and irritable bowel syndrome. Although there are differences in the pathways leading to the different types of pain, part of the underlying mechanisms may be shared, such as structural changes in the brain,^79^ central sensitization,^95^ and neurochemical imbalances in the central nervous system.^20^

The risk of developing pain can be attributed to sociodemographic factors (eg, age, female gender, and occupation),^42,45^ psychological factors (eg, depression),^80^ clinical factors (eg, chronic disease),^88^ and lifestyle factors.^134^ In addition, preexisting pain is related to the development of other types of pain. For instance, acute postoperative pain is a risk factor for chronic pain development after surgery.^11^ Besides these factors, genetic susceptibility could also contribute to the development of pain. Indeed, heritability estimates for different pain phenotypes range from 30% to 70%, indicating that genetics contributes.^19^ For instance, the heritability of neuropathic pain, low back pain, and neck pain are estimated to be approximately 37%, 52% to 68%, and 35% to 58%, respectively.^75,90^

Although numerous genetic risk factors have been described for pain development and unsatisfied pain treatment response, the underlying genetic mechanisms remain elusive. One reason might be that most published studies use a hypothesis-driven approach, thus focusing on specific genes/pathways with known functions, which might be biased by previous knowledge of the etiology of pain.^106^ The 2 most investigated genes related to pain are *COMT* (involved in neurotransmission) and *OPRM1* (encoding opioid receptor).^3,47^ However, no consistent associations with pain have been observed for both genes from candidate gene studies.^93,106^ Hypothesis-free methods like genome-wide association studies (GWASes) are more appropriate for finding additional genes beyond known mechanisms. Indeed, GWASes have identified many putative causal genes other than the previously described candidate genes, which shed new light on the mechanism of pain development.^34^ Unfortunately, most candidate and genome-wide association studies on pain report inconsistent results, which is partly due to the low statistical power of the studies. Therefore, few findings are convincing enough to be investigated further.

Besides contributing to pain development, current evidence suggests that genetic variabilities can also contribute to pain treatment response differences in efficacy and side effects.^30,119^ To date, several studies investigated the association between genetic variants and treatment efficacy and adverse event in the 2 most common drug categories for pain management, namely, nonsteroidal anti-inflammatory drugs (NSAIDs) and opioid analgesics.^10^ A clear example is codeine treatment outcome and genetic variants in the drug-metabolizing gene *CYP2D6*.^10,22^

In this systematic review, we aimed to summarize GWASes investigating pain, nociception, neuropathy, and pain treatment responses in humans to provide an overview of the potential genetic risk factors for pain. In addition, the overlap of the identified genes in all included studies is summarized, aiming to fill the knowledge gap on the shared genetic background of pain syndromes. To provide additional evidence for the role of the identified genes in pain, we examined whether the genes identified in this systematic review were linked to pain by other studies using the human and mouse pain genetics database.

## 2. Method

This systematic review was conducted and reported following Preferred Reporting Items for Systematic Reviews and Meta Analyses guidelines (PRISMA).^103^ This study should be viewed as a descriptive review, and we did not conduct a meta-analysis considering the broad pain phenotypes included in this study.

### 2.1. Systematic search

**Figure 1.**
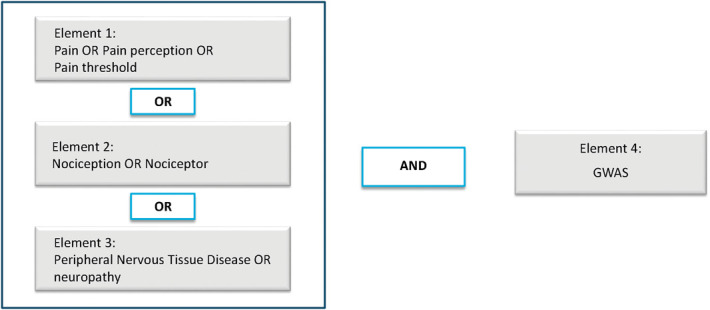
Search strategy for systematic review. GWAS, genome-wide association study.

A systematic literature search was performed to assess all available literature on GWAS of pain, nociception, neuropathy, and pain treatment response. Headaches and migraine were excluded because the underlying biological mechanisms differ from other pain phenotypes. A search term including 4 elements was built. The first 3 terms were included to capture pain, and the fourth term identifies GWASes. The following terms were used: (1) pain, pain perception, or pain threshold, describing “*an unpleasant sensory and emotional experience associated with, or resembling that associated with, actual or potential tissue damage” (IASP terminology)*; (2) nociception or nociceptor, describing “*the neural process of encoding noxious stimuli, which pain sensation is not necessarily implied*” (IASP terminology); nociception (pain sensitivity) was included because it has predictive value in postoperative pain^74^ and pain treatment outcome^29^; (3) neuralgia or peripheral nervous system diseases or neuropathy, describing “*a disturbance of function or pathological change in a nerve: in one nerve, mononeuropathy; in several nerves, mononeuropathy multiplex; if diffuse and bilateral, polyneuropathy*” (IASP terminology); neuropathy is included because it is one of the underlying cause of neuropathic pain; (4) genome-wide association study, a hypothesis-free method to scan associations between genetic variants and phenotypes. A hypothesis-free method is mainly data driven or discovery driven without preset hypotheses, for instance, testing all genetic markers on a genotyping array or whole exome/genome sequencing. The first 3 elements were connected by “OR” and then connected to the last element by “AND” as shown in Figure 1. Science libraries MEDLINE and Embase were searched for relevant literature using MeSH and Emtree terms, respectively (See Table S1, available as supplemental digital content at http://links.lww.com/PAIN/B808). The literature search was performed on February 21, 2022. To validate our search terms, we examined all publications of pain, nociception, and peripheral neuropathy traits in the GWAS Catalog to determine whether we missed relevant publications.

After removing duplicates, all identified articles were assessed by 2 independent reviewers (A.B. and S.L.), and article selection was conducted independently in Rayyan.^102^ A third independent reviewer evaluated articles with conflict decisions to reach a consensus (M.J.H.C. and R.L.M.v.B). As we focused on the pain itself other than the background diseases triggering pain in this review, we excluded articles investigating the diverse background of diseases causing pain. Article selection criteria details can be found in Table S2, available as supplemental digital content at http://links.lww.com/PAIN/B808.

The review was not preregistered as the first intention was to write a narrative review. However, after we performed the search, the number of articles was manageable to write a systematic review. Based on this, we decided to switch to a systematic review.

### 2.2. Quality assessment

The quality of studies was assessed by checking compliance with the “STrengthening the Reporting of Genetic Association studies” (STREGA) guidelines that include 30 key items in 6 categories: title and abstract, introduction, method, results, discussion, and funding information (Table S3, available as supplemental digital content at http://links.lww.com/PAIN/B808).^72^ The quality score was calculated for each study based on the sum of each assessed item. Higher scores represent studies of higher quality. No quality score threshold was set to select articles.

### 2.3. Data extraction

For each article included in this review, the following information was extracted: PubMed identifier (PMID), first author, publication year, outcome phenotype, phenotype variable type (eg, continuous, discrete, time-to-event, or binary), study characteristics (sample size, ethnicity, *P* value threshold applied in the original article, number of significant loci) of discovery, replication, and meta-analysis phase, and single nucleotide polymorphisms (SNPs) associated with the investigated phenotype. The phenotypes investigated in the included articles as defined by the authors of the original publication can be found in Table S4, available as supplemental digital content at http://links.lww.com/PAIN/B808.

To reduce reporting of possible false-positive findings, an upper boundary of P < 1E-5 was set for associated SNPs. We referred to this threshold as a suggestively significant threshold. Throughout the article, we also refer to genome-wide significance defined as the conventional threshold of 5E-8. If multiple SNPs within the same loci/gene were identified, only the most statistically significantly associated SNP was extracted for inclusion in this article. The following information was extracted for selected SNPs: rsid; allele frequency, effect size, and standard error of effect allele and the *P* value in the discovery, replication, and meta-analysis phase if applicable. For effect size, values from the meta-analysis phase were prioritized to report whenever available. If an odds ratio was reported, it was converted to effect size by natural logarithm to make results comparable.

When the included articles indicated that they aimed to replicate GWAS-identified loci from previous studies we included this in the review. We describe this in each phenotype section using the following wordings “replication” or “replicated.” We use the word “overlap” to indicate our own search for overlap between the studies as described in the paragraph below and in section 3.11.

### 2.4. Follow-up research

As genomic position and (nearby) genes of extracted loci were not always reported, and different articles use different reference genome versions for annotation, all extracted variants were reannotated to genes by wANNOWAR^15^ and Ensembl Variant Effect Predictor (VEP).^81^ If a variant was located in an intergenic region, it was mapped to the closest genes (upstream and downstream). Chromosome band was obtained in the University of California, Santa Cruz (UCSC) genome browser.^59^ All annotations were based on *Homo sapiens* (human) genome assembly GRCh37 (hg19).

To investigate whether the identified loci/genes from included articles overlap with the same pain phenotypes or between different pain phenotypes, we first examined the linkage disequilibrium (LD) of extracted variants with LD matrix^76^ in Northern Europeans from Utah (CEU) ancestry. Besides checking LD, all SNPs were mapped to (closest) genes (see above), and the mapped gene symbols were checked for overlap.

All mapped genes from the included GWASes were queried in the human^82^ and mouse^63^ pain genetics database to find additional evidence that the genes contribute to pain phenotypes. The Human Pain Genetic Database (HPGDB) is a comprehensive variant-focused inventory of genetic contributors to human pain summarized from both candidate gene studies and GWASes. This database was updated until July 2021. Before querying, articles already included in this review were removed from the HPGDB. The mouse pain genetic database included 434 genes involved in acute or tonic nociception, injury- or stimulus-induced hypersensitivity (ie, allodynia or hyperalgesia), or drug- or stress-induced inhibition of nociception (ie, analgesia) in the mouse. This database only contains the results of articles published before 2015.

## 3. Results

### 3.1. Literature search

**Figure 2.**
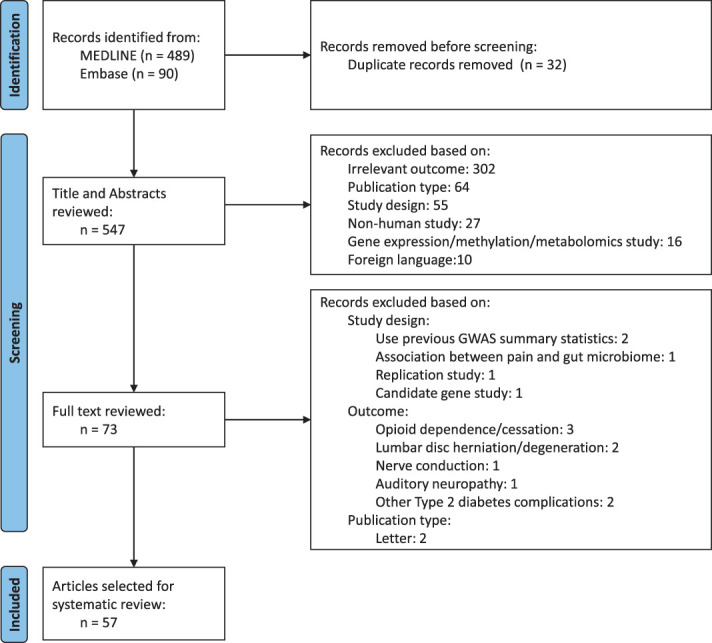
Systematic literature search and assessment process according to PRISMA principles. PRISMA, Preferred Reporting Items for Systematic Reviews and Meta-Analyses.

A literature search in MEDLINE and Embase resulted in the identification of 579 articles. Figure 2 illustrates the article selection workflow and reasons for exclusion. During the screening process, 32 duplicates were removed, and after screening titles and abstracts, 474 articles were excluded because they were not in line with our research question. The full text of the 73 remaining studies was reviewed, which led to the exclusion of 16 articles because of the outcome (n = 9), study design (n = 5), or publication type (n = 2). Details concerning the reason for exclusion are described in Table S5, available as supplemental digital content at http://links.lww.com/PAIN/B808. To ensure that no articles were missed, the GWAS catalog was checked using the phenotype keywords “pain” and “neuropathy.” Five additional articles were identified but not included in this review because they did not meet the inclusion criteria (Table S5, available as supplemental digital content at http://links.lww.com/PAIN/B808).

### 3.2. Included studies

The characteristics of the 57 included articles are summarized in Table 1. The STREGA quality score of the studies ranged from 16 to 29 (see Table S6, available as supplemental digital content at http://links.lww.com/PAIN/B808). Most studies reported on participants with European ancestry (including Hispanic) (n = 44). Thirty-two studies had a relatively small sample size (<1000 samples), whereas the studies with a large sample size mainly included data from the UK Biobank (UKB) (n = 12). Twenty-four studies (42%) did not include a replication cohort (see Table S7, available as supplemental digital content at http://links.lww.com/PAIN/B808 for replication and meta-analysis information of included articles).

**Table 1:** Characteristics of included studies in this review.

PMID	Author, y	Outcome phenotype(s)	Phenotype category	Phenotype variable type	Discovery study design			
					Sample size	Ethnicity	*P* threshold	No. of significant loci
33802509	Adjei, 2021	Paclitaxel; paclitaxel and carboplatin; or oxaliplatin receipt induced sensory symptoms	Cancer pain	Continuous	692	EA; AA; Asian; American Indian or Alaska Native; others	1.00E-06	3
22843789	Baldwin, 2012	Paclitaxel-induced peripheral neuropathy: the maximum grade neuropathy observed Paclitaxel-induced peripheral neuropathy: the cumulative dose level	Cancer pain Cancer pain	Discrete Time to event	855 855	EA EA	1.00E-05 1.00E-05	4 7
28317148	Campo, 2017	Bortezomib‐induced peripheral neuropathy	Cancer pain	Binary	Cases 102 and controls 544	German	1.00E-05	4
32562552	Chua, 2020	Microtubule targeting agents induced peripheral neuropathy	Cancer pain	Time to event	469	EA	1.00E-05	0
24909733	Cook-Sather, 2014	Acute postoperative pain Opioid analgesia	Postoperative pain Pain treatment response	Binary Continuous	In EA, cases 132 and controls 136 In AA, cases 103 and controls 118 EA 277, AA 241	EA; AA EA; AA	1.00E-05 1.00E-05	2 in EA 3 in EA; 9 in AA
25710658	Diouf, 2015	Vincristine-induced peripheral neuropathy	Cancer pain	Binary	Cases 89 and controls 232	EA; AA; Asian; Hispanic; others	1.00E-05	5
24582949	Docampo, 2014	Fibromyalgia	Musculoskeletal pain	Binary	Cases 300 and controls 203	White Spanish	1.00E-05	9
28611204	Dolan, 2017	Cisplatin-induced peripheral neuropathy	Cancer pain	Ordinal	680	EA; others	1.00E-05	13
32681239	Dunbar, 2020	Constant-severe pain in chronic pancreatitis	Visceral pain	Binary	Cases 787 and controls 570	EA	1.00E-05	1
34924555	Fontanillas, 2021	Cold pressor test Pain sensitivity questionnaire score	Pain sensitivity Pain sensitivity	Time to event Continuous	6853 25,321	EA EA	1.00E-06 1.00E-06	1 3
30747904	Freidin, 2019	Chronic back pain	Musculoskeletal pain	Binary	Cases 91,100 and controls 258,900	EA	5.00E-08	5
33021770	Freidin, 2021	Chronic back pain	Musculoskeletal pain	Binary	In males, cases 35,705 and controls 166,372 In females, cases 43,230 and controls 194,524	EA	5.00E-08	7 in females; 2 in males
21622719	Galvan, 2011	Opioid analgesia	Pain treatment response	Discrete	438	EA	1.00E-05	8
27605156	García-Sanz, 2017	Bortezomib and thalidomide induced peripheral neuropathy	Cancer pain	Binary	Cases 40 and controls 132	Not report	1.00E-05	3
27143689	Hertz, 2016	Docetaxel-induced peripheral sensory neuropathy	Cancer pain	Time to event	623	EA	1.05E-05	3
29855537	Hirata, 2018	Dysmenorrhoea pain	Visceral pain	Discrete	11348	Japanese	5.50E-09	2
28025368	Janicki, 2016	Complex regional pain syndrome	Musculoskeletal pain	Binary	Cases 230 and controls 230	EA; AA; Hispanics	2.50E-07	0
31194737	Johnston, 2019	Multisite chronic pain	Musculoskeletal pain	Discrete	387,649	EA	5.00E-08	39
33830993	Johnston, 2021	Multisite chronic pain	Musculoskeletal pain	Discrete	178,556 males and 209,093 females	EA	5.00E-08	10 in females; 5 in males
27454463	Jones, 2016	Dysmenorrhoea pain	Visceral pain	Discrete	11,891	EA	1.00E-06	6
34391895	Kanai, 2021	Oxaliplatin induced peripheral sensory neuropathy: grade 2/3 vs grade 0 Oxaliplatin induced peripheral sensory neuropathy: grade2/3 vs grade 0/1	Cancer pain Cancer pain	Binary Binary	Cases 233 and controls 49 Cases 383 and controls 605	Japanese Japanese	1.00E-05 1.00E-05	2 7
19207018	Kim, 2009	Acute postoperative pain NSAID analgesia	Postoperative pain Pain treatment response	Discrete Continuous	112 112	EA EA	3.30E-08 3.30E-08	0 1
26015512	Komatsu, 2015	Paclitaxel-induced sensory peripheral neuropathy	Cancer pain	Binary	Cases 24 and controls 121	Asian	1.00E-05	4
23776197	Leandro-García, 2013	Paclitaxel-induced peripheral sensory neuropathy	Cancer pain	Time-to event	144	EA	1.05E-05	10
31196165	Lee, 2019	Acute postradiation therapy pain	Postoperative pain	Binary	Cases 326 and controls 786	African American; Hispanic Whites; non-Hispanic Whites; others	1.00E-05	3
24554482	Leger, 2014	Stavudine and didanosine induced peripheral neuropathy	Neuropathic pain	Binary	Cases 90 and controls 164	EA; AA; Hispanic	1.05E-05	5
27764105	Lemmela, 2016	Sciatica	Neuropathic pain	Binary	Cases 291 and controls 3671	Finnish	5.00E-08	2
28447608	Li, 2017	Dysmenorrhoea pain	Visceral pain	Binary	Cases 2404 and controls 2920	Chinese	5.00E-08	0
30506673	Li, 2019	Vincristine-induced peripheral neuropathy	Cancer pain	Time-to event in discovery cohort; discrete in replication cohort	1068	EA	1.05E-05	2
27060151	Magrangeas, 2016	Bortezomib-induced peripheral neuropathy	Cancer pain	Binary	Cases 155 and controls 314	EA; others	1.05E-05	4
24974787	Meng, 2015 #1	Diabetic neuropathic pain	Neuropathic pain	Binary	Cases 572 and controls 2491	EA	1.00E-06	1
26629533	Meng, 2015 #2	Diabetic neuropathic pain	Neuropathic pain	Binary	In males, cases 470 and controls 2021 In females, cases 491 and controls 1239	EA	1.00E-06	1 in overall; 1 in females; 1 in males
31482140	Meng, 2019	Chronic knee pain	Musculoskeletal pain	Binary	Cases 22,204 and controls 149,312	EA	5.00E-08	2
32246137	Meng, 2020	Shoulder and neck pain	Musculoskeletal pain	Binary	Cases 53,994 and controls 149,312	EA	5.00E-08	3
26566055	Mieda, 2016	Opioid analgesia	Pain treatment response	Continuous	350	Japanese	Fisrt and second stage *P* < 0.05; final stage Q < 0.05	1
23183491	Nishizawa, 2014	Opioid analgesia	Pain treatment response	Continuous	355	Japanese	Fisrt and second stage *P* < 0.05; final stage Q < 0.05	1
29207912	Nishizawa, 2018	Opioid analgesia	Pain treatment response	Continuous	350	Japanese	Fisrt and second stage *P* < 0.05; final stage Q < 0.05	2
33685280	Nishizawa, 2021	Chronic pain Postherpetic neuralgia	Musculoskeletal pain Neuropathic pain	Binary Binary	Cases 191 and controls 282 Cases 89 and controls 282	Japanese Japanese	1.86E-07 1.86E-07	0 1
22956598	Peters, 2012	Chronic widespread pain	Musculoskeletal pain	Binary	Cases 1308 and controls 5791	EA	1.00E-05	10
33926923	Rahman, 2021	Chronic widespread pain	Musculoskeletal pain	Binary	Cases 6914 and controls 242,929	EA	5.00E-08	3
27670397	Reyes-Gibby, 2016	Severe pretreatment cancer pain	Cancer pain	Binary	Cases 148 and controls 810	EA	5.00E-08	0
29884837	Reyes-Gibby, 2018	Neuropathy	Neuropathic pain	Binary	Cases 130 and controls 913	EA	5.00E-08	4
28081371	Sanders, 2017	Temporomandibular disorder	Orofacial pain	Binary	Cases 769 and controls 9384	Hispanic; Latino	5.00E-08	1 in overall; 2 in females
26138065	Schneider, 2015	Paclitaxel induced peripheral neuropathy	Cancer pain	Binary	Cases 727 and controls 843	EA; AA; others	5.00E-05	1 in EA; 1 in AA
30431558	Smith, 2019	Temporomandibular disorder	Orofacial pain	Binary	Cases 999 and controls 2031	EA; AA; others	5.00E-08	1 in overall; 2 in females; 1 in males
29278617	Sucheston-Campbell, 2018	Paclitaxel induced peripheral neuropathy	Cancer pain	Binary	Cases 178 and controls 1230	EA; AA	5.00E-08	0
30261039	Suri, 2018	Chronic back pain	Musculoskeletal pain	Binary	Cases 29,531 and controls 128,494	EA	5.00E-07	4
33729212	Suri, 2021	Chronic back pain	Musculoskeletal pain	Binary	Cases 49,182 and controls 51,629	EA	5.00E-08	0
29502940	Takahashi, 2018	Opioid analgesia	Pain treatment response	Continuous	355	Japanese	Fisrt and second stage *P* < 0.05; final stage Q < 0.05	2
31127053	Tang, 2019	Diabetic peripheral neuropathy	Neuropathic pain	Binary	Cases 4384 and controls 784	EA	1.00E-05	13
32587327	Tsepilov, 2020	Genetic components of multisite chronic pain	Musculoskeletal pain	Continuous	265,000	EA	1.30E-08	9
31903573	van Reij, 2020	Chronic postoperative pain	Postoperative pain	Binary	Cases 34 and controls 296	EA	1.00E-05	11
34854908	Veluchamy, 2021	Neuropathic pain	Neuropathic pain	Binary	In stage 1, cases 1244 and controls 2832, In stage 2, cases 3268 and controls 425,657	EA	5.00E-08	1
28051079	Warner, 2017	Chronic postoperative neuropathic pain	Postoperative pain	Binary	Cases 109 and controls 504	Not report	1.00E-05	4
34975738	Winsvold, 2021	Idiopathic polyneuropathy	Neuropathic pain	Binary	Cases 2093 and controls 445,256	EA	5.00E-08	0
22020760	Won, 2012	Oxaliplatin-induced chronic peripheral neuropathy	Cancer pain	Binary	Cases 39 and controls 57	Korean	1.00E-05	0
30277654	Yokoshima, 2018	Opioid analgesia	Pain treatment response	Discrete	71	Japanese	5.00E-08	2

AA, African American; EA, European ancestry; PMID, publication PubMed ID.

**Figure 3.**
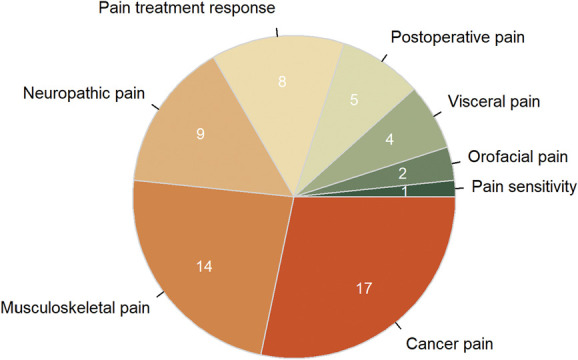
The number of published studies on different pain conditions included in this review. The total number of studies in this figure is 60 rather than the total number of articles (n = 57), as 3 articles investigated different pain phenotypes that are not in the same category.

We followed the 11th revision of the International Classification of Diseases (ICD-11) of chronic pain (Fig. 3) to define the phenotypes reported in the identified articles. Articles were categorized based on anatomical sites. Cancer pain was investigated most (n = 17), followed by musculoskeletal pain (n = 14) and neuropathic pain (n = 9). Pain sensitivity is the least investigated phenotype with only 1 article.

Below is a summary of the included studies focusing on overlapping findings between the studies. Details of all loci meeting the inclusion criteria are provided in Supplementary Data S1 (available as supplemental digital content at http://links.lww.com/PAIN/B808). Phenotype definitions were added in each general section (cancer pain, musculoskeletal pain, neuropathic pain, postoperative pain, visceral pain, and orofacial pain), mostly from ICD-11. The definitions of pain sensitivity and pain treatment response were not added because the diverse phenotypes were used in different studies and lacked an official definition. Definitions used in the included studies may sometimes differ from the official definitions, the definitions used in the included articles can be found in Table S4, available as supplemental digital content at http://links.lww.com/PAIN/B808.

### 3.3. Cancer pain

“Chronic cancer-related pain is chronic pain caused by primary cancer itself or metastases (chronic cancer pain) or its treatment (chronic postcancer treatment pain).”^7^

#### 3.3.1. Severe pretreatment pain

Reyes-Gibby et al.^109^ conducted a GWAS on severe pretreatment pain in untreated cancer patients to exclude pain associated with cancer treatment. They identified 1 genome-wide significant intergenic variant near *OR13G1/OR6F1* in the combined analysis of the discovery and replication (n = 958) phase.

#### 3.3.2. Chemotherapy-induced peripheral neuropathy

Chemotherapy-induced peripheral neuropathy (CIPN) is caused by oral or intravenous chemotherapy. Common chemotherapy agents that cause peripheral neuropathy include taxanes (paclitaxel and docetaxel), platinum-based drugs (cisplatin and oxaliplatin), vinca alkaloids (vincristine), thalidomide, and proteasome inhibitors (bortezomib).

Baldwin et al.^5^ conducted the first GWAS on CIPN in patients receiving paclitaxel treatment in the CALGB 40101 cohort (n = 855). This study identified 11 suggestively significant loci associated with paclitaxel-induced peripheral neuropathy. Seven GWASes^2,18,44,62,64,116,123^ were performed to identify genetic variants associated with taxane-induced peripheral neuropathy. Only 1 genome-wide significant association (an intronic locus in *TMEM150C*) was identified in the study by Adjei et al.^2^ (n = 692, but this locus could not be replicated in the same study).

There are 8 GWASes for other chemotherapies, including platinum-induced peripheral neuropathy,^26,57,144^ vincristine-induced peripheral neuropathy,^24,69^ and bortezomib-induced peripheral neuropathy.^13,38,77^ Only in the analysis focusing on vincristine-induced peripheral neuropathy (n = 321),^24^ a genome-wide significant intergenic region (*LOC100996325/CEP72*), was identified. All the other studies reported only suggestively significant results.

#### 3.3.3. Acute postradiation therapy pain

Lee et al.^65^ conducted a GWAS on postradiotherapy pain in breast cancer patients (n = 1112). They identified 3 suggestively significant loci (an intronic variant in *ABCC4*, an intergenic variant near *LINC01203*/*EGFL6*, and a noncoding transcript variant in *RFFL*) without a replication cohort.

#### 3.3.4. Overlap between studies on cancer pain

Although only a limited number of genome-wide significant hits have been reported, the studies focusing on CIPN reported 3 suggestively associated loci that showed overlap between studies investigating different drugs: an intergenic region near *LRP12/ZFPM2*, an intronic locus in *FGD4*, and an intergenic region near *LINC00290*. Interestingly, the gene *LRP12* is involved in the internalization of lipophilic molecules, and *FGD4* is known to cause a peripheral nervous system disorder (Charcot–Marie–Tooth disease).

### 3.4. Musculoskeletal pain

Chronic musculoskeletal pain is defined as *chronic pain arising from musculoskeletal structures such as bones or joints*.^105^

#### 3.4.1. Chronic back pain

Back pain is the leading cause of disabling conditions worldwide.^41^ Back pain may appear as a new (acute) episode or it develops as persistent (chronic) back pain if individuals fail to recover from acute episodes.^58^ The estimated heritability of back pain ranges from 30% to 68%, indicating a genetic predisposition.^6,56,75,99^

Four GWASes have been conducted on (chronic) back pain. In 2018, Suri et al.^124^ performed the first GWAS focusing on self-reported chronic back pain by combining 2 cohorts (the UKB and the CHARGE consortium) in a meta-analysis (n = 158,025). Four loci were identified: 3 in intronic regions (*DCC*, *DIS3L2*, and *SOX5*) and 1 in the intergenic region near *CCDC26*/*GSDMC*. A later study by Freidin et al.^35^ also used the UKB cohort for a GWAS on back pain (n = 350,000); the main phenotype definition difference with Suri et al.^124^ is that they had no limitations on the duration of back pain. Besides *CCDC26/GSDMC* and *SOX5*, they identified 3 additional loci, 2 in the intronic region of *C8orf34* and *HTRA1* and 1 in the intergenic region near *SPOCK2/CHST3*.

In another study on chronic back pain,^125^ no genome-wide significant loci were identified using samples from eMERGE Phase 3 (eMERGE3) and Geisinger (n = 100,811, in total). However, this study replicated results from the 2 studies described above. The variants rs12310519 (*SOX5*) and rs7814941 (*CCDC26/GSDMC*) were replicated (*P* = 0.011, *P* = 0.005, respectively), with a very similar magnitude and direction of effect as the initial studies. The previously reported association of rs3180 (near *SPOCK2/CHST3*) was not statistically significant in this study but had a similar magnitude and direction of effect.

The genetic architecture for chronic pain might be sex‐specific as the prevalence of chronic pain is sex‐related (ie, female patients are more frequently affected), even after adjustment for many socioeconomic, demographic, and clinical risk factors (hormone profiles).^115,119^ A sex-stratified GWAS on chronic back pain in the UKB identified 2 loci in men (n = 202,077) and 7 loci in women (n = 237,754).^36^ One of the loci identified in men (rs1678626 in the intronic region of *SPOCK2*) was replicated in the same study. One locus identified in women also showed a significant *P* value in the replication (rs62327819 in the intronic region of the *SLC10A7* gene [*P* = 0.0048]) but showed an opposite direction of effect.

#### 3.4.2. Chronic knee pain

Knee pain can be localized (use of 1 or 2 fingers to point to a specific location), regional (use of all of the fingers or the whole hand to cover a more extensive region), or diffuse/unable to identify pain as localized or regional in nature.^129^ Genetic studies have focused on knee pain caused by osteoarthritis and less on knee pain in general.^85^ The only GWAS that we identified studying chronic knee pain was performed by Meng et al.^85^ In their study, 2 genome-wide significant loci associated with general knee pain were identified using data from the UKB (n = 171,516): one variant in the 5′-UTR region of *GDF5* and the other is in the intergenic region near *KIF12/COL27A1*. These results were supported by 2 independent replication cohorts of knee osteoarthritis in the same study.

#### 3.4.3. Chronic shoulder and neck pain

Neck or shoulder pain is often described as a single entity^112^ as pain in the cervicobrachial area with shared etiology,^89,118^ and lesions in the neck can lead to pain in the shoulder and vice versa.^32^ Also for this phenotype, only 1 GWAS was identified.^86^ In this study, 3 loci were identified to be associated with neck and shoulder pain in the UKB (n = 203,309): 2 intergenic variants (1 near *FOXP2* and 1 near *CA10/LINC01982*) and 1 variant in the noncoding RNA *LINC01572*. A replication included in the same article showed a weak association for the *FOXP2* and *LINC01572* loci in the GS:SFHS cohort but not in the TwinsUK cohort. All 3 loci showed genome-wide significance in the joint meta-analysis.

#### 3.4.4. Chronic widespread pain, fibromyalgia, and multisite chronic pain

Chronic widespread pain (CWP) is defined as “*diffuse musculoskeletal pain in at least 4 of 5 body regions and at least 3 or more body quadrants (as defined by upper-lower/left-right side of the body) and axial skeleton (neck, back, chest, and abdomen)*.”^94^ Fibromyalgia is considered more severe and at the end of the spectrum of CWP. Fibromyalgia is often accompanied by sleep disorders, cognitive dysfunction, and somatic symptoms,^94^ but CWP and fibromyalgia syndrome are sometimes used interchangeably.

Two early articles conducted a GWAS on chronic widespread pain (n = 7099)^106^ and fibromyalgia (n = 503),^25^ respectively. However, both articles were low in statistical power, with only suggestively significant results. More recently, Rahman et al.^108^ conducted the largest GWAS on CWP using the UKB as a discovery cohort (n = 249,843) and 6 independent replication cohorts (n = 57,257). Three genome-wide significant loci were identified; 2 were in the intronic region of *RNF123* and *ATP2C1* and 1 was in the 3′-UTR region of *COMT*. Only the *RNF123* locus was successfully replicated.

Two studies performed a GWAS on the number of localized chronic pain sites in the UKB (see Table S4, available as supplemental digital content at http://links.lww.com/PAIN/B808 for more details concerning the phenotype definition). These 2 studies unraveled a shared genetic background between CWP and multisite chronic pain (MCP). Johnston et al.^53^ identified 39 genome-wide significant loci with diverse gene functions (n = 387,649). Many identified genes were implicated in nervous system development, neural connectivity, and neurogenesis. In a later sex-stratified analysis for MCP,^54^ 5 loci in men (n = 178,556) and 10 loci in women (n = 209,093) were identified, respectively. Although Rahman et al.^108^ and Johnston et al.^53^ used the same cohort (UKB) for their studies, the results differ because they used a slightly different phenotype definition for cases and controls. Johnston et al.^53^ also showed that the genetic correlation between CWP and MCP was high (rg = 0.83, *P* = 2.45 × 10^−54^), and most SNPs showed consistent effect size and directions of effect between MCP and CWP.

Tsepilov et al.^132^ investigated genetic factors underlying MCP at 4 locations (back, neck/shoulder, hip, and knee) using a principal component analysis to reduce the heterogeneity in phenotypes (see Table S4, available as supplemental digital content at http://links.lww.com/PAIN/B808 for more details concerning the phenotype definition). They identified 9 genome-wide significant loci, and 6 were replicated in the replication phase of this study (a variant in the 5′-UTR region of GDF5, 2 intronic variants in *EXD3* and *FOXP2,* respectively; 2 exonic variants in *SLC39A8* and *ECM1,* respectively; a variant in the 3′-UTR region of *AMIGO3/GMPPB*).

#### 3.4.5. Complex regional pain syndrome

*“Complex regional pain syndrome (CRPS) is a type of chronic primary pain characterized by pain in a regional distribution that usually starts in an extremity after trauma, and further characterized by signs indicating autonomic and inflammatory changes.”*^94^ The etiology of CRPS remains largely unknown, but evidence suggests genetic predispositions in the HLA region.^23,136^

In a GWAS performed by Janicki et al.,^50^ no genome-wide significant variants were identified, and none of the previously reported SNPs in the HLA region remained significant after multiple-testing correction. The top associated SNP was an intronic variant in *NAV3* (*P* = 0.0003) in the discovery phase (n = 460). Although this locus failed to pass the suggestively significant threshold, it is reported as part of this review because it overlaps with the top locus in a GWAS on postoperative pain^135^ (See paragraph 3.7).

#### 3.4.6. Chronic pain mixed phenotypes

Nishizawa et al.^98^ conducted a GWAS on chronic pain with mixed phenotypes (n = 473), including postherpetic neuralgia, lower back pain, hernia of intervertebral disk, spinal canal stenosis, postoperative pain, neck pain, and others. They were unable to identify any (suggestively) significant hits.

#### 3.4.7. Overlap between studies on musculoskeletal pain phenotypes

Genes reported more than once in GWASes on back pain are *SOX5*, *C8orf34*, *SPOCK2*, *CCDC26/GSDMC*, and *DCC*. These genes functionally link to chondrogenesis (*SOX* genes family)^73^; cartilage,^148^ osteoarthritis,^111^ and lumbar disc degeneration (*CCDC26/GSDMC*)^8^; and nociceptive pathways (*DCC*).^145^ However, the function of some genes/loci (eg, *C8orf34*) and how they are involved in back pain are still unexplained.

Several genes/loci have been reported more than once in CWP, fibromyalgia, and MCP, including *EXD3*, *SLC39A8*, *AMIGO3*/*GMPPB*/*RNF123*, *C6orf106*, *FAF1*, *SLC24A3*, and *LINC01065*/*LINC00558*. In addition, 2 SNPs associated with MCP from different studies were in LD (*r*^2^ = 0.958), rs3737240 (in the exon of *ECM1*),^132^ and rs59898460 (in the intergenic region near *FALEC*/*ADAMTSL4*).^53,54^ The functions of the reported genes include cell-cycle progression (*EXD3*, *RNF123*), onset of inflammation (*SLC39A8*), brain development (*AMIGO3*), apoptosis (*FAF1*), and intracellular calcium homeostasis and electrical conduction (*SLC24A3*).

The following genes were reported for more than 1 musculoskeletal pain phenotype: *DCC*, *FALEC*/*ADAMTSL4*, *CA10*/*LINC01982*, *FOXP2*, and *GDF5*. Relevant functions include patterning of the developing nervous system (*ADAMTSL4*), development and maintenance of synapses (*CA10*),^122^ brain development, neurogenesis, signal transmission and synaptic plasticity (*FOXP2*),^138^ and overlap with genes associated with osteoarthritis (*GDF5*).

The overlap within musculoskeletal pain should be interpreted carefully because many musculoskeletal GWASes included UK Biobank (UKB) samples as (part of the) discovery cohort. Therefore, the overlapping genes/loci might be the result of the sample overlap. For instance, multisite chronic pain GWASes include UKB participants reporting all kinds of chronic pain, including back pain. However, back pain was also investigated as an individual phenotype in another GWAS. Besides overlap in cohorts, the overlapping finding of sex-stratified and sex-unstratified analyses on the same phenotype are also reported in this review.

### 3.5. Neuropathic pain

Neuropathic pain is defined as “*pain caused by a lesion or disease of the somatosensory system*.”^52^ The etiology of neuropathic pain can be diverse, including metabolic disease (eg, diabetic neuropathy), surgery or trauma, infections (eg, shingles and HIV), exposure to chemotherapy, or unknown etiology (eg, idiopathic neuropathies).

#### 3.5.1. Diabetic neuropathic pain

Diabetic peripheral neuropathy is the most common cause of neuropathy,^107^ with 21% of patients with diabetes suffering from it.^1^ The first GWAS on diabetic peripheral neuropathy was conducted in the GoDARTS cohort by Meng et al.^84^ Cases were defined as type 2 diabetic individuals if they had at least 1 prescription history of any of the following medicines for diabetic peripheral neuropathy: duloxetine, gabapentin, pregabalin, capsaicin cream/patch, and lidocaine patch (see Table S4, available as supplemental digital content at http://links.lww.com/PAIN/B808 for more details concerning the phenotype definition). One suggestively significant intergenic region near *GFRA2/DOK2* was found to be associated with diabetic peripheral neuropathy (n = 3063).

In the same year, Meng et al.^83^ conducted a GWAS in the same cohort with a more stringent case definition: cases were defined as type 2 diabetic patients who have a minimum of 2 prescriptions of the 5 medicines that were also included in the previous study (see above). This analysis led to the identification of 1 suggestively significant locus in the intronic region of *ZSCAN20*. In the sex-stratified analysis, this locus remained suggestively significant in women (n = 1730), and another intergenic region near *ABRA/ANGPT1* passed the suggestively significant threshold in men (n = 2491).

Tang et al.^128^ conducted a GWAS on diabetic peripheral neuropathy in 2 diabetic trials (n = 5168 in total). An intergenic region near *SCN7A/XIRP2* passed the genome-wide significant threshold and was successfully validated. The minor allele at this locus was associated with a higher expression of the adjacent gene *SCN2A* in the tibial nerve. Two additional intronic variants in *NTRK3* and *THEG5* passed the suggestively significant threshold in the discovery phase and almost reached genome-wide significance in the meta-analysis.

#### 3.5.2. Neuropathy/neuropathic pain

As diverse types of peripheral neuropathy can cause neuropathic pain, we also included GWASes on neuropathy in this review, although not all neuropathy patients have pain experience. Therefore, the genetic association findings in neuropathy might not directly link to pain. Reyes-Gibby et al.^110^ conducted a GWAS on neuropathy in untreated head and neck cancer patients (n = 1043) defined by ICD codes. They identified 4 loci passing the genome-wide significance threshold (an intergenic region in *KNG1/EIF4A2*, an upstream region in *PCP2*, and 2 intronic variants in *RORA* and *SNX8*, respectively), but no validation was done as part of this study. Veluchamy et al.^137^ conducted a 2-stage analysis for neuropathic pain. The first stage consisted of a meta-analysis of the GoDARTS (n = 803) and GS:SFHS cohorts (n = 3273). Both cohorts used the Brief Pain Inventory questionnaire and Douleur Neuropathique 4 Questions to define neuropathy. In stage 2, they combined the results of stage 1 with UKB data (n = 428,925). For these participants, a proxy phenotype was used to define neuropathy. In stage 1, one intergenic region near the *EPHA3* gene showed genome-wide significance. In stage 2, a locus near *SLC9A7P1* showed genome-wide significance. The *EPHA3* locus identified in stage 1 and another intronic variant in the *CAB39L* gene were close to genome-wide significance.

Winsvold et al.^143^ conducted a GWAS on idiopathic polyneuropathy in the HUNT and UKB cohorts (n = 63,351 in total). Only in the meta-analysis, 2 genome-wide significant loci were identified (1 intronic variant in *B4GALNT3 and* 1 intergenic variant near *NR5A2/LINC01221*). They aimed to validate 5 previously identified variants in 5 genes that are reported to be associated with related (polyneuropathy) phenotypes; *PRPH* associated with nerve conduction,^9^ *CEP72* and *VAC14* associated with CIPN,^24,44^ *IL2RA* associated with drug-induced peripheral neuropathy,^66^ and *XIRP2* associated with diabetic peripheral neuropathy.^128^ Unfortunately, none of these 5 variants were successfully validated in this study (FDR-corrected *P* value < 0.05 for 5 tests). They also selected 69,887 variants near 175 genes related to monogenic forms of polyneuropathy (eg, hereditary neuropathy, familial dysautonomia) to validate. None of these variants remained significant after multiple testing correction (FDR-corrected *P* value < 0.05 for 69,887 tests).

#### 3.5.3. Sciatica

The typical symptom of sciatica is sciatic pain or lumbar radicular pain, and it is usually caused by a common low back disorder, eg, lumbar disc herniation.^121^ Lemmela et al.^67^ conducted a GWAS on sciatica using a meta-analysis of 2 discovery cohorts (n = 3962 in total). In the discovery phase, they identified 2 genome-wide significant variants (in the intronic region of *MYO5A* and *NFIB*, respectively). Only the variant in *MYO5A* was replicated in an independent cohort (n = 19,265).

#### 3.5.4. Postherpetic neuralgia

Nishizawa et al.^98^ conducted a GWAS on postherpetic neuralgia (n = 371). One intronic SNP in the *ABCC4* gene showed a genome-wide significant association with postherpetic neuralgia using an additive model.

#### 3.5.5. Drug-induced peripheral neuropathy

Leger et al.^66^ identified 5 suggestively significant loci associated with stavudine and didanosine-induced peripheral neuropathy (n = 254), which were an intronic variant in *ADAMTS2*, an exonic variant in *KRR1*, an intergenic variant near *MIR8054/LUZP2*, a variant in the 3′-UTR region of *SASH1*, and an intergenic variant near *SLCO3A1/ST8SIA2*.

#### 3.5.6. Overlap between studies on neuropathic pain phenotypes

Based on the *P* value thresholds used in the articles, we could not identify overlapping genes/loci between the GWAS studies on neuropathic pain phenotypes.

### 3.6. Visceral pain

Chronic visceral pain is chronic pain originating from internal organs of the head or neck region or of the thoracic, abdominal, or pelvic region.^131^

#### 3.6.1. Dysmenorrhea pain

Dysmenorrhea pain is an intense and often disabling abdominal or pelvic pain during every menstrual cycle, and it can be primary or secondary (eg, to endometriosis). Three GWASes have been conducted on dysmenorrhea pain, and each study included participants from different populations: European (n = 11,891),^55^ Chinese (n = 5324),^68^ and Japanese (n = 11,348).^46^ One shared intergenic region was identified in these 3 studies: *TSPAN2/NGF.* An intronic variant in *ZMIZ1* was only identified in the Chinese study, and an intergenic region near *IL1A/IL1B* was only identified in the Japanese study. The *IL1A* signal might reflect endometriosis because this locus was previously reported to be associated with endometriosis.^114^

#### 3.6.2. Constant-severe pain in chronic pancreatitis

Dunbar et al.^28^ conducted a GWAS on constant-severe pain in chronic pancreatitis (n = 1357).^92^ One suggestively significant locus was identified in the intronic region of *SGCZ* without replication.

### 3.7. Postoperative pain

Postoperative pain can be acute or chronic. “*Chronic Postoperative pain develops or increases in intensity after a surgical procedure and persists beyond the healing process,* ie, *at least 3 months after the surgery*.”^117^

Kim et al.^61^ conducted the first GWAS on acute postoperative pain using 2 phenotypes separately, ie, the maximum postoperative pain rating and postoperative pain onset time (n = 112 for both phenotypes). However, no significant loci were identified after correcting for multiple comparations. In another study, Cook-Sather et al.^21^ identified 2 suggestively significant loci (an intergenic variant near *CDC5L/LOC105375075* and an upstream variant of *LOC105375075*) associated with acute postoperative pain scores (n = 277).

Two additional studies investigated chronic postpostoperative pain. Warner et al.^139^ identified 4 suggestively significant loci associated with postoperative neuropathic pain in post–total joint replacement patients (n = 613). To define neuropathic pain cases, a 7-item questionnaire was used to describe the nature of pain. The top hit in the meta-analysis was an intronic variant in the *PRKCA* gene (*P* = 1.65 × 10^−5^). The study of van Reij et al.^135^ found 11 loci suggestively significantly associated with chronic postoperative pain measured by the numeric rating scale (NRS) (n = 330). Only 1 intronic variant in *NAV3* was replicated in an independent cohort (*P* = 0.009).

No overlap was found between the studies that investigated postoperative pain.

### 3.8. Orofacial pain

The most investigated orofacial pain is temporomandibular disorder (TMD), which can be primary or secondary to persistent inflammation, structural changes (such as osteoarthritis or spondylosis), injury, or nervous system diseases. Sanders et al.^113^ conducted the first GWAS on TMD in participants of Hispanic/Latino ancestry (n = 10,153). A stringent case definition was applied, ie, reporting pain in both face and jaw joint, but information on symptoms duration was not available. One genome-wide significant locus in the intronic region of the *DMD* gene was identified. Unfortunately, this SNP was not genotyped in the replication cohorts. Another suggestively associated intergenic variant near *PPP1R9B/SGCA* was replicated in 1 replication cohort. This study also performed a sex-stratified analysis and identified 2 genome-wide significant loci in women (an intergenic variant near *B3GLCT/RXFP2* and an intronic variant in *BAHCC1*); only the variant in *BAHCC1* was replicated among women in the meta-analysis of this study. This article does not mention whether they conducted the analysis in men only.

Smith et al.^120^ investigated genetic variants associated with examiner-verified chronic TMD (see Table S4, available as supplemental digital content at http://links.lww.com/PAIN/B808 for more details concerning the phenotype definition) in the OPPERA cohort (n = 3030), and this cohort was one of the replication cohorts in the study described above.^113^ They identified one genome-wide significant variant (in the intergenic region near *OTUD4/LINC02266*) in the analysis including men and women. A sex-stratified analysis identified 2 loci in women (an intronic variant in *SFRP1* and the same intergenic variant as in the analysis including all subjects) and 1 in men (an intergenic region near *LINC01210/CLDN18*). However, no SNPs were replicated in the meta-analysis of 7 independent cohorts after applying Bonferroni correction.

Similarly, for orofacial pain, no overlap was found in the reported genes.

### 3.9. Pain sensitivity

Fontanillas et al.^34^ conducted the first GWAS on pain sensitivity. Two phenotypes were used to measure pain sensitivity: a pain questionnaire (n = 25,321) and a cold pressor test (n = 6853). In the GWAS, using the first phenotype, they identified 1 genome-wide significant locus in the intronic region of *EIPR1* and 2 suggestively associated loci (an intergenic region near *VAPA*/*LINC01254* and one intronic variant in *NALCN*). The GWAS using the cold pressor test as phenotype led to the identification of 1 suggestively significant locus in the intronic region of *PITPNC1*. The reported loci for each of the 2 phenotypes were not associated with the other phenotype. In addition, Fontanillas et al.^34^ also validated the previously reported *MC1R* variants in their study. Variants in this gene were associated with red hair and modulate pain sensitivity, especially in women.^17,43,149^ Three *MC1R* variants were tested for association with increased self-perceived pain sensitivity, and the most statistically significant variant was rs1805007 (*P* value = 5.10E-03).

### 3.10. Pain treatment responses

#### 3.10.1. Nonsteroidal anti-inflammatory drugs

Kim et al.^61^ conducted a GWAS on analgesic onset time after ketorolac administration (n = 112). They identified 1 genome-wide significant locus in the upstream region of *ZNF429*.

#### 3.10.2. Opioids

Galvan et al.^37^ conducted a GWAS on pain relief measured by an 11-point numerical rating scale in patients receiving opioid treatment (morphine, oxycodone, and fentanyl) (n = 438). They split the cohort into 2 groups and applied a 2-stage analysis. Eight suggestively significant loci were identified in stage 1, but only 1 intergenic region near *RHBDF2/CYGB* showed significance in the combined analysis of stages 1 and 2.

Cook-Sather et al.^21^ performed a GWAS using total postoperative morphine requirement as phenotype. They identified 3 suggestively significant loci in Europeans (n = 277) and 9 in African Americans (n = 241). The top SNP from the analysis including Europeans, rs795484 in the intronic region of *TAOK3*, was replicated in a small replication cohort (n = 75). This variant was also associated with postoperative pain scores in the same study (see above under 3.7 postoperative pain) in both Europeans (*P* < 5E-5) and African Americans (*P* < 0.01).

Nishizawa et al.^96^ conducted a GWAS on opioid analgesic (fentanyl) requirements during the 24-hour postoperative period (n = 355). They divided 1 cohort into 3 groups for a 3-stage analysis: SNPs that showed *P* values of <0.05 in one stage were selected as candidate SNPs for the next stage. In the final stage, SNPs with Q of <0.05 (the Q-values of false discovery rate for multiple testing correction) were considered significant. This study identified 1 significant intergenic variant near *METTL21A/LINC01857*. Three additional studies applied the same method to investigate genetic variants associated with opioid analgesia. These studies identified 1 exonic variant in *LAMB3*,^87^ an intronic variant in *SLC9A9*, a variant in the 5′-UTR region of *TMEM8A*,^97^ and 2 intergenic regions near *C3orf38/EPHA3* and *LOC389602/LOC285889*.^126^ In addition, Yokoshima et al.^146^ identified 2 genome-wide significant loci (one intronic variant in *ABAT* and an intergenic region near *DAZL*/*PLCL2*), which were associated with pain decrease corresponding to opioid analgesics but without replication (n = 71).

No overlap was found between the genes identified for pharmacological pain treatment outcomes.

### 3.11. Follow-up research

#### 3.11.1. Overlap between different pain phenotypes

Besides checking the overlap genes/loci in the same pain phenotype or category (as described above), we also investigated the overlap between the reported loci in different pain categories (see Table 2 for a summary). Thirty loci were reported in at least 2 studies and covered a wide range of phenotypes. *DCC* is the most reported gene, with 4 studies on musculoskeletal pain and 1 on CIPN. Two gene families were reported frequently, ie, the ephrin receptor subfamily of the protein-tyrosine kinase family (*EPHA3* and *EPHA4*) and the *SOX* (SRY-related HMG-box) family of transcription factors (*SOX5* and *SOX6*). *EPHA3* was reported in 1 opioid analgesia study and 1 neuropathic pain study, and *EPHA4* was reported in 1 CIPN and 1 chronic postoperative pain study. *SOX5* was reported in 3 chronic back pain studies, *SOX6* was reported in 1 CIPN study, and 1 multisite chronic pain study. Many genes are implicated in neurological functions (Table 2).

**Table 2:** Loci reported more than once from all included articles.

Mapped genes/loci region	Gene functions	SNP	Outcome	PMID	Comments
*DCC*	Nociceptive pathways	rs4384683 rs62098013 rs72922230 rs17748074 18:50442591_TTTC_T	Chronic back pain Multisite chronic pain Chronic back pain CIPN Multisite chronic pain	30261039 31194737 33021770 28317148 33830993	* * * † *
*FALEC; ADAMTSL4*	Nervous system development (*ADAMTSL4*)	rs59898460 rs367563576 rs59898460	Multisite chronic pain Chronic back pain Multisite chronic pain	31194737 33021770 33830993	* * † * †
*CA10; LINC01982*	Brain development (*CA10*)	rs12453010 rs11079993 rs11079993	Shoulder and neck pain Multisite chronic pain Multisite chronic pain	32246137 33830993 31194737	* * † *
*EXD3*	Cell-cycle progression	rs73581580 rs73581580 rs73581580	Multisite chronic pain Genetic components of chronic musculoskeletal pain Multisite chronic pain	31194737 32587327 33830993	* * * †
*FOXP2*	Brain development	rs12537376 rs2049604 rs12705966	Multisite chronic pain Shoulder and neck pain Genetic components of chronic musculoskeletal pain	31194737 32246137 32587327	* * *
*LRP12; ZFPM2*	Internalization of lipophilic molecules (*LRP12*)	rs2941627 rs3110366 rs3110290	CIPN CIPN CIPN	22843789 32562552 28611204	
*TSPAN2; NGF*	Regulation of cell development, activation, growth and motility (*TSPAN2*), sensory neurons growth and differentiation (*NGF*)	rs7523831 rs12030576 rs7523086	Dysmenorrhoea pain Dysmenorrhoea pain Dysmenorrhoea pain	28447608 29855537 27454463	
*SLC39A8*	Inflammation	rs13135092 rs13107325 rs13135092	Multisite chronic pain Genetic components of chronic musculoskeletal pain Multisite chronic pain	31194737 32587327 33830993	* * * †
*SOX5*	Chondrogenesis	rs12310519 rs12310519 rs12308843	Chronic back pain Chronic back pain Chronic back pain	30261039 30747904 33021770	* * * †
*ABCC4*	Prostaglandins transportation	rs4584690 rs4773840	Acute post-radiation therapy pain Postherpetic neuralgia	31196165 33685280	
*LINC01065; LINC00558*	Not known	rs1443914 rs34003284	Multisite chronic pain Multisite chronic pain	31194737 33830993	* * †
*RNF123; AMIGO3; GMPPB*	Brain development, synapse assembly (*AMIGO3*), cell cycle progression (*RNF123*)	rs7628207 rs1491985 rs7628207	Genetic components of chronic musculoskeletal pain Chronic widespread pain Multisite chronic pain	32587327 33926923 31194737	* * ‡ *
*C6orf106*	Inflammation	rs6907508 rs151060048	Multisite chronic pain Multisite chronic pain	31194737 33830993	* * †
*C8orf34*	Not known	rs1865442 rs7834973	Chronic back pain Chronic back pain	30747904 33021770	* * †
*CCDC26; GSDMC*	Lumbar disc degeneration	rs7814941 rs7833174	Chronic back pain Chronic back pain	30747904 30261039	* *
*EPHA3*	Nervous system development	rs13093031 rs112990863	Opioid analgesia Neuropathic pain	29502940 34854908	
*MIR4268; EPHA4*	Nervous system development (*EPHA4*)	rs17348202 rs10194315	CIPN Chronic postoperative pain	23776197 31903573	
*FAF1*	Apoptosis	rs10888692 rs35072907	Multisite chronic pain Multisite chronic pain	31194737 33830993	* * †
*FGD4*	Peripheral nerve pathophysiology	rs10771973 rs10771973	CIPN CIPN	22843789 32562552	
*GDF5*	Osteoarthritis	rs143384 rs143384	Chronic knee pain Genetic components of chronic musculoskeletal pain	31482140 32587327	* *
*LINC00290*	Not known	rs12501594 rs6552496	CIPN CIPN	34391895 28317148	
*NAV3*	Predominantly expressed in the nervous system	rs300501 rs118184265	CRPS Chronic postoperative pain	28025368 31903573	
*SLC24A3*	Electrical conduction	rs2424248 20:19709268_AAAAT_A	Multisite chronic pain Multisite chronic pain	31194737 33830993	* * †
*SOX6*	Chondrogenesis	rs4757366 rs61883178	CIPN Multisite chronic pain	28611204 31194737	*
*SPOCK2*	Neurogenesis	rs1678626 rs3180	Chronic back pain Chronic back pain	33021770 30747904	* † *
*FCGBP*	Maintenance of the mucosal structure	rs234348 rs17796312	Opioid analgesia Chronic widespread pain	24909733 22956598	
*LOC102546299; LINC01947*	Not known	rs7734804 rs10515902	Post-operation neuropathic pain Opioid analgesia	28051079 24909733	
*MIR4422HG; LINC01753*	Not known	rs1165472 rs12566055	CIPN Opioid analgesia	23776197 24909733	
*GPD2*	Calcium ion binding and glycerol-3-phosphate dehydrogenase activity	rs298235 rs13421094	Postoperation neuropathic pain Opioid analgesia	28051079 21622719	
*SP4*	DNA-binding transcription factor activity	rs73271865 rs7798894	Temporomandibular disorder Multisite chronic pain	28081371 31194737	*

* Studies included the UK Biobank (UKB) as their study cohort, and the phenotype definition is based on the pain questionnaires in the UKB (Category 100048).

† Sex-stratified analysis.

‡ This variant was identified by checking linkage disequilibrium rather than checking gene symbol overlap.

CIPN, chemotherapy-induced peripheral neuropathy; CPRS, complex regional pain syndrome; PMID, publication PubMed ID.

SNPs in linkage disequilibrium were summarized in Table S8, available as supplemental digital content at http://links.lww.com/PAIN/B808. Except for 2 additional SNPs associated with MCP (see 3.4.7), all SNPs in linkage disequilibrium were identified by checking overlapping gene symbols.

#### 3.11.2. Overlap between genome-wide association study-identified pain genes and pain genetic databases

To check whether genes described in this review are associated with (other) pain phenotypes, we searched 2 pain genetic databases. The first is the Human Pain Genetics Database (HPGDB). Twenty-five genes/loci reported in pain GWASes were also reported in the HPGDB (Table 3). *COMT* is the most investigated candidate gene with 90 published articles, followed by *SUGCT* (n = 9) and *TSPAN2/NGF* (n = 9) (see Supplementary Data S2 for details of the genes identified in HPGDB, available as supplemental digital content at http://links.lww.com/PAIN/B808). Of the 25 overlapping genes/loci, 6 genes/loci were associated with more than 2 phenotypes in HPGDB (*COMT*, *OPRD1*, *IL1A*, *IL1B, TSPAN2/NGF*, *GDF5*), and 15 genes were associated with migraine.

**Table 3:** Overlapping genes between genes/loci from all included articles in this review and HPGDB.

Overlapping *genes*	GWAS phenotype [PMID]	Phenotypes in HPGDB	No. of articles in HPGDB
*COMT* *	Chronic widespread pain [33926923]	Analgesia; cancer pain; fibromyalgia; migraine; musculoskeletal pain; neuraxial pain; neuropathic pain; nociception; other clinical pain; Postoperative pain; temporomandibular disorder	90
*SUGCT*	CIPN [32562552]	Migraine	9
*TSPAN2; NGF*	Dysmenorrhoea pain [28447608], [29855537], [27454463]	Temporomandibular disorder, migraine	9
*ASTN2*	Multisite chronic pain [31194737]	Migraine	7
*IL1B*	Dysmenorrhoea pain [29855537]	Analgesia; cancer pain; migraine; musculoskeletal pain; neuraxial pain	7
*IL1A*	Dysmenorrhoea pain [29855537]	Migraine; neuraxial pain; nociception	5
*YTHDF2; OPRD1*	CIPN [28611204]	Postoperative pain for both genes; these phenotypes only reported in OPRD1: analgesia; nociception; temporomandibular disorder	5
*FALEC; ADAMTSL4*	Multisite chronic pain [31194737] [33830993], chronic back pain [33021770]	Migraine	3
*PLCE1*	Fibromyalgia [24582949]	Migraine	3
*SLC24A3*	Multisite chronic pain [31194737] [33830993]	Migraine	3
*GDF5*	Chronic knee pain [31482140], genetic components of chronic musculoskeletal pain [32587327]	Neuraxial pain; temporomandibular disorder	2
*GPATCH2L; ESRRB*	Diabetic neuropathic pain [31127053]	Temporomandibular disorder	2
*LINC01777*	Opioid analgesia [24909733]	Migraine	2
*TAC1*	CIPN [22020760]	Temporomandibular disorder	2
*ABCC4*	Acute postradiation therapy pain [31196165], postherpetic neuralgia [33685280]	Cancer pain	1
*CACNB2*	CIPN [22843789]	Migraine	1
*CDC5L; LOC105375075*	Acute postoperative pain [24909733]	Migraine	1
*CX3CL1*	CIPN [32562552]	Postoperative pain	1
*GABRB1*	CIPN [34391895]	Neuraxial pain	1
*IL1RN*	Dysmenorrhoea pain [27454463]	Neuraxial pain	1
*LINC00290*	CIPN [34391895], CIPN [28317148]	Migraine	1
*METTL21A; LINC01857*	Opioid analgesia [23183491]	Analgesia	1
*RSU1*	Chronic postoperative pain [31903573]	Migraine	1
*TAOK3*	Opioid analgesia [24909733]	Postoperative pain	1
*TPH1*	Chronic widespread pain [22956598]	Migraine	1

* Intergenic variants between *COMT* and *TXNRD2* in Human Pain Genetics Database were also included.

CIPN, chemotherapy-induced peripheral neuropathy; GWAS, genome-wide association study; HPGDB, Human Pain Genetics Database; PMID, publication PubMed ID.

**Figure 4.**
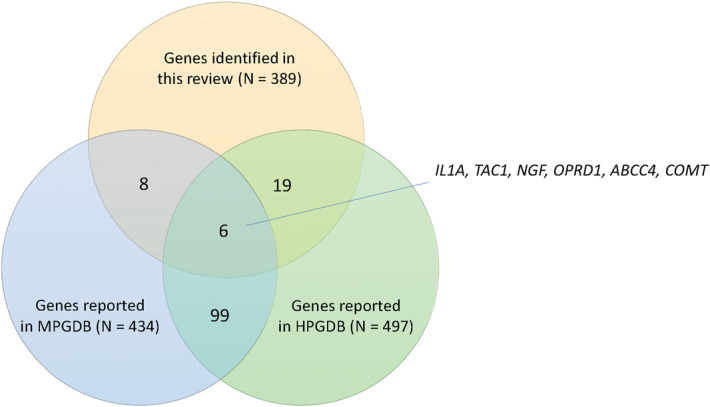
The number of (overlapping) genes reported from genome-wide association studies on pain included in this review, Human Pain Genetic Database (HPGDB), and mouse pain genetic database (MPGDB).

Besides the HPGDB, we also searched the mouse pain genetics database, which provides a repository of investigated genes in nociception, hypersensitivity, and analgesia in mice. Table 4 summarizes the overlapping genes between genes reported in this review and the mouse pain genetics database. Fourteen genes/loci could be found in the mouse pain genetics database. The functions of these genes are diverse (see Supplementary Data S3 for details, available as supplemental digital content at http://links.lww.com/PAIN/B808), but many overlapping genes are involved in neurological function, such as neurotransmitters (*COMT1*), neuromodulators (*TAC1*), neurotrophins (*EFNB2*, *GFRA2*, *NGF*), and synaptic scaffolding/vesicles (*DTNBP1*, *PPP1R9B*). An overview of the number of (overlapping) genes identified using different approaches/sources (GWAS findings, HPGDB, and the mouse pain genetics database) is depicted in Figure 4.

**Table 4:** Overlapping genes between genes/loci from all included articles in this review and mouse pain genetics database.

*Genes*	GWAS phenotype [PMID]	Nociception in mouse	Hypersensitivity in mouse	Analgesia in mouse
*ABCC4*	Acute postradiation therapy pain [31196165], postherpetic neuralgia [33685280]	Not tested	Mutant less sensitive	Not tested
*COMT*	Chronic widespread pain [33926923]	Mutant more sensitive	Not tested	Contradictory data
*DTNBP1*	CIPN [34391895]	Mutant less sensitive	Not tested	Not tested
*EFNB2*	Chronic back pain [33021770]	No difference	Mutant less sensitive	Not tested
*GFRA2*	Diabetic neuropathic pain [24974787]	Mutant more sensitive	Not tested	Not tested
*HDAC4*	Diabetic neuropathic pain [31127053]	Mutant less sensitive	Not tested	Not tested
*IL1 (IL1A,IL1B)*	Dysmenorrhoea pain [29855537]	Mutant less sensitive	Mutant less sensitive	Mutant less sensitive
*MAPK9*	CIPN [28611204]	No difference	Mutant less sensitive	Not tested
*NGF*	Dysmenorrhoea pain [28447608], [29855537], [27454463]	Mutant less sensitive	Not tested	Not tested
*OPRD1*	CIPN [28611204]	No difference	Mutant more sensitive	Contradictory data
*PMP22*	Chronic widespread pain [22956598]	Mutant less sensitive	No difference	Not tested
*PPP1R9B*	Temporomandibular disorder [28081371]	No difference	Not tested	Contradictory data
*PRKCA*	Postoperation neuropathic pain [28051079]	No difference	Mutant more sensitive	Not tested
*TAC1*	CIPN [22020760]	Mutant less sensitive	Contradictory data	Contradictory data

GWAS, genome-wide association study; PMID, publication PubMed ID.

## 4. Discussion

This review summarizes the findings from GWASes on pain and related phenotypes (nociception, neuropathy, and pain treatment response). In all GWAS studies included in this review, 32 overlapped loci were found between the studies, and some loci reported in GWASes also overlapped with candidate gene studies in humans and mice. Our study provides an overview of the identified and potential genetic risk factors for pain from GWAS findings. Our results suggest that multiple genetic risk factors involved in different functions can influence susceptibility to pain. Especially, many GWAS-identified genes and overlapping genes between included studies are implicated in neurological functions and inflammations. These functions are critical for pain development because chronic pain mainly arises from inflammation and nerve injury at the peripheral level and neuroplasticity at the central level.^12,101^

The involvement of genes related to neurological functioning meets our expectations because pain is mediated by processes in the nervous system regardless of the nature of pain.^39^ The most-reported locus is the *DCC* gene region. *DCC* plays several key roles in both central nervous system development^27^ and mature neuron survival and death.^51^ *DCC* might also be important for pain development because this gene is necessary for noxious stimuli localization in both mice and humans,^22^ and it is known to contribute to neuropathic pain^70,145^ and maladaptive responses (tolerance, dependence, and opioid-induced hyperalgesia) to opioids in mouse.^71^ Interestingly, several other repeatedly reported genes can also be linked to neurological mechanisms, eg, brain development and neuron functioning and development. This all stresses that the nervous system is indeed an important player in pain.

Besides neurological functions, the overlapping genes also suggest other possible mechanisms involved in pain, including inflammation. Inflammation events are highly relevant to pain because it plays a central role in the pathogenesis of chronic pain.^127^ In addition, some overlapping genes were implicated in diseases with pain as one of the symptoms. For instance, the genes found in chronic back pain are involved in chondrogenesis (*SOX5* and *SOX6*^73^) or lumbar disc degeneration (*CCDC26/GSDMC*^8^). Similarly, the *GDF5* locus found in knee pain is also involved in osteoarthritis.^133^

However, the function of some loci remains unclear because they were mapped to an intergenic region or nonprotein coding genes with unknown functions (such as *LINC01065* and *C8orf34*). Rather than influencing protein coding, these variants might regulate gene expression levels.^14^ However, this warrants future research using gene expressions mapping methods, such as eQTL mapping or chromatin interaction mapping.^140^

The overlapping genes across studies should be interpreted cautiously. One reason is that many studies only reported variants that passed the suggestively significant threshold but not the genome-wide significant *P* value. In addition, many studies lacked replication. Second, the heterogeneity of study designs exists in included studies, such as variability of participant characteristics (eg, age, ethnicity), the disease that led to pain (eg, osteoarthritis, diabetes), the nature of pain (eg, nociceptive, neuropathic, and nociplastic), differences in pain measurements (pain measured by pain questionnaire or ICD codes), and different genotyping platforms. Moreover, the overlapping findings on musculoskeletal pain phenotypes might be because of sample overlaps and should be further validated (see also paragraph 3.4.7).

Of the genes identified in candidate gene studies, only a few were replicated in GWASes. For instance, *COMT* and *OPRM1* are the 2 most investigated genes in various pain phenotypes in humans and mice. However, *COMT* was only reported once in all included GWAS articles, and *OPRM1* was not reported at all, which could be explained by the small effect size of these variants in the multifactorial pain phenotypes or insufficient statistical power of the candidate gene studies because of small sample size. Variants in these genes could still be identified for certain pain phenotypes when statistical power increases. On the other hand, these results also suggest that the choice of gene/pathway in the hypothesis-driven approach might be biased. Therefore, hypothesis-free methods are needed to uncover (novel) biological mechanisms of pain.

We excluded migraine and headache for this review, considering that central nervous system disorders might have different mechanisms compared with the peripheral types of pain. Surprisingly, 15 of 26 genes that overlap with the Human Pain Genetic Database are previously associated with migraine. This overlap might be explained by the phenotypic correlations between migraine and other types of pain, such as fibromyalgia,^104^ and the possible link with dysmenorrhoea pain.^78^ However, we also identified genes linked to migraine and phenotypes without a direct correlation with migraines, such as CIPN, opioid analgesia, and postoperative pain. These results indicate that investigation of the shared genetic background of pain might be worth pursuing, which can be done by cutting-edged methods, such as linkage disequilibrium score regression.

Genome-wide association study findings on pain will facilitate the understanding of pain development and clinical management of pain. To fully interpret how the noncoding variants identified by GWASes are involved in pain development, we need comprehensive biological annotation tools from different transcription levels, such as epigenetic regulation, noncoding RNA function, and gene expression profiles.^30^ Although fully understanding the functions of these variants might be challenging at this moment, it does not withhold the introduction of these variants in a clinical setting. One successful example is applying a polygenic risk score (PRS) based on GWAS results for breast cancer prediction.^60^ However, we are still far from the clinical application of genetic factors for pain development prediction and personalized pain management. Because this area is still under investigation, no unequivocal genetic predictors have been found yet.^48,91^

To the best of our knowledge, this review is the first systematic overview of GWASes on pain and related phenotypes in humans to date. Other strengths of this study are that we included articles reporting genetic association of pain-related phenotypes (such as neuropathy and pain treatment response), followed by the standards of PRISMA guidelines, and checked the reporting quality according to STREGA for genetic association studies. In addition, a systematic examination of the overlap between different studies was performed.

However, our review also has some limitations. Concerning article selection steps, we had to exclude 2 letters because no information on methods was provided to determine the reporting quality. However, these 2 articles might include important findings. One article reported the genetic associations between a variant in *TRPM8* and pain in Parkinson disease,^142^ and the other article reported an association between an intergenic variant rs3115229 and acute severe vaso-occlusive pain.^16^ In addition, articles investigating other markers than genetic markers (such as epigenetic markers) were excluded because this was not in line with our goal. Furthermore, the function of identified loci was not further annotated (eg, expression quantitative trait locus). Moreover, this review only focuses on GWAS findings, which might neglect important findings from candidate gene studies. To overcome this, we checked recent findings in candidate gene studies by comparing overlap between genes reported in GWASes and 2 comprehensive pain genetic databases.

Our study provides an overview of the identified and potential genetic risk factors for pain from GWAS findings, suggesting that multiple genetic risk factors involved in different functions can influence susceptibility to pain. For further studies, the overlapping genes (such as the 6 overlapping genes reported from GWASes, HPGDB, and the mouse pain genetics database) might be worth validation with careful experiment design, sufficient statistical power, and robust statistical methods to minimize incidental findings and yield validated results.^49^ Especially, genes implicated in neurological functions and inflammation might be prioritized for validation and further investigation. In addition, more efforts should be made to characterize the multiomics biomarker signatures of pain, such as gene expression, epigenetics, and metabolic profiles. Besides, to empower accurate replication, meta-analysis, and international collaborations, it is highly recommended that future studies use clear, consistent, phenotype definitions aligned with the current diagnosis definition/system, such as ICD-11 classification for chronic pain.^130^ A comprehensive understanding of the biological mechanisms of pain will finally benefit patients by improving the clinical management of pain.

## Conflict of interest statement

The authors have no relevant financial or nonfinancial interests to disclose. All authors' conflicts of interest in the manuscript is in agreement with the COI statement on the ICJME forms submitted in the submission system.

## Appendix A. Supplemental digital content

Supplemental digital content associated with this article can be found online at http://links.lww.com/PAIN/B808.

## Supplementary Material

## Acknowledgments

S. Li was supported by China Scholarship Council (CSC) Grant number 201908130179.
