# Supplementary material for: A systematic review of genome-wide association studies for pain, nociception, neuropathy, and pain treatment responses
Source: Pain. 2023 May 5;164(9):1891–911. doi: 10.1097/j.pain.0000000000002910 (PMC10436363; doi:10.1097/j.pain.0000000000002910)
Supplement: Supplementary file 1 [file jop-164-1891-s001.pdf]

Table S1. Search strategy.

**Search date**	2022-02-21
**PubMed/MEDLINE**	Element 1 (#1):<br>"Pain Perception"[Mesh] OR "Pain Threshold"[Mesh] OR "Pain Measurement"[Mesh] OR "Pain"[Mesh] OR Pain[tiab]<br>Element 2 (#2):<br>"Nociceptors"[Mesh] OR nocicept*[tiab]<br>Element 3 (#3):<br>"Neuralgia"[Mesh] OR "Peripheral Nervous System Diseases"[Mesh] OR Neuropathy[tiab]<br>Element 4 (#4):<br>"Genome-Wide Association Study"[Mesh] OR GWAS[tiab] OR GWA Stud*[tiab] OR Genome Wide Association Stud*[tiab] OR Genome Wide Association Analys*[tiab] OR Whole Genome Association Stud*[tiab] OR Whole Genome Association Analys*[tiab] OR Genome-wide scan*[tiab] OR Genome Wide Association Scan*[tiab] OR Whole Genome association scan*[tiab] OR genome wide association meta-analys*[tiab]<br>Search strategy:<br>(#1 OR #2 OR #3) AND #4 AND ("2000/01/01"[Date - Publication] : "2021/12/31"[Date - Publication])
**Embase**	#1: exp pain/ or exp pain threshold/ or pain intensity/ or pain assessment/ or pain measurement/ or pain receptor/ or (Pain or pains).ti,ab,kf.<br>#2: nociception/ or nocicept*.ti,ab,kf.<br>#3: neuropathic pain/ or neuropathy/<br>#4: (GWAS or GWA Stud* or Genome Wide Association Stud* or Genome Wide Association Analys* or Whole Genome Association Stud* or Whole Genome Association Analys* or Genome-wide scan* or Genome Wide Association Scan* or Whole Genome association scan* or genome wide association meta-analys*).ti,ab,kf. or genome-wide association study/<br>#5: (1 or 2 or 3) and 4<br>#6: limit 5 to (full text and human and English language and "remove preprint records" and yr="2000 - 2021")

Table S2. Inclusion and exclusion criteria for paper selection process.

Inclusion criteria:	Exclusion criteria:
Studies focusing on pain, nociception, or neuropathy.<br><br>Genome wide association studies (no other association studies).<br><br>Studies performed in humans.<br><br>Studies published in English and in peer-reviewed journals.	Wrong outcome<br><br>- Studies focusing on headache or migraine.<br>- Studies on the background of a disease or condition (such as osteoarthritis) rather than pain itself.<br><br>Wrong study design:<br><br>- Candidate gene study, not genome-wide, replication study, Mendelian randomization study, genetic correlation study, family study<br>- Studies investigating gene expression, metabolic, methylation profiles rather than genetic markers.<br><br>Non-human study: e.g., cell lines, animal, in silico studies<br><br>Wrong publication type:<br><br>- Review, case repot, commentary, abstract, study protocol, and letters.

Table S3. Quality assessment form according to the "STrengthening the REporting of Genetic Association studies" (STREGA) guidelines.

Item	Item number	STROBE Guideline	Extension for Genetic Association Studies (STREGA)	Score
**Title and Abstract**	1	(a) Indicate the study's design with a commonly used term in the title or the abstract.		1
		(b) Provide in the abstract an informative and balanced summary of what was done and what was found.		
**Introduction**				
Background rationale	2	Explain the scientific background and rationale for the investigation being reported.		1
Objectives	3	State specific objectives, including any pre-specified hypotheses.	***State if the study is the first report of a genetic association, a replication effort, or both.***	1
**Methods**				
Study design	4	Present key elements of study design early in the paper.		1
Setting	5	Describe the setting, locations and relevant dates, including periods of recruitment, exposure, follow-up, and data collection.		1

Item	Item number	STROBE Guideline	Extension for Genetic Association Studies (STREGA)	Score
Participants	6	(a) **Cohort study –** Give the eligibility criteria, and the sources and methods of selection of participants. Describe methods of follow-up.<br><br>**Case-control study –** Give the eligibility criteria, and the sources and methods of case ascertainment and control selection. Give the rationale for the choice of cases and controls.<br><br>**Cross-sectional study –** Give the eligibility criteria, and the sources and methods of selection of participants.	***Give information on the criteria and methods for selection of subsets of participants from a larger study, when relevant***.	1
		(b) **Cohort study –** For matched studies, give matching criteria and number of exposed and unexposed.<br><br>**Case-control study –** For matched studies, give matching criteria and the number of controls per case.		
Variables	7	*(a)* Clearly define all outcomes, exposures, predictors, potential confounders, and effect modifiers. Give diagnostic criteria, if applicable.	***(b) Clearly define genetic exposures (genetic variants) using a widely-used nomenclature system. Identify variables likely to be associated with population stratification (confounding by ethnic origin).***	1
Data sources measurement	8*	*(a)* For each variable of interest, give sources of data and details of methods of assessment (measurement). Describe comparability of assessment methods if there is more than one group.	***(b) Describe laboratory methods, including source and storage of DNA, genotyping methods and platforms (including the allele calling algorithm used, and its version), error rates and call rates. State the laboratory/centre***	1

Item	Item number	STROBE Guideline	Extension for Genetic Association Studies (STREGA)	Score
			where genotyping was done. Describe comparability of laboratory methods if there is more than one group. Specify whether genotypes were assigned using all of the data from the study simultaneously or in smaller batches.	
Bias	9	*(a)* Describe any efforts to address potential sources of bias.	***(b) For quantitative outcome variables, specify if any investigation of potential bias resulting from pharmacotherapy was undertaken. If relevant, describe the nature and magnitude of the potential bias, and explain what approach was used to deal with this.***	1
Study size	10	Explain how the study size was arrived at.	***State power calculation.***	1
Quantitative variables	11	Explain how quantitative variables were handled in the analyses. If applicable, describe which groupings were chosen, and why.	***If applicable, describe how effects of treatment were dealt with.***<br><br>***If applicable, describe how covariates measurement were dealt with.***	1
Statistical methods	12	(a) Describe all statistical methods, including those used to control for confounding.	***State software version used and options (or settings) chosen.***	1
		(b) Describe any methods used to examine subgroups and interactions.		

Item	Item number	STROBE Guideline	Extension for Genetic Association Studies (STREGA)	Score
		(c) Explain how missing data were addressed.<br><br>(d) **Cohort study –** If applicable, explain how loss to follow-up was addressed.<br><br>**Case-control study –** If applicable, explain how matching of cases and controls was addressed.<br><br>**Cross-sectional study –** If applicable, describe analytical methods taking account of sampling strategy.<br><br>(e) Describe any sensitivity analyses.		
			(f) State whether Hardy-Weinberg equilibrium was considered and, if so, how.	1
			(g) Describe any methods used for inferring genotypes or haplotypes.	1
			(h) Describe any methods used to assess or address population stratification.	1
			(i) Describe any methods used to address multiple comparisons or to control risk of false positive findings.	1

Item	Item number	STROBE Guideline	Extension for Genetic Association Studies (STREGA)	Score
			(j) Describe any methods used to address and correct for relatedness among subjects	1
**Results**				
Participants	13*	(a) Report the numbers of individuals at each stage of the study – e.g., numbers potentially eligible, examined for eligibility, confirmed eligible, included in the study, completing follow-up, and analysed.	***Report numbers of individuals in whom genotyping was attempted and numbers of individuals in whom genotyping was successful.***	1
		(b) Give reasons for non-participation at each stage.		
		(c) Consider use of a flow diagram.		
Descriptive data	14*	(a) Give characteristics of study participants (e.g., demographic, clinical, social) and information on exposures and potential confounders.	***Consider giving information by genotype.***	1
		(b) Indicate the number of participants with missing data for each variable of interest.		

Item	Item number	STROBE Guideline	Extension for Genetic Association Studies (STREGA)	Score
		(c) **Cohort study –** Summarize follow-up time, e.g., average and total amount.		
Outcome data	15 *	**Cohort study-**Report numbers of outcome events or summary measures over time.	***Report outcomes (phenotypes) for each genotype category over time***	1
		**Case-control study –** Report numbers in each exposure category, or summary measures of exposure.	***Report numbers in each genotype category***	
		**Cross-sectional study –** Report numbers of outcome events or summary measures.	***Report outcomes (phenotypes) for each genotype category***	
			Report effect size/odds ratio, allele frequency, confidence intervals and P-value	
Main results	16	(a) Give unadjusted estimates and, if applicable, confounder-adjusted estimates and their precision (e.g., 95% confidence intervals). Make clear which confounders were adjusted for and why they were included.		1

Item	Item number	STROBE Guideline	Extension for Genetic Association Studies (STREGA)	Score
		(b) Report category boundaries when continuous variables were categorized.		
		(c) If relevant, consider translating estimates of relative risk into absolute risk for a meaningful time period.		
			(d) Report results of any adjustments for multiple comparisons.	1
Other analyses	17	(a) Report other analyses done – e.g., analyses of subgroups and interactions, and sensitivity analyses.		1
			(b) If numerous genetic exposures (genetic variants) were examined, summarize results from all analyses undertaken.	1
			(c) If detailed results (e.g., summary statistics) are available elsewhere, state how they can be accessed.	1
**Discussion**				
Key results	18	Summarize key results with reference to study objectives.		1

Item	Item number	STROBE Guideline	Extension for Genetic Association Studies (STREGA)	Score
Limitations	19	Discuss limitations of the study, taking into account sources of potential bias or imprecision. Discuss both direction and magnitude of any potential bias.		1
Interpretation	20	Give a cautious overall interpretation of results considering objectives, limitations, multiplicity of analyses, results from similar studies, and other relevant evidence.		1
Generalizability	21	Discuss the generalizability (external validity) of the study results.		1
**Other Information**				
Funding	22	Give the source of funding and the role of the funders for the present study and, if applicable, for the original study on which the present article is based.		1

Table S4. Outcome phenotype definitions of included papers.

**Author, year**	**Phenotype definition**
Adjei, 2021	Chemotherapy-induced peripheral neuropathy (CIPN) was measured using the QLQ-CIPN20 questionnaire. The CIPN20 scores were re-scaled in both populations so that 0 represented the most severe symptoms and 100 represented no symptoms. Hence, a negative change from baseline in N08Cx cohort corresponded to worsening of symptoms, and a lower score corresponded to worse symptoms in both N08Cx and the MCBDR cohorts.
Baldwin, 2012	The adverse events were graded according to the National Cancer Institute Common Toxicity Criteria for Adverse Events (NCI-CTCAE) version 2.0. The analyses were carried out using 2 complementary endpoints: (i) the cumulative dose level triggering the first grade II or higher treatment related sensory peripheral neuropathy episode and (ii) the maximum observed treatment-related sensory peripheral neuropathy grade.
Campo, 2017	Adverse Events, defining the range of severity of neuropathy cases as grade 0–4. Comparisons were made between peripheral neuropathy (PN) (grade 2-4) and no or subclinical PN (grade 0-1).
Chua, 2020	Chemotherapy-induced peripheral neuropathy (CIPN) were graded according to the National Cancer Institute Common Terminology Criteria for Adverse Events, defining the range of severity of neuropathy cases as grade 0–4. Because the incidence of the toxicity is dependent on cumulative drug exposure, sensory peripheral neuropathy (PN) was assessed with a dose-to-event phenotype. A microtubule targeting agents (MTA)-induced sensory PN event was defined as the cumulative MTA dose (mg/m2) to first instance of grade 2 or higher sensory PN.
Cook-Sather, 2014	Children's Hospital of Eastern Ontario Pain Scale scores ranging from 4 to 13 were used, they were normalized to a 0–10 scale. Maximum and minimum postoperative pain scores (0–10), and postoperative and total (intraoperative plus postoperative) morphine in µg/kg were calculated.
Diouf, 2015	Children in the St Jude Total XIIIB study were graded according to NCI Common Terminology Criteria for Adverse Events (CTCAE) version 1.0 and those in the COG cohort according to a modified NCI CTCAE version 2.0. Neuropathy events were assessed as mild (grade 1), moderate (grade 2), serious/disabling (grade 3), or life threatening (grade 4). Those with grades 2, 3, or 4 motor and/or sensory neuropathy were considered neuropathy cases. There were no neuropathy-related deaths (grade 5).
Docampo, 2014	All patients fulfilled the 1990s American College of Rheumatology (ACR) criteria for fibromyalgia (FM) and were selected by the rheumatologists of the units participating in the study. Patients were then evaluated by another group of physicians trained in the assessment of FM patients. Diagnosis of FM was based on questionnaires and physical examination, and detailed descriptions of these information can be found in this paper [PMID: 24098674]. Three different control cohorts were used for this study: Gabriel consortium (ECHRS), National DNA Bank of Salamanca, and Spanish blood donor samples.

Dolan, 2017	The frequency of sensory neuropathy was evaluated using nine items in the EORTC-CIPN20. Four ordinal groups were derived reflecting the average severity across symptoms: none (0; mean = 0), a little (1; 0 < mean ≤ 1), quite a bit (2; 1 < mean ≤ 2), very much (3; 2 < mean ≤ 3). Groups 2 and 3 were combined due to low frequency.
Dunbar, 2020	Patterns of pain were defined using a 6-category severity–frequency classification system with O = no pain; A = episodes of mild pain; B = constant mild to moderate pain; C = episodes of severe pain; D = constant mild and episodes of severe pain; E = constant-severe pain. For this study, subjects responding with D or E were classified as constant-severe pain, while subjects responding with O, A, B, or C were classified as not constant- severe pain.
Fontanillas, 2021	The pain sensitivity questionnaire (PSQ) contains 14 questions in which participants should imagine themselves in certain situations. Participants should then grade how painful they would be, from 0 that stands for no pain to 10, the most severe pain that participants can imagine or consider possible. The total PSQ score is the mean of the 14 responses.<br><br>For the cold pressor test (CPT), participants were asked to prepare their own bath of ice water at home, and to keep their non-dominant hand submerged to the wrist for no more than 150 seconds. Two primary outcomes were assessed: cold pain threshold and cold pain tolerance. Cold pain threshold was the time to the first report of pain and cold tolerance the time to removal of the hand from the water.
Freidin, 2019	For the UK Biobank, cases of back pain (BP) were defined as those who reported “Back pain” in the response to the question: “Pain type(s) experienced in the past month.” Controls were defined as those who did not report BP in response to this question. Individuals who did not reply or replied: “Prefer not to answer” or “Pain all over the body” were excluded.<br><br>For the CHARGE Consortium, cases were defined as those reporting BP present for at least 3 months, whereas the controls were defined as those who reported no BP or BP with shorter duration. Thus, the definition of BP in these cohorts corresponded to chronic BP.
Freidin, 2021	For the UK Biobank: Those who indicated “Back pain” in response to the data-field 6159 (Pain types) question and also replied “Yes” to the data-field 3571 (Back pain for 3 months) question were classified as cases. Those who did not indicate “Back pain” in response to the data field 6159 or replied “No” to the data field 3571 question were classified as controls.<br><br>For the Generation Scotland (GS): The definition of chronic back pain (cBP) cases is those who selected BP option for more than 3 months, while the controls are all other participants.<br><br>For the orofacial Pain cohort: The phenotype of cBP was defined as participants that reported having more than 5 episodes of BP in the past year and those that reported between 2 to 4 episodes last year and that the episode lasted more than 2 hours. Participants reporting chronic widespread pain and fibromyalgia were excluded.

	For the HUNT study: The questionnaire data were used, with the participants who at least 3 consecutive months of pain and/or stiffness in muscles and listed lower BP or upper BP as complaint regions. Fibromyalgia participants were excluded both from cases and controls.<br><br>For ELSA cohort: Participants who positively responded to the questions “Whether often troubled with pain” and “Whether feel pain in back” were considered to have BP during a particular wave, whereas those who replied negatively to the first and/or second question were considered not to have BP. After obtaining these data in each wave separately, those who were cases in at least 2 waves were defined as cBP cases, whereas the rest were defined as controls.
Galvan, 2011	The pain relief phenotype under study is semi-quantitative and determined based on the BPI, a robust and psychometric validated method to assess the subjective severity of pain. Pain relief is measured using an 11-point numerical rating scale, from 0%, representing "no pain relief" to 100% or "complete pain relief," that is, 0%, 10%, 20%, etc.. For genome-wide association analysis, the cancer patients were first defined as "good" or "poor" responders to opioid therapy, based on their pain relief phenotype score of 90% or more or 40% or less, respectively.
García-Sanz, 2017	Patients who developed neuropathy (grade ≥ 2, NCI-CTCAE) were compared with those who did not.
Hertz, 2016	The primary endpoint for GWAS analysis was the cumulative docetaxel dose (mg/m2) at first report of treatment-related grade 3+ sensory peripheral neuropathy defined by National Cancer Institute Common Toxicity Criteria for Adverse Events version 3.0.
Hirata, 2018	Dysmenorrhea pain severity was originally queried in Japanese using a five-level word-association scale with 1 = not at all painful, 2 = not very painful, 3 = neither painful or unpainful, 4 = slightly painful, and 5 = very painful; (Closest English translations). Then, the integer values were transformed into an 11-point Numeric Rating Scale (NRS) which can be sub-divided into ranges with 0 = No pain, 1–3 = Mild Pain, 4–6 = Moderate Pain, and 7–10 = Severe Pain. Five-levels of pain severity were mapped to the NRS as 1-> 0 (No pain), 2-> 1 (Mild pain), 3-> 2 (Mild pain), 4-> 5 (Moderate pain), and 5-> 10 (Severe pain) for the genome-wide association analysis.
Janicki, 2016	Diagnosis of complex regional pain syndrome (CRPS) confirmed by motor/trophic changes that fulfill the International Association of the Study of Pain criteria for CRPS, duration of CRPS symptoms>1 year, and currently under follow-up treatment in the Pain Clinic at either PSHMC or Drexel. CRPS was diagnosed by pain specialists of the participating centers experienced in diagnosis and treatment of CRPS patients. Controls were subjects without CRPS.
Johnston, 2019	A genome-wide association was conducted for multisite chronic pain (MCP), which was defined as the sum of body sites (category ID 100048) at which chronic pain (at least 3 months duration) was recorded: 0 to 7 sites. Those who answered that they had chronic pain ‘all over the body’ were excluded from the GWAS as there is some evidence that this phenotype relating to widespread pain can be substantially different from more localised chronic pain and should not, therefore, be considered a logical extension of the multisite scale.

Johnston, 2021	Multisite Chronic Pain (MCP) was a quasi-quantitative variable defined as previously reported [PMID: 31194737]; briefly, this variable captures the number of body sites at which chronic pain (at least 3 months duration) was recorded: phenotypic values therefore ranged from 0 to 7. (excluding those with chronic widespread pain).
Jones, 2016	The dysmenorrhea pain severity phenotype was captured as an ordered variable with the following possible values scored as 0 , 1 , 2 , 3 ("not painful" . "little painful" . "moderately painful" . "extremely painful"), respectively, excluding responders selecting "I'm not sure."
Kanai, 2021	Patients with grade 0/1 peripheral sensory neuropathy (PSN) were compared to patients with grade 2/3 PSN.<br><br>For extreme phenotypes of PSN, patients who discontinued oxaliplatin early due to grade 2/3 PSN were selected and compared to those who maintained the status of grade 0 PSN after the completion of preplanned 6-month treatment without any dose reduction or delay of L-OHP.
Kim, 2009	Clinically induced pain was recorded with a paper and pencil form of a 100 mm visual analog scale (VAS). After the extraction of the impacted third molars, pain was recorded every 20 min by VAS until subjects requested analgesic medication as the local anesthesia was eliminated and post-operative pain onset occurred. The maximum post-operative pain rating, post-operative pain onset time and the analgesic onset time after ketorolac administration were used as measures of clinical pain and the onset of nonsteroidal anti-inflammatory drug (NSAID) analgesia.
Komatsu, 2015	Patients who developed ≥ grade 2 paclitaxel-induced sensory peripheral neuropathy (cases) were compared with patients who did not show neuropathy (controls). The grade of toxicity was classified in accordance with the US National Cancer Institute's Common Toxicity Criteria version 2.0.
Leandro-García, 2013	Neuropathy symptoms at baseline and cumulative paclitaxel dose at first neuropathy event, at grade 2 sensory neuropathy, and at maximum neuropathy grade were also collected from all patients. Patients with no or minimal adverse reaction (grade 0/1) were censored at total paclitaxel cumulative dose (mg). The grading was based on a common questionnaire modified on the National Cancer Institute Common Toxicity Criteria V.2.
Lee, 2019	A pain score was determined as the mean of four pain severity items (from 0 = no pain to 10 = the worst imaginable pain) as suggested by the Brief Pain Inventory developers, and moderate to severe pain (pain score ≥ 4) was considered clinically relevant. Therefore, cases were defined as those that had a pain score ≥ 4 at post-radiotherapy and the reference group included those with a pain score < 4 at post- radiotherapy.
Leger, 2014	Peripheral neuropathy was assessed at each study visit and was categorized as grade 1 (asymptomatic with sensory alteration on exam or minimal paresthesia causing no or minimal interference with usual social and functional activities), grade 2 (sensory alteration or paresthesia causing greater than minimal interference with usual social and functional activities), and grade 3 (sensory alteration or paresthesia causing inability to perform usual social and functional activities). Analyses were performed separately for grade ≥1, grade

	≥2, and grade 3 peripheral neuropathies. Controls were patients who did not develop such signs or symptoms within 96 weeks of initiating didanosine- or zidovudine/lamivudine-containing regimens/Stavudine.
Lemmela, 2016	In the YFS study, information on physician-diagnosed sciatica was inquired during on-site examinations using a self-administered questionnaire ("Do you currently have or have you had a long-term disease diagnosed by a physician, such as sciatica?"). In the H2000, the diagnosis of sciatica was based on the presence of chronic (>3 months) low back pain radiating down to the leg and either clinical findings of lumbar nerve root compression or a history of lumbar disc herniation that had been previously verified by imaging or required surgery.<br><br>For the replication study (The FINRISK Study), those diagnosed with one of the ICD-codes selected a priori by two experts on musculoskeletal diseases (EVJ and MH) as relevant for sciatica or sciatic syndrome (ICD8 353, 728.8; ICD9 724.3, 722.1, 722.10, 722.5, 722.52, 355.0; ICD10 M54.3, M51.1, M54.1, M54.4) were included as cases.
Li, 2017	A visual analogue scale has previously been used to rate menstrual pain. In this study, a horizontal 10-cm visual analogue scale with endpoints were adopted, spanning from 'no pain at all' (score=0) on the far left to 'the worst pain' (score=10) on the far right. Scores of less than 1 were assigned to a control group, and scores higher than 4 (moderate intensity) were assigned to the case group. The participants with possible causes of secondary dysmenorrhoea, such as endometriosis and other gynecological problems, were excluded.
Li, 2019	POG studies 9904 and 9905. Based on National Cancer Institute Common Toxicity Criteria for Adverse Events version 2.0, the vincristine induced peripheral neuropathy (VIPN) events are defined when patients experienced symptomatic neurotoxicity with neuropathy grade ≥ 3 in either motor or sensory neurons.<br><br>ADVANCE trial. A 5-item total neuropathy score (TNS-PV) was used as phenotype to summarize the VIPN in this genetic association data analysis. The 5-item score includes sensory symptoms (i.e., numbness, tingling, and neuropathic pain), temperature sensibility, vibration sensibility, strength, and tendon reflexes.
Magrangeas, 2016	Adverse events including peripheral neuropathy were graded by NCI Common Toxicity Criteria Version 3.0. Grade ≥ 2 bortezomib-induced peripheral neuropathy (BiPN) patients were compared with control patients defined as grade 1 or no BiPN .
Meng, 2015 #1	A neuropathic pain case was defined in this study as a type 2 diabetic individual with a history of at least one prescription of any of the following five medicines, which are effective and recommended in diabetic peripheral neuropathy (Attal et al., 2010; Finnerup et al., 2010; NICE, 2013) and used less frequently for other indications: duloxetine, gabapentin, pregabalin, capsaicin cream/patch and lidocaine patch. The cases also had positive monofilament tests in at least one foot, indicating the likely presence of sensory neuropathy.<br><br>A control was defined as a type 2 diabetic individual with no prescription history of these five drugs, nor of the following 16 opioid analgesics (buprenorphine, codeine phosphate, diamorphine, dihydrocodeine, dipipanone, fentanyl, hydromorphone, meptazinol,

	methadone, morphine, oxycodone, papaveretum, pentazocine, pethidine, tapentadol and tramadol). Individuals with a prescription history of amitriptyline, carbamazepine or nortriptyline were excluded from controls since these are also frequently used to treat other disorders (although these drugs are effective in neuropathic pain).
Meng, 2015 #2	A neuropathic pain case was defined as a type 2 diabetic patient who has a history of multiple usages (minimum twice) of at least one of the following five medicines which are recommended and effective in diabetic peripheral neuropathy and prescribed uncommonly for other disorders: duloxetine, gabapentin, pregabalin, capsaicin cream (or patch) and lidocaine patch (Attal et al., 2010; National Institute for Health & Care Excellence NICE (UK), 2013; Finnerup et al., 2010).<br><br>A control was defined as a type 2 diabetic patient who has not been prescribed any of these five drugs before. Individuals who had a prescription history of amitriptyline, carbamazepine, or nortriptyline were not included as controls because these drugs are often used for the treatment of other medical conditions, as well as neuropathic pain. Individuals with a history of only one single prescription for any of these five drugs were excluded from both cases and controls.
Meng, 2019	For the UK Biobank: The knee pain cases were those who selected the 'knee pain' option for the UK Biobank Questionnaire field ID 6159, regardless of whether they had selected other options. The controls in this study were those who selected the 'None of the above' option.<br><br>Independent cohort 1—23andMe, Inc: Cases were defined as those self-reported having been diagnosed or treated for osteoarthritis. Controls were defined as those self-reporting as having not been diagnosed or treated for osteoarthritis.<br><br>Independent cohorts 2—OAI and JoCo: Cases were those with definitive knee osteoarthritis, defined as radiographic evidence of the presence of definite osteophytes and possible joint space narrowing (Kellgren-Lawrence grade ≥ 2) or total joint replacement in one or both knees. Controls were those having no or doubtful evidence of OA (Kellgren-Lawrence grade = 0 or 1) in both knees at all available time points.
Meng, 2020	For the UK Biobank: Cases were defined as participants who reported having activity limiting pain in the neck or shoulder in the past month (the UK Biobank Questionnaire field ID 6159), regardless of whether they reported pain in other regions. The controls were defined as participants who chose the 'None of the above' option.<br><br>Replication cohort 1—GS:SFHS: Participants were asked a few question, if a participant selected both 'Neck or shoulder pain' and 'Have you had this pain or discomfort for more than 3 months?', then he/she was defined as a case. All other subjects were defined as controls.<br><br>Replication cohort 2: TwinsUK: participants were asked 'In the past three months, have you had pain in your neck or shoulders?' Those who answered 'Yes' were defined as cases. Those who answered 'No' were defined as controls. Those with missing answers were not included in the study.

Mieda, 2016	24-h patient-controlled analgesia (PCA) fentanyl consumption, defined as the cumulative doses of fentanyl that were actually administered to the patients via the PCA pump during the first 24-h postoperative period, was used as the primary endpoint among various quantitative phenotypic traits.
Nishizawa, 2014	Requirements for an opioid analgesic as a continuous variable, fentanyl (μg kg-1), during the 24-h postoperative period.
Nishizawa, 2018	The average remifentanil infusion rate (in μg/kg/min) during surgery was calculated by dividing the total dose of remifentanil that was required during surgery by the duration of surgery and body weight. Prior to the analyses, the quantitative values of the average remifentanil infusion rate (μg/kg/min) were natural-log-transformed for approximation to the normal distribution according to the following formula: value for analyses = Ln (1 + average remifentanil infusion rate [μg/kg/min]).
Nishizawa, 2021	Chronic pain cases include: postherpetic neuralgia (PHN), lower back pain (LBP), hernia of intervertebral disk, spinal canal stenosis, postoperative pain, neck pain, others. 282 healthy adult volunteers were enrolled as controls who were disease-free, did not experience chronic pain, and who lived in or near the Kanto area in Japan.
Peters, 2012	Chronic widespread pain (CWP) was defined as subjects having pain in the left side of the body, in the right side of the body, above the waist, below the waist, and in the axial skeleton (following the Fibromyalgia Criteria of the American College of Rheumatology). Controls were defined as subjects not having CWP. Subjects using analgesics (ATC code: N0231) were excluded from the control group.
Rahman, 2021	In UKB, chronic widespread pain (CWP) cases were defined by combining self-reported diagnosis of pain all over the body lasting for >3 months; simultaneous pain in the knee, shoulder, hip and back lasting 3+ months and fibromyalgia. Controls comprised those who reported no pain in the last month or reported pain all over the body in the previous month that did not last for 3 months or reported only ≥3 months of non-musculoskeletal pain (headache, facial and abdominal pain). Those reporting a self-reported diagnosis of rheumatoid arthritis, polymyalgia rheumatica, arthritis not otherwise specified, systemic lupus erythematosus, ankylosing spondylitis and myopathy were excluded from the study.
Reyes-Gibby, 2016	Pain "during the past week" was rated using a standardized 11-point numeric scale (0 = "no pain" and 10 = "pain as bad as you can imagine"). A binary pain phenotype was adopted, where cases were individuals with severe pre-treatment pain (score ≥ 7) and controls were individuals with non-severe pre-treatment pain (score < 7), based on the National Comprehensive Cancer Network cutoff score for severe pain.
Reyes-Gibby, 2018	The following ICD-9 codes (053.13; 337.0; 337.09; 337.1; 356.4; 356.8; 356.9; 357.2; 357.3; 357.9; 377.41) and ICD-10 codes (G58.8; G58.9; G62.0; G62.2; G62.9; G63.0) were used to identify neuropathy in head and neck cancer (HNSCC) patient's cohort. HNSCC patients without these ICD codes were defined as controls.
Sanders , 2017	The HCHS/SOL cohort: To be classified as temporomandibular disorder (TMD) case, participants had to report having had pain in both their face and in their jaw joint.

	OPPERA cohort: Examiners determined classification of TMD cases who met all 3 of the following criteria: 1) pain reported with sufficient frequency in the cheeks, jaw muscles, temples, or jaw joints during the preceding 6 months; 2) pain reported in the examiner-defined orofacial region for at least 5 days out of the prior 30 days; and 3) pain reported in at least 3 masticatory muscles or at least 1 temporomandibular joint in response to palpation of the orofacial muscles or maneuver of the jaw.<br><br>SHIP cohort: Participants reported symptoms by questionnaire regarding pain in the temporomandibular joint and facial muscles; presence and frequency of pain were assessed. During a clinical exam, the examiner inquired about pain or discomfort upon palpation of masticatory tissues, including temporomandibular joints (dorsocranial and lateral) at 2 kg/cm2 and masseter, temporalis, and medial pterygoid at 1 kg/cm2.<br><br>NFBC: Participants (52% female) reported symptoms by responding to a questionnaire with the following questions: 1) “Do you experience temple, temporomandibular joint, face, or jaw pain once a week or more often?” 2) “Do you experience pain once a week or more often while opening your mouth wide?” A clinical exam determined the presence of examiner-evoked pain in 3 or more temporomandibular muscles and/or joints.<br><br>Brazilian Cohort: Pain history was determined by asking participants the following question: “Have you had pain in your head, face, jaw, or in front of the ears in the last 30 days?” The examiner manually palpated lateral and posterior temporomandibular joints (0.45 kg) and asked participants to report yes or no responses to the presence of pain.<br><br>Subjects did not meet those criteria in the cohort were defined as controls.
Schneider, 2015	Cases. Cases were defined as those experiencing grade 2–4 taxane-induced peripheral neuropathy (TIPN) as assessed by the Common Toxicity Criteria Adverse Events (CTCAE) version 3.0. Cases included patients who received at least one dose of paclitaxel and the neuropathy event occurred during treatment or within 3 months of the last dose of therapy.<br><br>Controls. Controls included patients who met all the following: (i) received all planned doses of paclitaxel; (ii) had follow-up for at least 3 months after the last dose of drug; (iii) did not meet any of the case definitions as outlined above; and (iv) had either paclitaxel or bevacizumab held or modified for any reason (i.e., disease progression or other toxicity) were excluded.
Smith, 2019	Temporomandibular disorder (TMD) cases met all 3 of the following criteria: (1) pain in the cheeks, jaw muscles, temples, or jaw joints that occurred for at least 5 days per month during the preceding 6 months, including at least 15 days in the month before enrollment; (2) pain reported in the examiner-defined orofacial region for at least 5 days out of the prior 30 days; and (c) pain evoked by palpation of the orofacial muscles or maneuver of the jaw that occurred in at least 3 masticatory muscles or at least 1 temporomandibular joint or both. Subjects did not meet those criteria in the cohort were defined as controls.
Sucheston-Campbell, 2018	Taxane-induced peripheral neuropathy were monitored and reported using the CTCAE, version 3.0, which contains descriptive terminology to be used for adverse event reporting. Grade 3 toxicities interfere with activities of daily living, and grade 4 adverse events are life-threatening and often require hospitalization. Logistic regression was performed between <grade 3 and >=grade 3.

Suri, 2018	Chronic back pain (CBP) cases were defined in this study using one of 3 definitions depending on the cohort: 1) ≥ 3 months of back pain, 2) ≥ 6 months of back pain, and 3) ≥ 1 month of back pain in consecutive years (reflecting ≥ 12 months of back pain). For each cohort, the comparison group ("controls") was comprised of those who reported not having back pain or reported back pain of insufficient duration to be included as a case.
Suri, 2021	For the low back pain requiring healthcare utilization (LBP-HC) phenotype: cases were defined as adults with 2 or more ICD-9 or ICD-10 codes indicating a phenotype, and controls were defined as adults with no codes indicating a phenotype (all codes included, available at http://links.lww.com/PAIN/B304). Adults with only 1 diagnostic code indicating a phenotype were omitted from the analysis (i.e., not included as cases or controls).
Takahashi, 2018	The baseline latency to pain perception, defined as the time of immersion of the hand in the ice water, before the i.v. injection of fentanyl (PPLpre) was recorded. A cut-off point was set at 150s. The hand was warmed with a hair dryer as soon as it was withdrawn from the ice water until the sensation of cold was completely abolished, then Fentanyl, 2 mg/kg was injected i.v. Three minutes after the injection, the pain perception latency of the dominant hand (PPLpost) was measured again. The analgesic effect of fentanyl in the preoperative cold pressor-induced pain test was evaluated simply as the difference between PPLpost and PPLpre (PPLpost - PPLpre).
Tang, 2019	The ACCORD and BARI 2D trials both defined neuropathy based on a Michigan Neuropathy Screening Instrument (MNSI) clinical examination that includes a focused examination of the feet to assess skin and structural abnormalities, along with assessment of distal vibration perception with a 128-Hz tuning fork and ankle reflexes. Diabetic peripheral neuropathy (DPN) case subjects were defined as participants having an MNSI > 2.0 at study entry and/or at any time during follow-up, whereas DPN control subjects were defined as participants having an MNSI < 2.0 at study entry and for the entire duration of follow-up.
Tsepilov, 2020	For the UK Biobank, those who reported back, neck or shoulder, hip, or knee pain lasting more than 3 months were considered chronic back, neck/shoulder, hip, and knee pain cases, respectively. Participants reporting no such pain lasting longer than 3 months were considered controls (regardless of whether they had another regional chronic pain, such as abdominal pain, or not). Individuals who preferred not to answer, reported more than 3 months of pain all over the body were excluded from the study.<br><br>To obtain genetic components explaining four chronic musculoskeletal pain phenotypes (chronic back, neck/shoulder, hip, and knee pain), a modified principal component analysis (PCA) technique was used to combine multiple correlated variables into a set of uncorrelated principal components (PCs). PCs are linear combinations of variables constructed such that the first PC explains the maximum proportion of the total variance of the set of traits, the second PC accounts for the largest proportion of the remaining variance, and so on.
van Reij, 2020	The primary outcome measured in this cohort was the highest surgery-related pain score at rest during the last week at 3 months after surgery measured by the numeric rating scale (NRS). Based on the primary outcome measure, patients were divided into a non-pain (NRS = 0) and a chronic postoperative pain (NRS > 3) group to perform an extreme phenotype analysis to increase the power. Patients with mild pain (NRS between 1 and 3) score were not included in the genetic analysis.

Veluchamy, 2021	GoDARTS and GS:SFHS:<br><br>Individuals with of possible Neuropathic pain (NP) (ie, case participants) were identified based on current reported pain and/or currently taking pain medications, pain duration of at least 3 months, and Douleur Neuropathique en 4 Questions (DN4) score greater than or equal to 3 of 7. Control participants were defined as those reporting no pain or not taking any pain medications at the time of completing the questionnaire. Participants who reported pain of less than 3 months' duration or who scored less than 3 on the DN4 were excluded.<br><br>For the UK Biobank: Self-reported prescribed medication linked to routine hospital admissions records were used as a proxy phenotype for NP. Briefly, case participants were defined as individuals with a record of the most commonly prescribed anti-neuropathic medicines, based on the NeuPSIG guidelines (ie, gabapentin, pregabalin, duloxetine). Control participants were those with no such reported prescriptions. Individuals reporting receipt of amitriptyline, other tricyclic antidepressants, and/or tramadol were excluded from the control and case groups, despite the potential role of these medicines in treating NP because of their frequent use to treat other conditions and consequent nonspecificity for NP. Individuals who self-reported an epilepsy diagnosis and/or any anti-epileptic medication concomitantly with a gabapentinoid alone were excluded.
Warner, 2017	Individuals were assigned a phenotype by classifying them according to their scores on the painDETECT questionnaire. This is a seven-item questionnaire scored from 0 to 39 that uses a Likert scale for participants to describe the nature of their pain, in order to distinguish it from nociceptive pain. Questions are included on qualities such as burning pain, tingling, sudden pain and sensitivity to heat and cold. In all cohorts, scores of > 12 were classified as 'possible neuropathic pain' according to the validated cut-offs for diagnosis by Freynhagen *et al* [PMID: 17022849]. All others in the cohort were defined as controls.
Winsvold, 2021	Identical criteria were used to define cases and controls in the HUNT and UK Biobank studies. Cases were defined by 1) the presence of at least one hospital contact with a registered diagnosis of idiopathic progressive neuropathy (ICD-10 G60.3, ICD-9 356.4), other specified idiopathic peripheral neuropathy (ICD-9 356.8), unspecified hereditary and idiopathic neuropathy (ICD-10 G60.9), or unspecified polyneuropathy (ICD-10 G62.9, ICD-9 356.9); 2) no hospital contact with a registered diagnosis of diabetes (ICD-10 E10–E14, ICD-9 250).<br><br>Controls included all participants who had no hospital contacts with a registered diagnosis of hereditary or idiopathic polyneuropathy (ICD-10 G60, ICD-9 356), other inflammatory polyneuropathy (ICD-10 G61.8, ICD-9 357), unspecified inflammatory polyneuropathy (ICD-10 G61.9), other and unspecified polyneuropathies (ICD-10 G62), polyneuropathy in diseases classified elsewhere (ICD-10 G63), idiopathic peripheral autonomic neuropathy (ICD-10 G90.0), paraneoplastic neuropathy (ICD-10 G13.0), autonomic neuropathy in diseases classified elsewhere (ICD-10 G99.0), or diabetes (ICD-10 E10–E14, ICD-9 250).
Won, 2012	Neuropathy was evaluated and rated in accordance with National Cancer Institute (NCI) criteria. Cases were defined as prolonged (≥7 days) grade 2 or grade 3 events. All the others in the cohort were defined as controls.

Yokoshima, 2018	Opioid analgesic responsiveness were evaluated by pain intensity (NRS) on a 5-point Likert scale (responses were scored as 0 = absence of symptoms, 1 = mild, 2 = moderate, 3 = severe, and 4 = very severe) before and after prescribing firstly or increasing opioid analgesics. Opioid analgesic responsiveness was defined as pain decrease corresponding to increased opioid analgesics.

Table S5. List of excluded studies after full-text assessment.

Author, Year	Title	PubMed ID	Reason for exclusion
Bjornsdottir, 2019	A PRPH splice-donor variant associates with reduced sural nerve amplitude and risk of peripheral neuropathy	30992453	Outcome: nerve conduction
Bjornsdottir, 2017	Sequence variant at 8q24.21 associates with sciatica caused by lumbar disc herniation	28223688	Outcome: lumbar disc herniation
Chaturvedi, 2017	Genome-wide association study to identify variants associated with acute severe vaso-occlusive pain in sickle cell anemia	28584135	Publication type: letter
Cox, 2020	Genome-wide association study of opioid cessation	31936517	Outcome: opioid cessation
Freidin, 2021	An association between chronic widespread pain and the gut microbiome	33331911	Study design: not genetic marker of humans
Johnston, 2019	Identification of novel common variants associated with chronic pain using conditional false discovery rate analysis with major depressive disorder and assessment of pleiotropic effects of LRFN5	31748543	Study design: analysis using published GWAS summary statistics
Mahmoudpour, 2018	Chemotherapy-induced peripheral neuropathy: evidence from genome-wide association studies and replication within multiple myeloma patients	30111286	Study design: replication study
Meng, 2017	A genome-wide association study suggests that MAPK14 is associated with diabetic foot ulcers	28672053	Outcome: diabetic foot ulcer
Ruau, 2012	Integrative approach to pain genetics identifies pain sensitivity loci across diseases	22685391	Study design: candidate gene study not GWAS
Sanchez-Roige, 2021	Genome-wide association study of problematic opioid prescription use in 132,113 23andMe research participants of European ancestry	34728798	Outcome: opioid dependence/cession
Smith, 2017	Genome-wide association study of therapeutic opioid dosing identifies a novel locus upstream of OPRM1	28115739	Outcome: opioid dependence/cessation
Trendowski, 2020	Clinical and genome-wide analysis of multiple severe cisplatin-induced neurotoxicities in adult-onset cancer survivors	32998964	Study design: hearing loss was included for cases
Ustinova, 2021	Novel susceptibility loci identified in a genome-wide association study of type 2 diabetes complications in population of Latvia	33430853	Study design: peripheral circulatory complications were included for cases

Wheeler, 2013	Integration of cell line and clinical trial genome-wide analyses supports a polygenic architecture of Paclitaxel-induced sensory peripheral neuropathy	23204130	Study design: analysis using previous GWAS summary statistics
Williams, 2013	Novel genetic variants associated with lumbar disc degeneration in northern Europeans: a meta-analysis of 4600 subjects	22993228	Outcome: lumbar disc degeneration
Williams, 2020	Genome-wide association study of pain in Parkinson's disease implicates TRPM8 as a risk factor	32078185	Publication type: letter
Backman, 2021*	Exome sequencing and analysis of 454,787 UK Biobank participants.	34662886	Study design: not a focus on pain related phenotype
Jiang, 2021*	A generalized linear mixed model association tool for biobank-scale data.	34737426	Study design: not a focus on pain related phenotype
Dönertaş, 2021*	Common genetic associations between age-related diseases.	33959723	Study design: not a focus on pain related phenotype
Sakaue, 2021*	A cross-population atlas of genetic associations for 220 human phenotypes.	34594039	Study design: not a focus on pain related phenotype
Watts, 2021*	Genome-wide association studies of toxicity to oxaliplatin and fluoropyrimidine chemotherapy with or without cetuximab in 1800 patients with advanced colorectal cancer.	34270794	Outcome: No results on neuropathy were reported

* Asterisk indicates additional papers found by checking the GWAS catalog.

Table S6. Quality assessment score of included papers according to STREGA guidelines.

**Study**	**Title & Abstract (Max. score =1)**	**Introduction (Max. score =2)**	**Method (Max. score =14)**	**Results (Max. score =8)**	**Discussion (Max. score =4)**	**Funding (Max. score =1)**	**Quality score (Max. score =30)**
Adjei, 2021	1	2	11	7	4	1	26
Baldwin, 2012	1	2	10	5	4	1	23
Campo, 2017	1	2	10	5	2	1	21
Chua, 2020	1	2	12	7	3	1	26
Cook-Sather, 2014	1	2	12	4	4	1	24
Diouf, 2015	1	2	10	7	3	1	24
Docampo, 2014	1	2	9	4	4	0	20
Dolan, 2017	1	2	12	6	3	1	25
Dunbar, 2020	1	2	10	6	3	1	23
Fontanillas, 2021	1	2	11	6	3	1	24
Freidin, 2019	1	2	11	7	4	1	26
Freidin, 2021	1	2	11	7	4	1	26
Galvan, 2011	1	2	8	5	2	1	19
García-Sanz, 2017	1	2	8	4	2	1	18
Hertz, 2016	1	2	11	6	4	1	25
Hirata, 2018	1	2	11	6	2	0	22
Janicki, 2016	1	2	10	4	3	1	21
Johnston, 2019	1	2	13	8	3	1	28

Johnston, 2021	1	2	11	6	3	1	24
Jones, 2016	1	2	11	6	3	1	24
Kanai , 2021	1	2	11	6	2	1	23
Kim, 2009	1	2	10	3	3	0	19
Komatsu, 2015	1	2	6	4	2	1	16
Leandro-García, 2013	1	2	11	6	2	1	23
Lee, 2019	1	2	11	7	3	1	25
Leger, 2014	1	2	7	5	3	1	19
Lemmela, 2016	1	2	11	7	3	1	25
Li, 2017	1	2	11	6	4	1	25
Li, 2019	1	2	11	6	4	1	25
Magrangeas, 2016	1	2	10	5	3	1	22
Meng, 2015 #1	1	2	12	4	3	1	23
Meng, 2015 #2	1	2	13	5	3	1	25
Meng, 2019	1	2	11	5	4	1	24
Meng, 2020	1	2	11	6	4	1	25
Mieda, 2016	1	2	8	6	2	1	20
Nishizawa, 2014	1	2	8	6	2	1	20
Nishizawa, 2018	1	2	8	6	3	1	21
Nishizawa, 2021	1	2	7	4	3	1	18
Peters, 2012	1	2	12	6	4	1	26

Rahman, 2021	1	1	14	8	4	1	29
Reyes-Gibby, 2016	1	2	12	5	3	1	24
Reyes-Gibby, 2018	1	2	12	7	3	1	26
Sanders, 2017	1	2	12	5	4	1	25
Schneider , 2015	1	2	11	5	4	1	24
Smith, 2019	1	2	13	7	4	1	28
Sucheston-Campbell, 2018	1	2	11	6	3	1	24
Suri, 2018	1	2	12	7	4	1	27
Suri, 2021	1	2	13	7	4	1	28
Takahashi, 2018	1	2	10	6	2	1	22
Tang, 2019	1	2	12	7	4	1	27
Tsepilov, 2020	1	2	13	7	4	1	28
van Reij, 2020	1	2	14	7	4	1	29
Veluchamy, 2021	1	2	13	5	4	1	26
Warner, 2017	1	2	13	7	4	1	28
Winsvold, 2021	1	2	14	5	4	1	27
Won, 2012	1	2	7	6	3	1	20
Yokoshima, 2018	1	2	7	5	3	1	19

Table S7. Replication and meta-analysis information of included papers.

	Replication study					Joint meta-analysis			
Author, Year	Replication analysis type	Sample size	Ethnicity	P-value threshold	No. of replicated locus in replication	Available	P-value threshold	No. of replicated locus in joint meta-analysis	Comments
Adjei, 2021	Single independent replication cohort	381	NS	1.00E-06	4	No	NS	NS	The SNPs found in replication were different independent loci from discovery
Baldwin, 2012	Replication was split from the discovery cohort	271	EA; AA	NS	NS	No	NS	NS	This study is CALGB 40101, which is used for many following studies as replication cohort.
Campo, 2017	No replication	NS	NS	NS	NS	No	NS	NS	
Chua, 2020	Single independent replication cohort from previous published results: CALGB 40101	855	EA	NS	NS	Yes	1.00E-05	15	
Cook-Sather, 2014	Replication was split from the discovery cohort	145	EA; AA	NS	NS	No	NS	NS	Replicaiton was only conducted for total morphine dose phenotype
Diouf, 2015	Single independent replication cohort	99	EA; AA; Asian; Hispanic; other	NS	NS	Yes	5.00E-08	1	
Docampo, 2014	Replication was split from the discovery cohort	1532	White Spanish	2.94E-03	0	Yes	NS	NS	Three suggestive significant signals in joint meta-analysis were

									added to describe in the results section
Dolan, 2017	No replication	NS	NS	NS	NS	No	NS	NS	
Dunbar, 2020	No replication	NS	NS	NS	NS	No	NS	NS	
Fontanillas, 2021	Two phenotype cohorts replicated in each other	25321 for CPT phenotype replication, 6853 for PSQ phenotype replication	EA	NS	NS	No	NS	NS	
Freidin, 2019	Meta-analysis of several independent cohorts	154970-157752	EA; AA; SA; Chinese	1.00E-02	3	Yes	5.00E-08	1	Meta-analysis in EA only
Freidin, 2021	Meta-analysis of several independent cohorts	43740 males; 50092 females	EA	5.60E-03	2	No	NS	NS	Discovery study is sex-stratified analysis
Galvan, 2011	Replication was split from the discovery cohort	570	EA	NS	NS	Yes	5.00E-08	1	
García-Sanz, 2017	No replication	NS	NS	NS	NS	No	NS	NS	
Hertz, 2016	Single independent replication cohort from previous published results: CALGB 40101	855	EA	5.00E-02	0	No	NS	NS	
Hirata, 2018	No replication	NS	NS	NS	NS	No	NS	NS	
Janicki, 2016	Replication was split from the discovery cohort	230	EA;AA;Hispanics	2.00E-03	0	Yes	2.00E-03	0	One strong signal in joint meta-analysis was added to describe in the results section

Johnston, 2019	No replication	NS	NS	NS	NS	No	NS	NS	
Johnston, 2021	No replication	NS	NS	NS	NS	No	NS	NS	Discovery study is sex-stratified analysis
Jones, 2016	No replication	NS	NS	NS	NS	No	NS	NS	
Kanai , 2021	No replication	NS	NS	NS	NS	No	NS	NS	
Kim, 2009	No replication	NS	NS	NS	NS	No	NS	NS	
Komatsu, 2015	No replication	NS	NS	NS	NS	No	NS	NS	Discovery GWAS results were not reported
Leandro-García, 2013	No replication	NS	NS	NS	NS	No	NS	NS	
Lee, 2019	No replication	NS	NS	NS	NS	No	NS	NS	
Leger, 2014	No replication	NS	NS	NS	NS	No	NS	NS	
Lemmela, 2016	Single independent replication cohort	19265	Finnish	5.00E-02	1	No	NS	NS	
Li, 2017	Single independent replication cohort from previous published results: CALGB 40101	1446	Chinese	NS	NS	Yes	5.00E-08	2	
Li, 2019	Single independent replication cohort from previous published results: CALGB 40101	63	EA	NS	NS	No	NS	NS	
Magrangeas, 2016	Single independent replication cohort	114	NS	NS	NS	No	NS	NS	
Meng, 2015 #1	No replication	NS	NS	NS	NS	No	NS	NS	

Meng, 2015 #2	No replication	NS	NS	NS	NS	No	NS	NS	This study included both sex-unstratified and sex-stratified GWASes; The genome-wide significant locus found in sex-unstratified analysis was overlapped with one locus found in females
Meng, 2019	Individual analysis of several replication cohorts	23andMe: 1540125; OAI and JoCo: 4448	EA	NS	NS	No	NS	NS	
Meng, 2020	Individual analysis of several replication cohorts	GS:SHFS: 19598; TwinsUK: 3982	EA	5.00E-02	2	Yes	NS	NS	Two loci identified in discovery study were replicated only in one of the replication cohort
Mieda, 2016	Three-stage analysis: one cohort was divided into 3 sub-cohorts	NS	NS	NS	NS	No	NS	NS	
Nishizawa, 2014	Three-stage analysis: one cohort was divided into 3 sub-cohorts	NS	NS	NS	NS	No	NS	NS	
Nishizawa, 2018	Three-stage analysis: one cohort was divided into 3 sub-cohorts	NS	NS	NS	NS	No	NS	NS	
Nishizawa, 2021	No replication	NS	NS	NS	NS	No	NS	NS	
Peters, 2012	Meta-analysis of several independent cohorts	9469	EA	NS	NS	Yes	NS	NS	
Rahman, 2021	Meta-analysis of several independent cohorts	57257	EA	1.70E-02	1	No	NS	NS	

Reyes-Gibby, 2016	Replication was split from the discovery cohort	410	EA	NS	NS	Yes	5.00E-08	1	
Reyes-Gibby, 2018	No replication	NS	NS	NS	NS	No	NS	NS	
Sanders, 2017	Meta-analysis of several independent cohorts	8814	Multiple cohorts: OPPERA; SHIP; NFBC; Brazilian Cohort	5.00E-02	1	No	NS	NS	Two suggestive significant signals identified in the discovery analysis were added to describe in the results section
Schneider , 2015	Single independent replication cohort	925	EA; AA; other	1.70E-03	1	No	NS	NS	
Smith, 2019	Meta-analysis of several independent cohorts	157164	Multiple cohorts: SHIP; NFBC; SPB; OP2; CPPC; HCHS;SOL; UKB	1.70E-02	0	No	NS	NS	This study included both sex-unstratified and sex-stratified GWASes; The genome-wide significant locus found in sex-unstratified analysis was overlapped with one locus found in females
Sucheston-Campbell, 2018	Single independent replication cohort from previous published results: CALGB 40101	855	EA	NS	NS	Yes	1.00E-05	3	
Suri, 2018	Meta-analysis of several independent cohorts	283752	EA	1.25E-02	3	Yes	5.00E-08	3	
Suri, 2021	No replication	NS	NS	NS	NS	No	NS	NS	
Takahashi, 2018	Three-stage analysis: one cohort was divided into 3 sub-cohorts	NS	NS	NS	NS	No	NS	NS	

Tang, 2019	Single independent replication cohort from previous published results: CALGB 40101	949	EA	4.00E-03	1	Yes	5.00E-08	1	
Tsepilov, 2020	Replication was split from the discovery cohort	191580	EA; AA; SA	5.60E-03	6	Yes	5.00E-08	2	
van Reij, 2020	Single independent replication cohort from previous published results: CALGB 40101	203	EA	9.00E-03	1	Yes	NS	NS	
Veluchamy, 2021	Single independent replication cohort from previous published results: CALGB 40101	428925	EA	NS	NS	Yes	5.00E-08	1	The SNPs found in meta-analysis were different independent loci from discovery study; One suggestive significant signals in the joint meta-analysis was added to describe in the results section
Warner, 2017	Individual analysis of several replication cohorts	Rotterdam: 212; Nottingham: 908	NS	NS	NS	Yes	NS	NS	
Winsvold, 2021	Single independent replication cohort	383998	EA	NS	NS	Yes	5.00E-08	2	
Won, 2012	Replication was split from the discovery cohort	247	Korean	NS	NS	Yes	1.00E-05	3	
Yokoshima, 2018	No replication	NS	NS	NS	NS	No	NS	NS	

EA, European ancestry; AA, African American. SA, south Asian. NS, not specified.

Table S8. SNPs in linkage disequilibrium ($r^2 > 0.6$) from all included papers.

SNP1	Phenotype and PMID of SNP1	SNP2	Phenotype and PMID of SNP2	r^2
rs10888692	Multisite Chronic Pain [31194737]	rs35072907	Multisite Chronic Pain [33830993]	0.673
rs12030576	Dysmenorrhoea pain [29855537]	rs7523086	Dysmenorrhoea pain [27454463]	0.977
rs3737240	Genetic components of chronic musculoskeletal pain [32587327]	rs59898460	Multisite Chronic Pain [31194737, 33830993]	0.958
rs1491985	Chronic Widespread Pain [33926923]	rs7628207	Multisite Chronic Pain [31194737]	1
rs13107325	Genetic components of chronic musculoskeletal pain [32587327]	rs13135092	Multisite Chronic Pain [31194737, 33830993]	0.895
rs2049604	Shoulder and Neck Pain [32246137]	rs12537376	Multisite Chronic Pain [31194737]	0.636
rs12537376	Multisite Chronic Pain [31194737]	rs12705966	Genetic components of chronic musculoskeletal pain [32587327]	0.693
rs7833174	Chronic back pain [30261039]	rs7814941	Chronic back pain [30747904]	0.919
rs3180	Chronic back pain [30747904]	rs1678626	Chronic back pain [33021770]	0.979
rs12308843	Chronic back pain [33021770]	rs12310519	Chronic back pain [30261039,30747904]	0.616
rs11079993	Multisite Chronic Pain [33830993]	rs12453010	Multisite Chronic Pain [31194737]	0.959
rs4384683	Chronic back pain [30261039]	rs72922230	Chronic back pain [33021770]	0.85

PMID: PubMed Identifier

Supplementary data 1: full list of reported SNPs from all included papers.

PubMed unique identifier	Outcome	SNP	CHR	POS	CHR_band	Function	Mapped genes	Distance to mapped gene	EA	EAF	BETA (SE)	Discovery P-value	Replication P-value	Meta analysis P-value	Comment
19207018	NSAID analgesic	rs2562456	19	21666210	chr19_p12	upstream	*LINC00664*	dist=307	-	-	-	2.30E-10	-	-	
21622719	Opioid analgesia	rs12211463	6	93467158	chr6_q16.1	intergenic	*CASC6;EPHA7*	dist=1067012;dist=482582	-	-	0.077 (0.023)	3.90E-06	7.30E-02	6.60E-04	BETA (SE) from meta-analysis
21622719	Opioid analgesia	rs12948783	17	74499400	chr17_q25.1	intergenic	*RHBDF2;CYGB*	dist=1891;dist=24030	-	-	-0.142 (0.025)	5.80E-07	4.10E-02	1.10E-08	BETA (SE) from meta-analysis
21622719	Opioid analgesia	rs10413396	19	44813702	chr19_q13.31	intergenic	*ZNF235;ZNF112*	dist=4524;dist=17004	-	-	-0.145 (0.041)	3.80E-07	9.90E-01	4.10E-04	BETA (SE) from meta-analysis
21622719	Opioid analgesia	rs13421094	2	158041802	chr2_q24.1	intergenic	*GPD2;GALNT5*	dist=598887;dist=72308	-	-	0.098 (0.025)	6.50E-06	3.67E-01	8.00E-05	BETA (SE) from meta-analysis
21622719	Opioid analgesia	rs7757130	6	113317262	chr6_q21	intergenic	*RFPL4B;LOC101927686*	dist=644764;dist=627475	-	-	-0.116 (0.033)	2.90E-07	7.57E-01	5.40E-04	BETA (SE) from meta-analysis
21622719	Opioid analgesia	rs2473967	6	113479335	chr6_q21	intergenic	*RFPL4B;LOC101927686*	dist=806837;dist=465402	-	-	-0.143 (0.036)	1.30E-10	2.12E-01	6.20E-05	BETA (SE) from meta-analysis
21622719	Opioid analgesia	rs2884129	10	17585149	chr10_p12.33	intergenic	*ST8SIA6;HACD1*	dist=88763;dist=46809	-	-	-0.093	1.90E-06	3.00E-02	7.50E-05	BETA (SE) from meta-analysis

											(0.024)				
21622719	Opioid analgesia	rs7104613	11	14079931	chr11_p15.2	intronic	*SPON1*		-	-	-0.117 (0.035)	2.20E-08	7.59E-01	7.30E-04	BETA (SE) from meta-analysis
22020760	CIPN	rs2338	6	1573613	chr6_p25.3	intergenic	*FOXF2;FOXCUT*	dist=177781;dist=32153	-	-	0.820 (0.179)	4.36E-05	4.25E-03	4.63E-06	BETA (SE) from meta-analysis
22020760	CIPN	rs10486003	7	97229778	chr7_q21.3	intergenic	*SDHAF3;TAC1*	dist=418703;dist=131493	-	-	-1.139 (0.226)	2.04E-05	2.46E-03	4.84E-07	BETA (SE) from meta-analysis
22020760	CIPN	rs830884	5	52020396	chr5_q11.2	intergenic	*LINC02118;PELO*	dist=680653;dist=63378	-	-	-1.139 (0.238)	2.36E-05	5.83E-03	1.74E-06	BETA (SE) from meta-analysis
22843789	CIPN	rs1903216	3	187629503	chr3_q27.3	intergenic	*BCL6;LINC01991*	dist=165990;dist=47060	-	-	0.102 (0.464)	5.60E-06	-	-	Time to event outcome
22843789	CIPN	rs5934683	X	9751474	chrX_p22.2	intergenic	*GPR143;SHROOM2*	dist=17469;dist=3022	-	-	0.476 (0.095)	6.00E-07	-	-	Ordinal outcome
22843789	CIPN	rs7973533	12	24917962	chr12_p12.1	intergenic	*LINC00477;BCAT1*	dist=180860;dist=44996	-	-	-0.416 (0.093)	8.40E-06	-	-	Ordinal outcome
22843789	CIPN	rs2941627	8	106283030	chr8_q23.1	intergenic	*LRP12;ZFPM2*	dist=681778;dist=48117	-	-	0.647 (0.139)	3.50E-06	-	-	Ordinal outcome

22843789	CIPN	rs10771973	12	32792974	chr12_p11.21	intronic	*FGD4*		-	-	0.096 (0.451)	2.60E-06	-	-	Time to event outcome
22843789	CIPN	rs16916932	10	18476276	chr10_p12.33	intronic	*CACNB2*		-	-	0.159 (0.732)	4.30E-06	-	-	Time to event outcome
22843789	CIPN	rs16948748	17	1457839	chr17_p13.3	intronic	*PITPNA*		-	-	0.184 (0.863)	2.70E-06	-	-	Time to event outcome
22843789	CIPN	rs17781082	12	67476327	chr12_q14.3	ncRNA_intronic	*LOC102724421*		-	-	0.102 (0.47)	4.30E-06	-	-	Time to event outcome
22843789	CIPN	rs4737264	8	56111322	chr8_q12.1	intronic	*XKR4*		-	-	0.109 (0.519)	1.90E-06	-	-	Time to event outcome
22843789	CIPN	rs7001034	8	28363378	chr8_p21.1	intronic	*FZD3*		-	-	-0.562 (0.095)	3.10E-09	-	-	Ordinal outcome
22843789	CIPN	rs7349683	4	66197804	chr4_q13.1	exonic	*EPHA5*		-	-	0.100 (0.489)	9.60E-07	-	-	Time to event outcome
22956598	Chronic Widespread Pain	rs7835968	8	136835301	chr8_q24.23	intergenic	*KHDRBS3;LINC02055*	dist=175449;dist=707740	G/A	0.127	0.148 (0.047)	4.26E-06	7.87E-01	1.43E-03	BETA (SE) from meta-analysis
22956598	Chronic Widespread Pain	rs2249104	11	17738564	chr11_p15.1	intergenic	*LOC102723330;MYOD1*	dist=20078;dist=2546	T/C	0.088	0.191 (0.057)	4.57E-06	8.82E-01	8.78E-04	BETA (SE) from meta-analysis

22956598	Chronic Widespread Pain	rs13361160	5	10169823	chr5_p15.2	intergenic	*LINC02112;FAM173B*	dist=265887;dist=55797	C/T	0.435	0.157 (0.031)	1.18E-08	1.61E-01	4.67E-07	BETA (SE) from meta-analysis
22956598	Chronic Widespread Pain	rs17796312	19	40343475	chr19_q13.2	intergenic	*FBL;FCGBP*	dist=6421;dist=10488	G/A	0.331	0.157 (0.037)	9.79E-06	1.66E-01	2.56E-05	BETA (SE) from meta-analysis
22956598	Chronic Widespread Pain	rs8065610	17	15175570	chr17_p12	intergenic	*PMP22;TEKT3*	dist=6880;dist=31559	A/C	0.387	0.077 (0.031)	3.86E-07	1.19E-01	1.20E-02	BETA (SE) from meta-analysis
22956598	Chronic Widespread Pain	rs12132674	1	120321087	chr1_p12	intergenic	*HMGCS2;REG4*	dist=9532;dist=15554	A/G	0.295	0.148 (0.034)	9.29E-07	1.65E-01	1.23E-05	BETA (SE) from meta-analysis
22956598	Chronic Widespread Pain	rs11606304	11	18052248	chr11_p15.1	intronic	*TPH1*		G/T	0.091	-0.248 (0.079)	1.47E-06	9.34E-01	1.64E-03	BETA (SE) from meta-analysis
22956598	Chronic Widespread Pain	rs4837492	9	133054779	chr9_q34.11	intronic	*HMCN2*		C/T	0.045	0.095 (0.032)	2.96E-06	5.68E-01	2.68E-03	BETA (SE) from meta-analysis
22956598	Chronic Widespread Pain	rs524513	18	34945141	chr18_q12.2	intronic	*CELF4*		T/C	0.182	0.122 (0.041)	4.00E-06	5.37E-01	2.75E-03	BETA (SE) from meta-analysis
22956598	Chronic Widespread Pain	rs7680363	4	16047459	chr4_p15.32	intronic	*PROM1*		A/T	0.064	0.239 (0.063)	1.72E-06	6.88E-01	1.52E-04	BETA (SE) from meta-analysis

23183491	Opioid analgesia	rs2952768	2	208494234	chr2_q33.3	intergenic	*METTL21A;LINC01857*	dist=3457;dist=32874	-	-	0.293 (0.059)	8.04E-07	-	-	BETA (SE) and P were from meta-analysis in additive model
23776197	CIPN	rs12699683	7	15133127	chr7_p21.2	intergenic	*DGKB;AGMO*	dist=118725;dist=106816	-	-	1.297 (0.288)	6.65E-06	-	-	
23776197	CIPN	rs10512385	9	111455575	chr9_q31.3	intergenic	*KLF4;ACTL7B*	dist=1203574;dist=161294	-	-	0.948 (0.209)	5.70E-06	-	-	
23776197	CIPN	rs10090117	8	18381235	chr8_p22	intergenic	*NAT2;PSD3*	dist=122512;dist=3578	-	-	0.867 (0.189)	4.23E-06	-	-	
23776197	CIPN	rs17348202	2	222072178	chr2_q36.1	intergenic	*MIR4268;EPHA4*	dist=1300892;dist=210569	-	-	1.579 (0.323)	1.02E-06	-	-	
23776197	CIPN	rs3181157	12	6308523	chr12_p13.31	upstream	*CD9*	dist=350	-	-	1.169 (0.254)	4.05E-06	-	-	
23776197	CIPN	rs1165472	1	56108604	chr1_p32.2	intergenic	*MIR4422HG;LINC01753*	dist=408754;dist=272678	-	-	0.859 (0.185)	3.65E-06	-	-	
23776197	CIPN	rs4141404	22	31675185	chr22_q12.2	UTR3	*LIMK2*	NM_005569:c.*758A>C;NM_016733:c.*758A>C	-	-	0.880 (0.189)	3.22E-06	-	-	

23776197	CIPN	rs10065203	5	14393621	chr5_p15.2	intronic	*TRIO*		-	-	0.920 (0.200)	4.25E-06	-	-	
23776197	CIPN	rs275456	5	6813937	chr5_p15.31	ncRNA_intronic	*LINC02236*		-	-	0.815 (0.175)	3.31E-06	-	-	
23776197	CIPN	rs2947253	15	36049492	chr15_q14	ncRNA_intronic	*DPH6-AS1*		-	-	1.218 (0.265)	4.36E-06	-	-	
24554482	Drug-induced peripheral neuropathy	rs12913269	15	92926705	chr15_q26.1	intergenic	*SLCO3A1; ST8SIA2*	dist=211040;dist=10412	-	-	1.872 (0.366)	3.20E-07	-	-	
24554482	Drug-induced peripheral neuropathy	rs4397851	11	23796711	chr11_p14.3	intergenic	*MIR8054; LUZP2*	dist=355975;dist=721805	-	-	1.668 (0.347)	1.50E-06	-	-	
24554482	Drug-induced peripheral neuropathy	rs8641	6	148869910	chr6_q24.3	UTR3	*SASH1*	NM_001346505:c.*216C>T;NM_001346506:c.*216C>T;NM_015278:c.*216C>T;NM_001346507:c.*216C>T;NM_001346508:c.*216C>T;NM_001	-	-	1.131 (0.25)	5.80E-06	-	-	

								346509:c .*216C>T						
24554482	Drug-induced peripheral neuropathy	rs11540407	12	75900382	chr12_q21.2	exonic	*KRR1*		-	-	0.956 (0.216)	9.60E-06	-	-	
24554482	Drug-induced peripheral neuropathy	rs6873892	5	178647219	chr5_q35.3	intronic	*ADAMTS2*		-	-	1.808 (0.381)	2.10E-06	-	-	
24582949	Fibromyalgia	rs2701106	12	114697547	chr12_q24.21	intergenic	*LINC02459;TBX5*	dist=17972;dist=94188	-	-	0.086 (0.066)	8.94E-06	2.87E-01	1.94E-01	BETA (SE) from meta-analysis
24582949	Fibromyalgia	rs2858166	X	100875273	chrX_q22.1	intergenic	*ARMCX6; ARMCX3*	dist=2282;dist=2847	-	-	0.166 (0.062)	3.61E-06	6.95E-01	8.00E-03	BETA (SE) from meta-analysis
24582949	Fibromyalgia	rs9381682	6	48620238	chr6_p12.3	intergenic	*PTCHD4; MUT*	dist=583813;dist=777835	-	-	-0.329 (0.097)	2.48E-05	1.56E-01	7.38E-04	Strong signal in meta-analysis; BETA (SE) from meta-analysis

24582949	Fibromyalgia	rs10782344	6	156778660	chr6_q25.3	intergenic	*SNORD28B;ARID1B*	dist=78703;dist=320329	-	-	0.231 (0.080)	3.63E-06	5.26E-01	4.00E-03	BETA (SE) from meta-analysis
24582949	Fibromyalgia	rs10821659	10	61793424	chr10_q21.2	intronic	*ANK3*		-	-	-0.236 (0.069)	4.06E-05	1.10E-01	6.22E-04	Strong signal in meta-analysis; BETA (SE) from meta-analysis
24582949	Fibromyalgia	rs11127292	2	2029943	chr2_p25.3	intronic	*MYT1L*		-	-	-0.400 (0.107)	2.60E-05	3.90E-02	1.76E-04	Strong signal in meta-analysis; BETA (SE) from meta-analysis
24582949	Fibromyalgia	rs11923054	3	167051769	chr3_q26.1	exonic	*ZBBX*		-	-	-0.186 (0.066)	3.52E-06	5.80E-01	4.70E-03	BETA (SE) from meta-analysis
24582949	Fibromyalgia	rs12556003	X	138743267	chrX_q27.1	intronic	*MCF2*		-	-	-0.431 (0.116)	2.14E-06	1.32E-01	1.98E-04	BETA (SE) from meta-analysis
24582949	Fibromyalgia	rs12704506	7	89621311	chr7_q21.13	ncRNA_intronic	*STEAP2-AS1*		-	-	0.784 (0.168)	3.20E-06	1.00E-02	9.00E-01	BETA (SE) from discovery analysis
24582949	Fibromyalgia	rs2194390	2	50902931	chr2_p16.3	intronic	*NRXN1*		-	-	-0.211	8.06E-06	7.02E-01	4.90E-02	BETA (SE) from meta-analysis

											(0.1 07)				
24582949	Fibromyalgia	rs2901761	10	95895127	chr10_q23.33	intronic	*PLCE1*		-	-	-0.693 (0.138)	5.11E-07	-	-	BETA (SE) from discovery analysis
24582949	Fibromyalgia	rs6131711	20	15776418	chr20_p12.1	intronic	*MACROD2*		-	-	0.223 (0.067)	9.92E-08	5.96E-01	9.00E-04	BETA (SE) from meta-analysis
24909733	Opioid analgesia	rs10515902	5	165069995	chr5_q34	intergenic	*LOC102546299;LINC01947*	dist=1100006;dist=1262232	A/G	0.029	46.550 (10.071)	3.80E-06	-	-	In AA population
24909733	Opioid analgesia	rs1503245	2	68224744	chr2_p14	intergenic	*LINC01812;C1D*	dist=172050;dist=44588	T/C	0.06	31.390 (6.716)	2.96E-06	-	-	In AA population
24909733	Opioid analgesia	rs1872270	4	88609681	chr4_q22.1	intergenic	*DMP1;IBSP*	dist=24169;dist=111021	T/C	0.34	16.330 (3.448)	2.18E-06	-	-	In AA population
24909733	Opioid analgesia	rs234348	19	40467983	chr19_q13.2	intergenic	*FCGBP;PSMC4*	dist=27450;dist=8929	G/A	0.21	18.280 (4.138)	1.00E-05	-	-	In AA population
24909733	Opioid analgesia	rs4916453	3	196726039	chr3_q29	intergenic	*PIGZ;MELTF*	dist=30297;dist=2573	A/G	0.42	-14.600 (3.299)	9.60E-06	-	-	In EA population
24909733	Opioid analgesia	rs9457743	6	160273699	chr6_q25.3	intergenic	*PNLDC1;MAS1*	dist=31963;dist=54275	G/T	0.36	-15.730 (3.496)	6.83E-06	-	-	In AA population

24909733	Opioid analgesia	rs1875227	16	56598615	chr16_q12.2	upstream	*MT4*	dist=346	T/C	0.015	60.090 (12.265)	9.61E-07	-	-	In AA population
24909733	Opioid analgesia	rs12566055	1	56260814	chr1_p32.2	intergenic	*MIR4422HG;LINC01753*	dist=560964;dist=120468	T/C	0.012	67.450 (15.170)	8.74E-06	-	-	In AA population
24909733	Opioid analgesia	rs10915528	1	4471374	chr1_p36.32	upstream	*LINC01777*	dist=737	C/T	0.075	27.880 (6.261)	8.46E-06	-	-	In AA population
24909733	Opioid analgesia	rs4620271	8	68741477	chr8_q13.2	intergenic	*CPA6;PREX2*	dist=82857;dist=122873	T/C	0.18	18.820 (4.075)	3.86E-06	-	-	In AA population
24909733	Acute postoperative pain	rs1322650	6	44520451	chr6_p21.1	upstream	*LOC105375075*	dist=833	-	-	-	1.21E-06	-	-	In EA population
24909733	Acute postoperative pain	rs736259	6	44508405	chr6_p21.1	intergenic	*CDC5L;LOC105375075*	dist=90244;dist=5493	-	-	-	5.41E-06	-	-	In EA population
24909733	Opioid analgesia	rs7680282	4	48196494	chr4_p12	intronic	*TEC*		T/C	0.23	17.380 (3.767)	3.95E-06	-	-	In EA population
24909733	Opioid analgesia	rs795484	12	118589862	chr12_q24.23	intronic	*TAOK3*		A/G	0.34	17.550 (3.589)	1.01E-06	3.60E-02	2.96E-07	In EA population
24974787	Diabetic neuropathic pain	rs17428041	8	21711431	chr8_p21.3	intergenic	*GFRA2;DOK2*	dist=65085;dist=54953	-	-	-0.400 (0.077)	1.77E-07	-	-	
25710658	CIPN	rs17032980	2	67302743	chr2_p14	intergenic	*LINC01799;LINC01828*	dist=104145;dist=10835	-	-	1.154	3.67E-06	2.00E-04	9.01E-07	

											(0.249)				
25710658	CIPN	rs12786200	11	91979018	chr11_q14.3	intergenic	*DISC1FP1; FAT3*	dist=1330798;dist=106244	-	-	-1.470 (0.28)	1.58E-07	2.00E-02	6.30E-07	
25710658	CIPN	rs7818688	8	96024082	chr8_q22.1	intergenic	*TP53INP1; NDUFAF6*	dist=62467;dist=13121	-	-	1.449 (0.283)	3.05E-07	1.00E-02	5.03E-07	
25710658	CIPN	rs4463516	9	32867481	chr9_p21.1	intergenic	*TMEM215;APTX*	dist=78282;dist=105123	-	-	1.061 (0.214)	6.83E-07	4.00E-03	1.19E-07	
25710658	CIPN	rs924607	5	610093	chr5_p15.33	ncRNA_intronic	*LOC100996325*		-	-	0.888 (0.183)	1.25E-06	4.00E-04	4.68E-08	
26138065	CIPN	rs3125923	1	68858479	chr1_p31.3	intergenic	*WLS;RPE65*	dist=160195;dist=36028	-	-	0.588 (0.145)	5.00E-05	1.70E-03	-	In EA population; BETA (SE) and P were from grade 3–4 vs control
26138065	CIPN	rs1856746	1	207143422	chr1_q32.1	intronic	*FCAMR*		-	-	1.705 (0.325)	1.60E-07	-	-	In AA population

26566055	Opioid analgesia	rs2076222	1	209791929	chr1_q32.2	exonic	*LAMB3*		-	-	-0.388 (0.075)	2.65E-07	-	-	BETA (SE) and P were from meta-analysis in additive model
26629533	Diabetic neuropathic pain	rs6986153	8	108072044	chr8_q23.1	intergenic	*ABRA;ANGPT1*	dist=289572;dist=189666	-	-	0.513 (0.104)	8.02E-07	-	-	in male
26629533	Diabetic neuropathic pain	rs71647933	1	33945601	chr1_p35.1	intronic	*ZSCAN20*		-	-	0.837 (0.163)	2.74E-07	-	-	In female
27060151	CIPN	rs2839629	21	44453022	chr21_q22.3	UTR3	*PKNOX1*	NM_001286258:c.*2811G>A;NM_001320694:c.*2811G>A;NM_004571:c.*2811G>A	-	-	0.637 (0.142)	7.64E-06	8.30E-03	-	
27060151	CIPN	rs2414277	15	54270793	chr15_q21.3	UTR5	*UNC13C*	NM_001329919:c.-34308C>T	-	-	0.652 (0.144)	6.17E-06	2.20E-01	-	
27060151	CIPN	rs10862339	12	82076089	chr12_q21.31	intronic	*PPFIA2*		-	-	0.718 (0.143)	5.47E-07	5.40E-01	-	
27060151	CIPN	rs11145770	9	139427066	chr9_q34.3	intronic	*NOTCH1*		-	-	0.637 (0.144)	9.70E-06	1.30E-01	-	

27143689	CIPN	rs11017056	10	131830640	chr10_q26.3	intergenic	*EBF3;LINC00959*	dist=68549;dist=31522	-	-	0.948 (0.19)	6.36E-07	-	-	
27143689	CIPN	rs1326116	13	26375046	chr13_q12.13	intronic	*ATP8A2*		-	-	1.095 (0.239)	4.57E-06	-	-	
27143689	CIPN	rs875858	16	70775455	chr16_q22.1	intronic	*VAC14*		-	-	1.348 (0.27)	5.88E-07	7.00E-01	-	
27454463	Dysmenorrhoea pain	rs7523086	1	115823387	chr1_p13.2	intergenic	*TSPAN2;NGF*	dist=191266;dist=5150	G/A	0.621	-2.303 (0.305)	4.10E-14	-	-	
27454463	Dysmenorrhoea pain	rs7137301	12	25450816	chr12_p12.1	intergenic	*KRAS;LMNTD1*	dist=46951;dist=178200	T/A	0.453	-2.688 (0.540)	6.40E-07	-	-	
27454463	Dysmenorrhoea pain	rs186838326	2	85715143	chr2_p11.2	intergenic	*SH2D6;PARTICL*	dist=50991;dist=49447	T/C	0.059	-1.645 (0.334)	8.40E-07	-	-	
27454463	Dysmenorrhoea pain	rs201074342	19	56051315	chr19_q13.42	upstream	*SBK2*		I/D	0.896	3.003 (0.596)	4.80E-07	-	-	
27454463	Dysmenorrhoea pain	rs189805869	8	75435311	chr8_q21.11	intergenic	*GDAP1;PI15*		G/A	0.007	3.016 (0.608)	7.10E-07	-	-	
27454463	Dysmenorrhoea pain	rs315934	2	113883706	chr2_q13	intronic	*IL1RN*		T/C	0.796	-2.551 (0.515)	7.40E-07	-	-	

27605156	CIPN	rs61752783	15	89870178	chr15_q26.1	exonic	*POLG*		-	-	-	3.20E-06	-	-	
27605156	CIPN	rs45443101	16	81922781	chr16_q23.3	exonic	*PLCG2*		-	-	-	3.14E-06	-	-	
27605156	CIPN	rs80146384	5	122425052	chr5_q23.2	ncRNA_intronic	*LOC105379152*		-	-	-	7.21E-06	-	-	
27670397	Severe pre-treatment cancer pain	rs3862188	1	247865773	chr1_q44	intergenic	*OR13G1;OR6F1*	dist=29430;dist=9358	-	-	0.626 (0.113)	7.35E-05	1.49E-03	3.45E-08	BETA (SE) from meta-analysis
27764105	Sciatica	rs145901849	15	52640539	chr15_q21.2	intronic	*MYO5A*		T/C	-	1.110 (0.190)	1.34E-08	2.20E-01	-	
27764105	Sciatica	rs71321981	9	14344411	chr9_p22.3	intronic	*NFIB*		AG/A	-	1.120 (0.200)	1.30E-08	4.00E-02	-	
28025368	CPRS	rs300501	12	78588283	chr12_q21.2	intronic	*NAV3*		-	-	-	3.00E-04	6.00E-01	2.50E-03	Top1 loci in discovery and meta-analysis although it insignificant

28051079	Post-operation neuropathic pain	rs7734804	5	164346536	chr5_q34	intergenic	*LOC102546299;LINC01947*	dist=376547;dist=1985691	A/C	-	0.959 (0.243)	5.25E-06	-	7.80E-05	Replication P not listed here as it replicated in two independent cohorts; BETA (SE) from meta-analysis
28051079	Post-operation neuropathic pain	rs12596162	16	87151495	chr16_q24.2	intergenic	*LINC02181;LOC101928708*	dist=53898;dist=94226	A/G	-	0.231 (0.064)	3.53E-06	-	2.80E-04	Replication P not listed here as it replicated in two independent cohorts; BETA (SE) from meta-analysis

28051079	Post-operation neuropathic pain	rs298235	2	157306688	chr2_q24.1	intronic	*GPD2*		A/G	-	1.089 (0.314)	3.41E-06	-	5.32E-04	Replication P not listed here as it replicated in two independent cohorts; BETA (SE) from meta-analysis
28051079	Post-operation neuropathic pain	rs887797	17	64579445	chr17_q24.2	intronic	*PRKCA*		A/G	-	0.392 (0.091)	4.29E-06	-	1.65E-05	Replication P not listed here as it replicated in two independent cohorts; BETA (SE) from meta-analysis
28081371	Temporomandibular Disorder	rs4794106	17	48238294	chr17_q21.33	intergenic	*PPP1R9B; SGCA*	dist=10416;dist=5052	T/C	0.549	0.259 (0.055)	2.60E-06	3.57E-01	-	All; This loci replicated in one of the replication cohort

28081371	Temporomandibular Disorder	rs60249166	13	32084901	chr13_q12.3	intergenic	*B3GLCT;RXFP2*	dist=178490;dist=228778	C/T	0.812	-0.439 (0.08)	3.57E-08	5.20E-02	-	Females
28081371	Temporomandibular Disorder	rs73271865	7	21399327	chr7_p15.3	intergenic	*LINC01162;SP4*	dist=337556;dist=68325	C/T	0.959	-0.582 (0.113)	2.91E-07	-	-	All
28081371	Temporomandibular Disorder	rs1531554	17	79380547	chr17_q25.3	intronic	*BAHCC1*		T/C	0.476	-0.389 (0.07)	2.92E-08	2.00E-03	-	Females
28081371	Temporomandibular Disorder	rs73460075	X	32283492	chrX_p21.1	intronic	*DMD*		G/C	0.964	-0.574 (0.104)	3.79E-08	-	-	All
28317148	CIPN	rs6552496	4	182293083	chr4_q34.3	intergenic	*LINC00290;TEMN3-AS1*	dist=212781;dist=448075	-	-	0.693 (0.155)	7.82E-06	-	-	
28317148	CIPN	rs12521798	5	79185938	chr5_q14.1	intergenic	*CMYA5;LINC01455*	dist=89889;dist=46464	-	-	1.015 (0.220)	3.80E-06	-	-	
28317148	CIPN	rs17748074	18	49947295	chr18_q21.2	intronic	*DCC*		-	-	0.673 (0.151)	8.60E-06	-	-	
28317148	CIPN	rs8060632	16	83625563	chr16_q23.3	intronic	*CDH13*		-	-	0.837 (0.174)	1.58E-06	-	-	
28447608	Dysmenorrhoea pain	rs7523831	1	115824192	chr1_p13.2	intergenic	*TSPAN2;NGF*	dist=192071;dist=4345	C/Not sp	0.543	-0.198	6.94E-06	2.70E-04	1.36E-08	BETA (SE) from meta-analysis

									ecified		(0.035)				
28447608	Dysmenorrhoea pain	rs76518691	10	80918767	chr10_q22.3	intronic	*ZMIZ1*		A/Not specified	0.133	-0.316 (0.052)	2.31E-07	1.55E-03	1.47E-09	BETA (SE) from meta-analysis
28611204	CIPN	rs4757366	11	15844721	chr11_p15.2	intergenic	*LOC102724957;SOX6*	dist=117807;dist=143274	-	-	0.637 (0.132)	1.35E-06	-	-	
28611204	CIPN	rs6601103	5	179657422	chr5_q35.3	intergenic	*RASGEF1C;MAPK9*	dist=21292;dist=3173	-	-	-0.494 (0.108)	4.88E-06	-	-	
28611204	CIPN	rs7314409	12	31912563	chr12_p11.21	intergenic	*AMN1;H3F3C*	dist=30455;dist=31556	-	-	-0.478 (0.103)	3.57E-06	-	-	
28611204	CIPN	rs6463874	7	9107831	chr7_p21.3	intergenic	*NXPH1;PER4*	dist=315238;dist=566069	-	-	-0.755 (0.164)	4.21E-06	-	-	
28611204	CIPN	rs10799121	1	29129932	chr1_p35.3	intergenic	*YTHDF2;OPRD1*	dist=33645;dist=8722	-	-	-0.478 (0.108)	9.27E-06	-	-	
28611204	CIPN	rs3110290	8	106079696	chr8_q22.3	intergenic	*LRP12;ZFPM2*	dist=478444;dist=251451	-	-	0.94 (0.202)	3.15E-06	-	-	

28611204	CIPN	rs2046571	8	122355712	chr8_q24.12	intergenic	*SNTB1;HAS2*	dist=531403;dist=269559	-	-	0.548 (0.117)	3.00E-06	-	-	
28611204	CIPN	rs1161389	6	50598875	chr6_p12.3	intergenic	*DEFB112; TFAP2D*	dist=582511;dist=82382	-	-	-1.05 (0.225)	3.05E-06	-	-	
28611204	CIPN	rs16825286	2	184826553	chr2_q32.1	intergenic	*NUP35;MIR548AE1*	dist=800141;dist=417149	-	-	0.756 (0.151)	5.37E-07	-	-	
28611204	CIPN	rs9641788	7	125952334	chr7_q31.33	intergenic	*LOC101928283;GRM8*	dist=932959;dist=126318	-	-	0.683 (0.151)	5.75E-06	-	-	
28611204	CIPN	rs6013504	20	36583396	chr20_q11.23	intergenic	*VSTM2L;TTI1*	dist=9649;dist=28013	-	-	0.536 (0.115)	3.02E-06	-	-	
28611204	CIPN	rs1816635	18	28619806	chr18_q12.1	intronic	*DSC3*		-	-	-0.734 (0.166)	9.79E-06	-	-	
28611204	CIPN	rs749958	1	7572262	chr1_p36.23	intronic	*CAMTA1*		-	-	0.519 (0.108)	1.60E-06	-	-	
29207912	Opioid analgesia	rs199670311	16	427758	chr16_p13.3	UTR5	*TMEM8A*		-	-	0.133 (0.024)	3.41E-08	-	-	BETA (SE) and P were from meta-analysis in dominant model

29207912	Opioid analgesia	rs4839603	3	143311260	chr3_q24	intronic	*SLC9A9*		-	-	-0.035 (0.008)	4.16E-06	-	-	BETA (SE) and P were from meta-analysis in additive model
29278617	CIPN	rs1858826	7	93349015	chr7_q21.3	intergenic	*MIR4652; TFPI2*	dist=2698;dist=165694	G/A	0.09	-1.238 (0.233)	8.20E-07	7.00E-02	1.10E-07	BETA (SE) from meta-analysis
29278617	CIPN	rs910920	17	701122	chr17_p13.3	intergenic	*MRM3;NXN*	dist=5373;dist=1431	A/G	0.29	-0.562 (0.123)	1.30E-07	2.00E-01	4.90E-06	BETA (SE) from meta-analysis
29278617	CIPN	rs1857798	4	165370126	chr4_q32.3	intergenic	*MARCH1;LINC01207*	dist=65719;dist=305157	C/T	0.35	-0.821 (0.184)	1.00E-02	5.00E-04	8.30E-06	BETA (SE) from meta-analysis
29502940	Opioid analgesia	rs6961071	7	155974701	chr7_q36.3	intergenic	*LOC389602;LOC285889*	dist=213616;dist=255782	-	-	-1.115 (0.217)	2.74E-07	-	-	Recessive model; BETA (SE) and P were from meta-analysis

29502940	Opioid analgesia	rs13093031	3	88859041	chr3_p11.1	intergenic	*C3orf38;EPHA3*	dist=651926;dist=297633	-	-	-2.239 (0.421)	1.06E-07	-	-	BETA (SE) and P were from meta-analysis in recessive model
29855537	Dysmenorrhoea pain	rs12030576	1	115817221	chr1_p13.2	intergenic	*TSPAN2;NGF*	dist=185100;dist=11316	G/T	-	0.419 (0.046)	1.10E-19	-	-	BETA (SE) from discovery meta-analysis
29855537	Dysmenorrhoea pain	rs80111889	2	113567430	chr2_q13	intergenic	*IL1A;IL1B*	dist=24455;dist=19907	T/G	-	0.425 (0.052)	1.90E-16	-	-	BETA (SE) from discovery meta-analysis
29884837	Neuropathy	rs6796803	3	186464107	chr3_q27.3	intergenic	*KNG1;EIF4A2*	dist=1908;dist=37254	-	-	-0.673 (0.116)	6.42E-09	-	-	
29884837	Neuropathy	rs4804217	19	7699347	chr19_p13.2	upstream	*PCP2*	dist=713	-	-	-0.545 (0.092)	2.95E-09	-	-	
29884837	Neuropathy	rs10950641	7	2334386	chr7_p22.3	intronic	*SNX8*		-	-	1.058 (0.14)	3.39E-14	-	-	
29884837	Neuropathy	rs4775319	15	61213564	chr15_q22.2	intronic	*RORA*		-	-	0.464 (0.081)	1.02E-08	-	-	

30261039	Chronic back pain	rs7833174	8	130718772	chr8_q24.21	intergenic	*CCDC26;GSDMC*	dist=26287;dist=41670	T/C	0.77	0.049 (0.007)	1.00E-07	3.70E-07	4.40E-13	BETA (SE) from meta-analysis
30261039	Chronic back pain	rs12310519	12	23975219	chr12_p12.1	intronic	*SOX5*		T/C	0.16	0.068 (0.008)	7.20E-10	5.30E-11	4.50E-19	BETA (SE) from meta-analysis
30261039	Chronic back pain	rs1453867	2	232917899	chr2_q37.1	intronic	*DIS3L2*		T/C	0.65	-0.030 (0.006)	7.70E-08	2.10E-02	3.90E-07	BETA (SE) from meta-analysis
30261039	Chronic back pain	rs4384683	18	50379032	chr18_q21.2	intronic	*DCC*		A/G	0.54	-0.030 (0.006)	3.20E-07	4.20E-05	2.40E-10	BETA (SE) from meta-analysis
30277654	Opioid analgesia	rs12494691	3	16700334	chr3_p24.3	intergenic	*DAZL;PLCL2*	dist=53328;dist=226118	-	-	0.010 (0.002)	3.92E-08	-	-	
30277654	Opioid analgesia	rs1641025	16	8871388	chr16_p13.2	intronic	*ABAT*		-	-	0.360 (0.064)	2.04E-08	-	-	
30431558	Temporomandibular Disorder	rs5862730	4	146211845	chr4_q31.21	intergenic	*OTUD4;LINC02266*	dist=111013;dist=44570	Deletion	0.33	0.351 (0.063)	2.82E-08	4.75E-01	-	All
30431558	Temporomandibular Disorder	rs34612513	3	137541085	chr3_q22.3	intergenic	*LINC01210;CLDN18*	dist=41366;dist=176573	A/Not specified	0.08	1.099 (0.194)	1.49E-08	1.35E-01	-	Male
30431558	Temporomandibul	rs10092633	8	41123732	chr8_p11.21	intronic	*SFRP1*		A/Not	0.03	1.416	2.91E-08	3.30E-01	-	Female

	ar Disorder								sp eci fie d		(0.2 55)				
30506673	CIPN	rs7963521	12	738289 56	chr12_ q21.1	intergenic	*LINC0244 4;LOC100 507377*	dist=226 859;dist= 698000	-	-	0.80 0 (0.2 06)	1.02E-04	6.57E-03	1.05E-05	Two discovery cohort replicated in each other; additive model
30506673	CIPN	rs1045644	14	313550 96	chr14_ q12	exonic	*COCH*		-	-	- 1.02 0 (0.2 57)	7.32E-05	1.79E-03	8.66E-06	Two discovery cohort replicated in each other; additive model
30747904	Chronic back pain	rs7814941	8	130718 859	chr8_q 24.21	intergenic	*CCDC26;G SDMC*	dist=263 74;dist=4 1583	A/ G	0.8	0.04 6 (0.0 07)	3.71E-11	5.32E-05	-	Same variant not reported in the joint meta-analysis
30747904	Chronic back pain	rs3180	10	738206 22	chr10_ q22.1	UTR3	*SPOCK2*	NM_014 767:c.*1 896T>C; NM_001 244950:c .*1896T> C	A/ G	0.4 4	- 0.03 7 (0.0 06)	1.65E-11	6.59E-03	-	Same variant not reported in the joint meta-analysis

30747904	Chronic back pain	rs12310519	12	23975219	chr12_p12.1	intronic	*SOX5*		C/T	0.84	-0.056 (0.007)	3.52E-14	5.00E-05	-	Same variant not reported in the joint meta-analysis
30747904	Chronic back pain	rs1865442	8	69574165	chr8_q13.2	intronic	*C8orf34*		C/T	0.82	-0.052 (0.007)	3.79E-13	1.05E-02	-	Same variant not reported in the joint meta-analysis
30747904	Chronic back pain	rs2672596	10	124226793	chr10_q26.13	intronic	*HTRA1*		G/A	0.74	0.041 (0.006)	8.89E-11	3.98E-02	4.06E-11	
31127053	Diabetic neuropathic pain	rs10555080	19	32043175	chr19_q12	intronic	*THEG5*		A/-	0.36	0.293 (0.057)	6.90E-06	9.80E-03	2.60E-07	BETA (SE) from meta-analysis
31127053	Diabetic neuropathic pain	rs201655918	14	76791316	chr14_q24.3	intergenic	*GPATCH2L;ESRRB*	dist=112161;dist=46374	C/-	0.26	-0.211 (0.061)	6.90E-06	4.00E-02	5.30E-04	BETA (SE) from meta-analysis
31127053	Diabetic neuropathic pain	rs2491019	10	70776987	chr10_q22.1	downstream	*KIF1BP*	dist=248	A/-	0.47	0.223 (0.052)	4.40E-06	8.60E-01	2.00E-05	BETA (SE) from meta-analysis
31127053	Diabetic neuropathic pain	rs13417783	2	167629849	chr2_q24.3	intergenic	*SCN7A;XIRP2*	dist=286368;dist=115148	T/-	0.14	-0.462 (0.068)	1.90E-09	9.00E-04	7.90E-12	BETA (SE) from meta-analysis

31127053	Diabetic neuropathic pain	rs11932946	4	45140214	chr4_p12	intergenic	*GNPDA2; GABRG1*	dist=411563;dist=897573	G/-	0.12	-0.315 (0.077)	9.60E-06	9.80E-01	4.50E-05	BETA (SE) from meta-analysis
31127053	Diabetic neuropathic pain	rs11073752	15	88423051	chr15_q25.3	intronic	*NTRK3*		C/-	0.32	-0.274 (0.055)	2.10E-06	1.10E-01	6.50E-07	BETA (SE) from meta-analysis
31127053	Diabetic neuropathic pain	rs1202660	7	70658177	chr7_q11.22	intronic	*GALNT17*		T/-	0.2	-0.288 (0.061)	6.20E-06	1.60E-01	2.50E-06	BETA (SE) from meta-analysis
31127053	Diabetic neuropathic pain	rs9948095	18	12018665	chr18_p11.21	intronic	*IMPA2*		C/-	0.15	-0.315 (0.069)	3.60E-06	4.60E-01	5.40E-06	BETA (SE) from meta-analysis
31127053	Diabetic neuropathic pain	rs60770880	3	8037416	chr3_p26.1	ncRNA_intronic	*LOC101927394*		A/-	0.21	0.293 (0.057)	5.00E-06	6.20E-01	1.00E-05	BETA (SE) from meta-analysis
31127053	Diabetic neuropathic pain	rs77494074	11	132794801	chr11_q25	intronic	*OPCML*		T/-	0.07	-0.416 (0.095)	1.00E-06	4.10E-01	1.30E-05	BETA (SE) from meta-analysis
31127053	Diabetic neuropathic pain	rs34948558	21	42825856	chr21_q22.3	intronic	*MX1*		A/-	0.27	-0.236 (0.056)	4.70E-06	9.50E-01	3.00E-05	BETA (SE) from meta-analysis
31127053	Diabetic neuropathic pain	rs13265430	8	4165607	chr8_p23.2	intronic	*CSMD1*		A/-	0.09	-0.357 (0.087)	1.00E-06	2.10E-01	4.30E-05	BETA (SE) from meta-analysis

31127053	Diabetic neuropathic pain	rs12988669	2	240275570	chr2_q37.3	intronic	*HDAC4*		C/-	0.16	-0.274 (0.068)	2.70E-06	3.20E-01	5.10E-05	BETA (SE) from meta-analysis
31194737	Multisite Chronic Pain	rs1976423	5	104042643	chr5_q21.2	intergenic	*NUDT12;RAB9BP1*	dist=1144141;dist=392532	A/C	-	-0.014 (0.002)	8.20E-09	-	-	
31194737	Multisite Chronic Pain	rs11786084	8	142651709	chr8_q24.3	intergenic	*MROH5;MIR1302-7*	dist=134379;dist=215894	G/A	-	-0.015 (0.003)	2.30E-08	-	-	
31194737	Multisite Chronic Pain	rs2386584	15	91539572	chr15_q26.1	intergenic	*PRC1;VPS33B*	dist=1691;dist=2074	T/G	-	-0.017 (0.003)	2.80E-11	-	-	
31194737	Multisite Chronic Pain	rs1443914	13	53917230	chr13_q14.3	intergenic	*LINC01065;LINC00558*	dist=191195;dist=472324	T/C	-	0.016 (0.002)	2.80E-11	-	-	
31194737	Multisite Chronic Pain	rs285026	16	77100089	chr16_q23.1	intergenic	*MIR4719;MON1B*	dist=197173;dist=124727	G/T	-	-0.014 (0.003)	1.90E-08	-	-	
31194737	Multisite Chronic Pain	rs11751591	6	33794215	chr6_p21.31	intergenic	*MLN;LINC01016*	dist=22422;dist=63073	G/A	-	0.021 (0.003)	2.70E-10	-	-	
31194737	Multisite Chronic Pain	rs59898460	1	150493004	chr1_q21.3	intergenic	*FALEC;ADAMTSL4*	dist=2496;dist=28841	T/C	-	0.017 (0.003)	9.20E-12	-	-	
31194737	Multisite Chronic Pain	rs12537376	7	114025053	chr7_q31.1	intergenic	*FOXP2*	dist=29999	A/G	-	0.015	1.70E-09	-	-	

											(0.0 03)				
31194737	Multisite Chronic Pain	rs2006281	14	104327 732	chr14_ q32.33	intergenic	*LINC0063 7;C14orf2*	dist=334 6;dist=50 893	C/ T	-	0.01 4 (0.0 02)	3.40E-08	-	-	
31194737	Multisite Chronic Pain	rs11079993	17	503015 52	chr17_ q22	intergenic	*CA10;LINC 01982*	dist=641 75;dist=1 66330	G/ T	-	- 0.01 7 (0.0 03)	5.70E-12	-	-	
31194737	Multisite Chronic Pain	rs6869446	5	655706 07	chr5_q 12.3	intergenic	*LINC0206 5;LINC022 29*	dist=653 33;dist=2 32766	T/ C	-	- 0.01 4 (0.0 03)	9.50E-09	-	-	
31194737	Multisite Chronic Pain	rs10992729	9	961810 75	chr9_q 22.31	intergenic	*C9orf129; FAM120A OS*	dist=723 79;dist=2 7702	C/ T	-	0.01 6 (0.0 03)	1.10E-09	-	-	
31194737	Multisite Chronic Pain	rs197422	1	112317 512	chr1_p 13.2	downstre am	*KCND3*	dist=942	C/ A	-	- 0.01 5 (0.0 03)	2.00E-09	-	-	
31194737	Multisite Chronic Pain	rs7798894	7	215529 95	chr7_p 15.3	UTR3	*SP4*	NM_003 112:c.*2 108A>G; NM_001 326543:c .*2108A> G;NM_0 0132654 2:c.*210 8A>G	A/ T	-	0.01 5 (0.0 03)	1.60E-08	-	-	
31194737	Multisite Chronic Pain	rs7628207	3	497549 70	chr3_p 21.31	UTR3	*AMIGO3*	NM_198 722:c.*4 14A>G	T/ C	-	0.02 0 (0.0 03)	8.40E-10	-	-	

31194737	Multisite Chronic Pain	rs10259354	7	3487414	chr7_p22.2	intronic	*SDK1*		G/A	-	0.015 (0.003)	3.00E-08	-	-	
31194737	Multisite Chronic Pain	rs10888692	1	50991473	chr1_p32.3	intronic	*FAF1*		C/G	-	-0.014 (0.003)	5.30E-09	-	-	
31194737	Multisite Chronic Pain	rs11599236	10	106454672	chr10_q25.1	intronic	*SORCS3*		T/C	-	0.014 (0.003)	3.30E-08	-	-	
31194737	Multisite Chronic Pain	rs11871043	17	43172849	chr17_q21.31	intronic	*NMT1*		T/C	-	0.015 (0.003)	1.70E-09	-	-	
31194737	Multisite Chronic Pain	rs12071912	1	243241614	chr1_q43	ncRNA_intronic	*LINC01347*		C/T	-	-0.015 (0.003)	5.30E-09	-	-	
31194737	Multisite Chronic Pain	rs12435797	14	73797669	chr14_q24.2	intronic	*NUMB*		G/T	-	-0.017 (0.003)	3.70E-08	-	-	
31194737	Multisite Chronic Pain	rs12765185	10	134977077	chr10_q26.3	intronic	*KNDC1*		T/A	-	-0.015 (0.003)	3.90E-08	-	-	
31194737	Multisite Chronic Pain	rs13135092	4	103198082	chr4_q24	intronic	*SLC39A8*		A/G	-	-0.033 (0.004)	1.50E-13	-	-	
31194737	Multisite Chronic Pain	rs13136239	4	140908755	chr4_q31.1	intronic	*MAML3*		G/A	-	0.014	3.60E-08	-	-	

											(0.003)				
31194737	Multisite Chronic Pain	rs17474406	5	122732342	chr5_q23.2	intronic	*CEP120*		G/A	-	-0.049 (0.009)	2.40E-08	-	-	
31194737	Multisite Chronic Pain	rs1946247	5	160836620	chr5_q34	intronic	*GABRB2*		T/G	-	-0.019 (0.004)	4.90E-08	-	-	
31194737	Multisite Chronic Pain	rs2183271	10	21957229	chr10_p12.31	intronic	*MLLT10*		T/C	-	-0.014 (0.003)	3.10E-08	-	-	
31194737	Multisite Chronic Pain	rs2424248	20	19650324	chr20_p11.23	intronic	*SLC24A3*		G/A	-	0.023 (0.004)	3.70E-10	-	-	
31194737	Multisite Chronic Pain	rs28428925	3	107294634	chr3_q13.12	intronic	*BBX*		G/A	-	-0.021 (0.004)	1.40E-09	-	-	
31194737	Multisite Chronic Pain	rs34811474	4	25408838	chr4_p15.2	exonic	*ANAPC4*		G/A	-	0.019 (0.003)	2.70E-11	-	-	
31194737	Multisite Chronic Pain	rs4852567	2	80703379	chr2_p12	intronic	*CTNNA2*		A/G	-	0.015 (0.003)	4.30E-08	-	-	
31194737	Multisite Chronic Pain	rs61883178	11	16317779	chr11_p15.1	intronic	*SOX6*		C/A	-	-0.021 (0.003)	2.00E-10	-	-	

31194737	Multisite Chronic Pain	rs62098013	18	50863861	chr18_q21.2	intronic	*DCC*		G/A	-	-0.017 (0.003)	4.00E-11	-	-	
31194737	Multisite Chronic Pain	rs6478241	9	119252629	chr9_q33.1	intronic	*ASTN2*		A/G	-	0.015 (0.003)	3.10E-09	-	-	
31194737	Multisite Chronic Pain	rs6770476	3	136073920	chr3_q22.3	intronic	*STAG1*		C/T	-	-0.015 (0.003)	9.40E-09	-	-	
31194737	Multisite Chronic Pain	rs6907508	6	34592090	chr6_p21.31	intronic	*C6orf106*		A/G	-	-0.022 (0.004)	1.10E-08	-	-	
31194737	Multisite Chronic Pain	rs6926377	6	145105354	chr6_q24.2	intronic	*UTRN*		A/C	-	-0.016 (0.003)	7.90E-09	-	-	
31194737	Multisite Chronic Pain	rs6966540	7	95727967	chr7_q21.3	intronic	*DYNC1I1*		T/C	-	-0.014 (0.003)	3.30E-08	-	-	
31194737	Multisite Chronic Pain	rs73581580	9	140251458	chr9_q34.3	intronic	*EXD3*		G/A	-	-0.028 (0.004)	5.30E-14	-	-	Original reported SNP identifier 9:140251458_G_A
31196165	Acute post-radiation	rs73633565	X	13477311	chrX_p22.2	intergenic	*LINC01203;EGFL6*	dist=117367;dist=110383	-	-	0.642	8.06E-06	-	-	

	therapy pain										(0.1 43)				
31196165	Acute post-radiation therapy pain	rs16970540	17	333384 47	chr17_ q12	ncRNA_ex onic	*RAD51L3-RFFL*		-	-	0.78 8 (0.1 65)	1.73E-06	-	-	
31196165	Acute post-radiation therapy pain	rs4584690	13	956801 32	chr13_ q32.1	intronic	*ABCC4*		-	-	0.61 5 (0.1 35)	5.46E-06	-	-	
31482140	Chronic knee pain	rs2808772	9	116913 794	chr9_q 32	intergenic	*KIF12;COL 27A1*	dist=524 57;dist=4 031	G/ A	0.5 25	0.00 6 (0.0 01)	1.49E-08	-	-	Replication P not listed here as it replicated in two independent cohorts
31482140	Chronic knee pain	rs143384	20	340257 56	chr20_ q11.22	UTR5	*GDF5*	NM_000 557:c.-48T>C;N M_0013 19138:c.-48T>C	G/ A	0.6	- 0.00 8 (0.0 01)	1.32E-12	-	-	Replication P not listed here as it replicated in two independent cohorts
31903573	Chronic postoper ative pain	rs10459710	15	936805 57	chr15_ q26.1	intergenic	*RGMA;LIN C02207*	dist=481 14;dist=7 19232	-	-	0.73 6 (0.8 93)	1.36E-06	6.40E-01	4.10E-01	BETA (SE) from meta-analysis

31903573	Chronic postoperative pain	rs10194315	2	220845621	chr2_q35	intergenic	*MIR4268; EPHA4*	dist=74335;dist=1437126	-	-	-0.837 (0.825)	6.94E-06	9.90E-01	3.10E-01	BETA (SE) from meta-analysis
31903573	Chronic postoperative pain	rs117119665	15	91074176	chr15_q26.1	intronic	*CRTC3*		-	-	2.421 (0.512)	7.00E-06	3.10E-01	2.26E-06	BETA (SE) from meta-analysis
31903573	Chronic postoperative pain	rs1145324	15	91005365	chr15_q26.1	intronic	*IQGAP1*		-	-	2.386 (0.517)	6.27E-06	4.60E-01	3.93E-06	BETA (SE) from meta-analysis
31903573	Chronic postoperative pain	rs118184265	12	78539743	chr12_q21.2	intronic	*NAV3*		-	-	-143.572 (174.260)	2.31E-06	9.00E-03	4.10E-01	BETA (SE) from meta-analysis
31903573	Chronic postoperative pain	rs1514185	1	61184756	chr1_p32.1	ncRNA_intronic	*LOC101926964*		-	-	0.988 (0.737)	4.21E-06	7.70E-01	1.80E-01	BETA (SE) from meta-analysis
31903573	Chronic postoperative pain	rs4957810	5	108674262	chr5_q21.3	intronic	*PJA2*		-	-	-40.834 (123.049)	6.69E-06	6.80E-01	7.40E-01	BETA (SE) from meta-analysis
31903573	Chronic postoperative pain	rs62281806	3	171962146	chr3_q26.31	intronic	*FNDC3B*		-	-	-0.100 (0.720)	5.59E-07	8.90E-01	8.90E-01	BETA (SE) from meta-analysis
31903573	Chronic postoperative pain	rs75361675	10	98131836	chr10_q24.1	intronic	*TLL2*		-	-	-108.258 (181.106)	2.41E-06	3.10E-01	5.50E-01	BETA (SE) from meta-analysis

31903573	Chronic postoperative pain	rs7894047	10	16845853	chr10_p13	intronic	*RSU1*		-	-	2.302 (1.315)	5.33E-06	7.00E-02	8.00E-02	BETA (SE) from meta-analysis
31903573	Chronic postoperative pain	rs80120866	8	96449430	chr8_q22.1	ncRNA_intronic	*C8orf37-AS1*		-	-	-670.395 (887.453)	2.03E-06	9.80E-01	4.50E-01	BETA (SE) from meta-analysis
32246137	Shoulder And Neck Pain	rs2049604	7	113990352	chr7_q31.1	intergenic	*FOXP2*	dist=64700	-	-	-0.008 (0.002)	3.26E-08	-	3.19E-09	Replication P not listed here as it replicated in two independent cohorts
32246137	Shoulder And Neck Pain	rs12453010	17	50316131	chr17_q22	intergenic	*CA10;LINC01982*	dist=78754;dist=151751	-	-	0.010 (0.001)	1.66E-11	-	2.20E-12	Replication P not listed here as it replicated in two independent cohorts

32246137	Shoulder And Neck Pain	rs62053992	16	72389872	chr16_q22.2	ncRNA_intronic	*LINC01572*		-	-	-0.010 (0.002)	4.50E-08	-	4.36E-09	Replication P not listed here as it replicated in two independent cohorts
32562552	CIPN	rs77526807	9	132123954	chr9_q34.11	intergenic	*LINC01503;LINC00963*	dist=14198;dist=126985	-	-	0.764 (0.235)	1.14E-03	4.32E-04	1.66E-06	
32562552	CIPN	rs74497159	1	101874586	chr1_p21.2	intergenic	*LINC01307;OLFM3*	dist=31717;dist=393537	-	-	0.693 (0.183)	1.59E-04	5.96E-04	3.62E-07	
32562552	CIPN	rs17076837	13	84069170	chr13_q31.1	intergenic	*SLITRK1*	dist=382170	-	-	0.304 (0.138)	2.78E-02	7.72E-06	1.85E-06	
32562552	CIPN	rs78777495	7	40966898	chr7_p14.1	intergenic	*SUGCT;LINC01450*	dist=66532;dist=37379	-	-	0.328 (0.160)	4.00E-02	1.11E-05	2.99E-06	
32562552	CIPN	rs3110366	8	106289521	chr8_q23.1	intergenic	*LRP12;ZFPM2*	dist=688269;dist=41626	-	-	-0.446 (0.122)	2.51E-04	1.20E-03	1.07E-06	
32562552	CIPN	rs6788186	3	166261888	chr3_q26.1	intergenic	*BCHE;LOC105374194*	dist=706628;dist=354583	-	-	0.476 (0.116)	4.11E-05	2.11E-02	5.08E-06	
32562552	CIPN	rs13168251	5	91434132	chr5_q14.3	intergenic	*ARRDC3-AS1;NR2F1-AS1*	dist=717600;dist=1310930	-	-	0.664 (0.151)	1.13E-05	4.18E-02	6.54E-06	

32562552	CIPN	rs11076190	16	57426936	chr16_q21	intergenic	*CX3CL1;CCL17*	dist=7976;dist=11743	-	-	0.453 (0.184)	1.38E-02	3.18E-05	2.55E-06	
32562552	CIPN	rs2188156	22	19639475	chr22_q11.21	intergenic	*LINC00895;SEPT5*	dist=85113;dist=62512	-	-	0.394 (0.203)	5.28E-02	1.90E-05	8.23E-06	
32562552	CIPN	rs777619	6	68858947	chr6_q12	intergenic	*LOC102723883;LOC101928280*	dist=88553;dist=77915	-	-	0.315 (0.129)	1.48E-02	1.54E-04	8.13E-06	
32562552	CIPN	rs10771973	12	32792974	chr12_p11.21	intronic	*FGD4*		-	-	0.203 (0.097)	3.65E-02	3.91E-06	2.15E-06	
32562552	CIPN	rs2060717	7	128380749	chr7_q32.1	intronic	*CALU*		-	-	0.735 (0.195)	1.61E-04	4.35E-03	3.48E-06	
32562552	CIPN	rs57940640	16	57959238	chr16_q21	intronic	*CNGB1*		-	-	0.607 (0.193)	1.64E-03	1.65E-03	8.74E-06	
32562552	CIPN	rs61963755	13	70422662	chr13_q21.33	intronic	*KLHL1*		-	-	0.630 (0.199)	1.56E-03	3.60E-04	1.88E-06	
32562552	CIPN	rs9623812	22	43724686	chr22_q13.2	intronic	*SCUBE1*		-	-	-0.387 (0.106)	2.60E-04	3.70E-03	3.23E-06	
32587327	Genetic components of chronic musculoskeletal pain	rs548227718	5	175902724	chr5_q35.2	intronic	*FAF2*		A/G	0.001	-0.283 (0.048)	4.01E-09	1.06E-01	-	

32587327	Genetic components of chronic musculoskeletal pain	rs111368900	1	53084695	chr1_p32.3	intergenic	*GPX7*		A/G	0.002	0.242 (0.041)	6.60E-09	6.55E-02	-	
32587327	Genetic components of chronic musculoskeletal pain	rs143384	20	34025756	chr20_q11.22	UTR5	*GDF5*	NM_000557:c.-48T>C;NM_001319138:c.-48T>C	T/C	0.585	-0.020 (0.003)	7.40E-13	1.65E-10	-	
32587327	Genetic components of chronic musculoskeletal pain	rs7628207	3	49754970	chr3_p21.31	UTR3	*AMIGO3*	NM_198722:c.*414A>G	C/T	0.818	-0.023 (0.004)	2.37E-10	4.92E-03	-	
32587327	Genetic components of chronic musculoskeletal pain	rs12705966	7	114248851	chr7_q31.1	intronic	*FOXP2*		A/G	0.672	0.018 (0.003)	7.52E-09	1.70E-03	-	
32587327	Genetic components of chronic musculoskeletal pain	rs13107325	4	103188709	chr4_q24	exonic	*SLC39A8*		C/T	0.926	-0.032 (0.005)	1.19E-09	4.21E-08	2.00E-08	

32587327	Genetic components of chronic musculoskeletal pain	rs3737240	1	150483355	chr1_q21.3	exonic	*ECM1*		C/T	0.611	0.017 (0.003)	2.69E-09	3.17E-03	8.40E-10	
32587327	Genetic components of chronic musculoskeletal pain	rs4985445	16	69867835	chr16_q22.1	intronic	*WWP2*		A/G	0.532	0.017 (0.003)	2.09E-09	3.71E-02	-	
32587327	Genetic components of chronic musculoskeletal pain	rs73581580	9	140251458	chr9_q34.3	intronic	*EXD3*		A/G	0.123	0.025 (0.004)	5.15E-09	9.54E-09	-	
32681239	Constant-severe pain in chronic pancreatitis	rs11300774	8	14471244	chr8_p22	intronic	*SGCZ*		T/TA	-	-0.453 (0.099)	4.38E-06	-	-	
33021770	Chronic back pain	rs116007789	7	101223945	chr7_q22.1	intergenic	*LINC01007;MYL10*	dist=11659;dist=32660	C/T	0.998	-0.079 (0.013)	3.30E-09	5.39E-01	-	Female
33021770	Chronic back pain	rs367563576	1	150495378	chr1_q21.3	intergenic	*FALEC;ADAMTSL4*	dist=4870;dist=26467	T/TAC	0.609	0.007 (0.001)	7.60E-09	9.04E-02	-	Female
33021770	Chronic back pain	rs1039325	5	30761421	chr5_p13.3	intergenic	*LOC105374704;CDH6*	dist=879211;dist=432341	T/G	0.417	-0.007	8.70E-09	1.11E-01	-	Female

											(0.0 01)				
33021770	Chronic back pain	rs12308843	12	239744 04	chr12_ p12.1	intronic	*SOX5*		G/ C	0.7 64	- 0.01 0 (0.0 01)	9.40E-15	5.95E-02	-	Female
33021770	Chronic back pain	rs1678626	10	738263 35	chr10_ q22.1	intronic	*SPOCK2*		T/ C	0.4 45	- 0.00 7 (0.0 01)	2.40E-08	2.80E-03	-	Male
33021770	Chronic back pain	rs2391333	13	107166 694	chr13_ q33.3	intronic	*EFNB2*		C/ T	0.6 15	- 0.00 7 (0.0 01)	1.90E-08	2.53E-02	-	Female
33021770	Chronic back pain	rs62327819	4	147211 141	chr4_q 31.22	intronic	*SLC10A7*		C/ T	0.3 22	- 0.00 7 (0.0 01)	8.10E-09	4.80E-03	-	Female
33021770	Chronic back pain	rs72922230	18	503944 07	chr18_ q21.2	intronic	*DCC*		A/ G	0.5 98	- 0.00 7 (0.0 01)	2.40E-08	1.52E-01	-	Male
33021770	Chronic back pain	rs7834973	8	696396 72	chr8_q 13.2	intronic	*C8orf34*		T/ G	0.6 09	- 0.00 7 (0.0 01)	4.20E-09	9.16E-01	-	Female
33685280	Postherp etic neuralgia	rs4773840	13	957704 25	chr13_ q32.1	intronic	*ABCC4*		-	-	-	1.64E-07	-	-	
33802509	CIPN	rs77880756	5	124192 458	chr5_q 23.2	intergenic	*ZNF608;L OC101927 421*	dist=111 653;dist= 180066	-	-	- 9.08 0	5.40E-01	6.54E-07	-	

											(1.7 90)				
33802509	CIPN	rs78825864	12	981695 01	chr12_ q23.1	intergenic	*LOC64371 1;MIR449 5*	dist=192 06;dist=1 63333	-	-	- 9.76 0 (1.9 20)	1.80E-01	5.84E-07	-	
33802509	CIPN	rs1515252	2	146779 243	chr2_q 22.3	intergenic	*TEX41;PA BPC1P2*	dist=944 952;dist= 565382	-	-	- 5.96 0 (1.1 10)	1.18E-07	7.00E-02	-	
33802509	CIPN	rs10769096	11	449432 60	chr11_ p11.2	intronic	*TSPAN18*		-	-	- 7.74 0 (1.4 60)	1.65E-07	8.20E-01	-	
33802509	CIPN	rs11380786 8	4	834393 24	chr4_q 21.22	intronic	*TMEM150 C*		-	-	- 16.1 80 (2.7 80)	7.70E-01	1.27E-08	-	
33802509	CIPN	rs4331859	5	179094 108	chr5_q 35.3	ncRNA_int ronic	*LOC10537 7763*		-	-	- 7.55 0 (1.5 10)	4.00E-02	9.17E-07	-	
33802509	CIPN	rs56360211	10	953744 53	chr10_ q23.33	intronic	*PDE6C*		-	-	- 12.9 20 (2.3 80)	7.92E-08	3.40E-01	-	
33830993	Multisite Chronic Pain	rs62381120	5	120176 330	chr5_q 23.1	intergenic	*PRR16;LO C1024672 26*	dist=153 305;dist= 481915	T/ C	-	- 0.02 1 (0.0 04)	3.50E-08	-	-	Female

33830993	Multisite Chronic Pain	rs34003284	13	53902876	chr13_q14.3	intergenic	*LINC01065;LINC00558*	dist=176841;dist=486678	C/A	-	-0.024 (0.004)	3.20E-10	-	-	Female
33830993	Multisite Chronic Pain	rs59898460	1	150493004	chr1_q21.3	intergenic	*FALEC;ADAMTSL4*	dist=2496;dist=28841	T/C	-	0.025 (0.004)	4.90E-12	-	-	Female
33830993	Multisite Chronic Pain	rs11079993	17	50301552	chr17_q22	intergenic	*CA10;LINC01982*	dist=64175;dist=166330	G/T	-	-0.021 (0.004)	4.50E-09	-	-	Female
33830993	Multisite Chronic Pain	rs16909443	11	6192462	chr11_p15.4	upstream	*OR52B2*	dist=906	T/C	-	-0.040 (0.007)	4.40E-08	-	-	Male
33830993	Multisite Chronic Pain	rs151060048	6	34633069	chr6_p21.31	intronic	*C6orf106*		CA/C	-	-0.035 (0.006)	5.40E-09	-	-	Female
33830993	Multisite Chronic Pain	rs74274428	5	170842428	chr5_q35.1	upstream	*FGF18*		CA/C	-	0.020 (0.004)	2.80E-08	-	-	Female
33830993	Multisite Chronic Pain	18:50442591_TTTC_T	18	50442591	chr18_q21.2	intronic	*DCC*		TTTC/T	-	-0.020 (0.004)	1.60E-08	-	-	Male
33830993	Multisite Chronic Pain	20:19709268_AAAAT_A	20	19709268	chr20_p11.23	intergenic	*SLC24A3;AL121761.1*		AAAAT/A	-	0.030 (0.005)	1.20E-08	-	-	Male
33830993	Multisite Chronic Pain	rs147903676	2	5835359	chr2_p25.2	exonic	*SOX11*		C/CT	-	-0.031	2.00E-08	-	-	Female

											(0.0 06)				
33830993	Multisite Chronic Pain	rs3080365	5	575765 65	chr5_q 11.2	intergenic	*PGAM1P1 ;PLK2*		TA CA C/ T	-	0.02 4 (0.0 04)	2.90E-08	-	-	Female; rs3080367 has merged into rs3080365
33830993	Multisite Chronic Pain	rs10660361	6	337413 71	chr6_p 21.31	intronic	*LEMD2*		C/ CG	-	0.02 0 (0.0 04)	1.80E-08	-	-	Male
33830993	Multisite Chronic Pain	rs13135092	4	103198 082	chr4_q 24	intronic	*SLC39A8*		A/ G	-	- 0.03 8 (0.0 06)	2.30E-09	-	-	Female
33830993	Multisite Chronic Pain	rs35072907	1	511895 56	chr1_p 32.3	intronic	*FAF1*		G/ C	-	0.02 0 (0.0 04)	2.40E-08	-	-	Female
33830993	Multisite Chronic Pain	rs73581580	9	140251 458	chr9_q 34.3	intronic	*EXD3*		G/ A	-	- 0.03 0 (0.0 05)	3.00E-09	-	-	Male; originally reported SNP identifier 9:140251458_G _A

33926923	Chronic Widespread Pain	rs165599	22	19956781	chr22_q11.21	UTR3	*COMT*	NM_000754:c.*522G>A;NM_001135161:c.*522G>A;NM_001135162:c.*522G>A;NM_007310:c.*522G>A	G/A	0.3	-0.003 (0.001)	2.50E-08	5.00E-01	-	Replication meta-analysis P-value from standard-error based analysis
33926923	Chronic Widespread Pain	rs10490825	3	130696383	chr3_q22.1	intronic	*ATP2C1*		G/A	0.87	-0.004 (0.001)	1.30E-08	4.90E-02	-	Replication meta-analysis P-value from standard-error based analysis
33926923	Chronic Widespread Pain	rs1491985	3	49739507	chr3_p21.31	intronic	*RNF123*		G/C	0.18	0.003 (0.001)	1.60E-08	3.00E-04	-	Replication meta-analysis P-value from standard-error based analysis
34391895	CIPN	rs2181623	10	6391491	chr10_p15.1	intergenic	*LOC399715;PRKCQ*	dist=13548;dist=44568	-	-	1.581 (0.354)	8.10E-06	-	-	Extreme phenotype
34391895	CIPN	rs7169642	15	82360813	chr15_q25.2	intergenic	*MEX3B;LINC01583*	dist=22329;dist=20122	-	-	-0.494	3.30E-06	-	-	Standard phenotype

											(0.106)				
34391895	CIPN	rs76299149	3	116973739	chr3_q13.31	intergenic	*LINC00901;LINC02024*	dist=322654;dist=423801	-	-	0.542 (0.119)	5.60E-06	-	-	Standard phenotype
34391895	CIPN	rs12501594	4	181526148	chr4_q34.3	intergenic	*LINC00290*	dist=459095	-	-	1.128 (0.243)	3.60E-06	-	-	Standard phenotype
34391895	CIPN	rs9476901	6	15731306	chr6_p22.3	intergenic	*DTNBP1; MYLIP*	dist=68017;dist=398011	-	-	0.451 (0.101)	8.40E-06	-	-	Standard phenotype
34391895	CIPN	rs112917429	9	36101017	chr9_p13.3	intronic	*RECK*		-	-	0.846 (0.184)	4.30E-06	-	-	Standard phenotype
34391895	CIPN	rs117036130	13	52551794	chr13_q14.3	intronic	*ATP7B*		-	-	-1.609 (0.353)	5.10E-06	-	-	Extreme phenotype
34391895	CIPN	rs17610383	18	39892281	chr18_q12.3	ncRNA_intronic	*LINC00907*		-	-	0.833 (0.174)	1.60E-06	-	-	Standard phenotype
34391895	CIPN	rs6829206	4	47052606	chr4_p12	intronic	*GABRB1*		-	-	0.761 (0.162)	2.70E-06	-	-	Standard phenotype

34854908	Neuropathic pain	rs369920026	12	98585582	chr12_q23.1	intergenic	*MIR4303; SLC9A7P1*	dist=196356;dist=262037	A/G	0.006	0.519 (0.091)	1.73E-05	1.29E-04	1.30E-08	P-values were from stage1; UKB;and stage 2 analysis; respectively; BETA (SE) from meta-analysis
34854908	Neuropathic pain	rs112990863	3	88714964	chr3_p11.1	intergenic	*EPHA3*		T/A	0.007	0.378 (0.085)	3.73E-08	9.60E-01	8.99E-06	P-values were from stage1; UKB;and stage 2 analysis; respectively; BETA (SE) from meta-analysis

34854908	Neuropathic pain	rs7992766	13	49905672	chr13_q14.2	intronic	*CAB39L*		A/C	0.75	0.086 (0.016)	2.41E-05	9.00E-04	1.22E-07	P-values were from stage1; UKB;and stage 2 analysis; respectively; BETA (SE) from meta-analysis
34924555	Pain sensitivity questionnaire score	rs142738119	18	10008669	chr18_p11.22	intergenic	*VAPA;LINC01254*	dist=48651;dist=396461	-	-	0.696 (0.138)	4.70E-07	9.00E-01	-	Validation p-value from CPT
34924555	Cold pressor test	rs141828201	17	65520139	chr17_q24.2	intronic	*PITPNC1*		-	-	0.356 (0.066)	2.40E-07	2.60E-01	-	Validation p-value from PSQ
34924555	Pain sensitivity questionnaire score	rs12583902	13	101893919	chr13_q33.1	intronic	*NALCN*		-	-	0.059 (0.012)	7.60E-07	2.90E-01	-	Validation p-value from CPT
34924555	Pain sensitivity questionnaire score	rs58194899	2	3280983	chr2_p25.3	intronic	*EIPR1*		-	-	-0.051 (0.009)	1.90E-08	5.80E-01	-	Validation p-value from CPT
34975738	Idiopathic polyneuropathy	rs147738081	1	199854673	chr1_q32.1	intergenic	*LINC01221;NR5A2*	dist=808809;dist=142057	T/C	0.031	0.519 (0.095)	2.13E-05	4.14E-04	4.75E-08	BETA (SE) from meta-analysis

34975738	Idiopathic polyneuropathy	rs7294354	12	588944	chr12_p13.33	intronic	*B4GALNT3*		T/G	0.428	0.174 (0.032)	1.50E-04	7.51E-05	4.51E-08	BETA (SE) from meta-analysis

Supplementary data 2: Publication list in the Human Pain Genetics Database (HPGDB) of overlapping genes between genes identified in this review and the HPGDB.

SNP	Function	Mapped (nearby) gene	Phenotype	Publication
rs1800587	UTR5	*IL1A*	Migraine	[1]
rs1042114	exonic	*OPRD1*	Nociception	[2]
rs4633	exonic	*COMT*	Temporomandibular Disorder	[3]
rs4633	exonic	*COMT*	Nociception	[3]
rs4633	exonic	*COMT*	Nociception	[3]
rs4633	exonic	*COMT*	Nociception	[3]
rs4680	exonic	*COMT*	Temporomandibular Disorder	[3]
rs4680	exonic	*COMT*	Nociception	[3]
rs4680	exonic	*COMT*	Nociception	[3]
rs4680	exonic	*COMT*	Nociception	[3]
rs4818	exonic	*COMT*	Nociception	[3]
rs4818	exonic	*COMT*	Temporomandibular Disorder	[3]
rs4818	exonic	*COMT*	Nociception	[3]
rs4818	exonic	*COMT*	Nociception	[3]
rs4818	exonic	*COMT*	Nociception	[3]
rs6269	intronic	*COMT*	Nociception	[3]
rs6269	intronic	*COMT*	Temporomandibular Disorder	[3]
rs6269	intronic	*COMT*	Nociception	[3]
rs6269	intronic	*COMT*	Nociception	[3]
rs6269	intronic	*COMT*	Nociception	[3]
rs4680	exonic	*COMT*	Analgesia	[4]
rs4633	exonic	*COMT*	Nociception	[5]
rs4680	exonic	*COMT*	Nociception	[5]
rs4680	exonic	*COMT*	Nociception	[5]
rs4818	exonic	*COMT*	Nociception	[5]
rs6269	intronic	*COMT*	Nociception	[5]
rs6269	intronic	*COMT*	Nociception	[6]

rs4646312	intronic	*COMT*	Nociception	[6]
rs4680	exonic	*COMT*	Analgesia	[7]
rs1800532	intronic	*TPH1*	Migraine	[8]
rs6290	exonic	*GABRB1*	Neuraxial Pain	[9]
rs4694846	intronic	*GABRB1*	Neuraxial Pain	[9]
rs6813436	intronic	*GABRB1*	Neuraxial Pain	[9]
rs7439087	intronic	*GABRB1*	Neuraxial Pain	[9]
rs13107066	intronic	*GABRB1*	Neuraxial Pain	[9]
rs17461905	intronic	*GABRB1*	Neuraxial Pain	[9]
rs4633	exonic	*COMT*	Fibromyalgia	[10]
rs4680	exonic	*COMT*	Fibromyalgia	[10]
rs4818	exonic	*COMT*	Fibromyalgia	[10]
rs6269	intronic	*COMT*	Fibromyalgia	[10]
rs4633	exonic	*COMT*	Analgesia	[11]
rs4680	exonic	*COMT*	Analgesia	[11]
rs6269	intronic	*COMT*	Analgesia	[11]
rs174680	intronic	*COMT*	Analgesia	[11]
rs174699	intronic	*COMT*	Analgesia	[11]
rs737866	intronic	*COMT*	Analgesia	[11]
rs740603	intronic	*COMT*	Analgesia	[11]
rs740603	intronic	*COMT*	Analgesia	[11]
rs2239393	intronic	*COMT*	Analgesia	[11]
rs5746849	intronic	*COMT*	Analgesia	[11]
rs7287550	intronic	*COMT*	Analgesia	[11]
rs7290221	intronic	*COMT*	Analgesia	[11]
rs165728	UTR3	*COMT*	Analgesia	[11]
rs7897594	intergenic	*SLC39A12;CACNB2*	Migraine	[12]
rs11596974	intergenic	*SLC39A12;CACNB2*	Migraine	[12]
rs1409202	intronic	*CACNB2*	Migraine	[12]
rs7076100	intronic	*CACNB2*	Migraine	[12]
rs8181477	intronic	*CACNB2*	Migraine	[12]

rs11014504	intronic	*CACNB2*	Migraine	[12]
rs11598027	intronic	*CACNB2*	Migraine	[12]
rs4680	exonic	*COMT*	Analgesia	[13]
rs4818	exonic	*COMT*	Analgesia	[13]
rs4680	exonic	*COMT*	Analgesia	[14]
rs4680	exonic	*COMT*	Fibromyalgia	[15]
rs1143634	exonic	*IL1B*	Migraine	[16]
rs4633	exonic	*COMT*	Analgesia	[17]
rs4680	exonic	*COMT*	Analgesia	[17]
rs4818	exonic	*COMT*	Analgesia	[17]
rs6269	intronic	*COMT*	Analgesia	[17]
rs4680	exonic	*COMT*	Fibromyalgia	[18]
rs4680	exonic	*COMT*	Nociception	[19]
rs4633	exonic	*COMT*	Other Clinical Pain	[20]
rs4680	exonic	*COMT*	Other Clinical Pain	[20]
rs4818	exonic	*COMT*	Other Clinical Pain	[20]
rs6269	intronic	*COMT*	Other Clinical Pain	[20]
rs4633	exonic	*COMT*	Musculoskeletal Pain	[21]
rs4680	exonic	*COMT*	Musculoskeletal Pain	[21]
rs4818	exonic	*COMT*	Musculoskeletal Pain	[21]
rs6269	intronic	*COMT*	Musculoskeletal Pain	[21]
rs4818	exonic	*COMT*	Post-operative Pain	[22]
rs6269	intronic	*COMT*	Post-operative Pain	[22]
rs4680	exonic	*COMT*	Neuraxial Pain	[23]
rs4680	exonic	*COMT*	Fibromyalgia	[24]
rs4818	exonic	*COMT*	Fibromyalgia	[24]
rs4680	exonic	*COMT*	Fibromyalgia	[25]
rs4680	exonic	*COMT*	Analgesia	[26]
rs4680	exonic	*COMT*	Analgesia	[26]
rs4680	exonic	*COMT*	Fibromyalgia	[27]
rs1146161	intergenic	*TSPAN2;NGF*	Migraine	[28]

rs4861775	intergenic	*LINC01098;LINC00290*	Migraine	[28]
rs4633	exonic	*COMT*	Analgesia	[29]
rs4680	exonic	*COMT*	Analgesia	[29]
rs165722	intronic	*COMT*	Analgesia	[29]
rs2078371	intergenic	*TSPAN2;NGF*	Migraine	[30]
rs1143634	exonic	*IL1B*	Neuraxial Pain	[31]
rs4680	exonic	*COMT*	Fibromyalgia	[32]
rs4680	exonic	*COMT*	Cancer Pain	[33]
rs2072100	intronic	*TAC1*	Temporomandibular Disorder	[34]
rs174697	intronic	*COMT*	Temporomandibular Disorder	[34]
rs2236857	intronic	*OPRD1*	Temporomandibular Disorder	[34]
rs11466066	upstream	*NGF*	Temporomandibular Disorder	[34]
rs4680	exonic	*COMT*	Nociception	[35]
rs4818	exonic	*COMT*	Other Clinical Pain	[36]
rs1544325	intronic	*COMT*	Temporomandibular Disorder	[37]
rs5993882	intronic	*COMT*	Temporomandibular Disorder	[37]
rs5993883	intronic	*COMT*	Temporomandibular Disorder	[37]
rs4680	exonic	*COMT*	Neuraxial Pain	[38]
rs9524885	intronic	*ABCC4*	Cancer Pain	[39]
rs4633	exonic	*COMT*	Fibromyalgia	[40]
rs4633	exonic	*COMT*	Fibromyalgia	[40]
rs4680	exonic	*COMT*	Fibromyalgia	[40]
rs4680	exonic	*COMT*	Fibromyalgia	[40]
rs4818	exonic	*COMT*	Fibromyalgia	[40]
rs6269	intronic	*COMT*	Fibromyalgia	[40]
rs4633	exonic	*COMT*	Neuraxial Pain	[41]
rs4680	exonic	*COMT*	Neuraxial Pain	[41]

rs6478241	intronic	*ASTN2*	Migraine	[42]
rs4633	exonic	*COMT*	Analgesia	[43]
rs4680	exonic	*COMT*	Analgesia	[43]
rs4680	exonic	*COMT*	Analgesia	[43]
rs4818	exonic	*COMT*	Analgesia	[43]
rs4818	exonic	*COMT*	Analgesia	[43]
rs6269	intronic	*COMT*	Analgesia	[43]
rs4680	exonic	*COMT*	Musculoskeletal Pain	[44]
rs4680	exonic	*COMT*	Fibromyalgia	[44]
rs4680	exonic	*COMT*	Fibromyalgia	[45]
rs4680	exonic	*COMT*	Analgesia	[46]
rs4680	exonic	*COMT*	Post-operative Pain	[47]
rs2078371	intergenic	*TSPAN2;NGF*	Migraine	[48]
rs2078371	intergenic	*TSPAN2;NGF*	Migraine	[48]
rs4680	exonic	*COMT*	Analgesia	[49]
rs165656	intronic	*COMT*	Temporomandibular Disorder	[50]
rs165722	intronic	*COMT*	Temporomandibular Disorder	[50]
rs2071375	intronic	*IL1A*	Neuraxial Pain	[51]
rs4680	exonic	*COMT*	Analgesia	[52]
rs4633	exonic	*COMT*	Nociception	[53]
rs4680	exonic	*COMT*	Nociception	[53]
rs4818	exonic	*COMT*	Nociception	[53]
rs6269	intronic	*COMT*	Nociception	[53]
rs143383	intronic	*GDF5*	Neuraxial Pain	[54]
rs4680	exonic	*COMT*	Migraine	[55]
rs10915437	intergenic	*LINC01346;LINC01777*	Migraine	[56]
rs12134493	intergenic	*TSPAN2;NGF*	Migraine	[56]
rs4379368	intronic	*SUGCT*	Migraine	[56]
rs6478241	intronic	*ASTN2*	Migraine	[56]
rs1143627	upstream	*IL1B*	Cancer Pain	[57]

rs4633	exonic	*COMT*	Musculoskeletal Pain	[58]
rs4680	exonic	*COMT*	Musculoskeletal Pain	[58]
rs4818	exonic	*COMT*	Musculoskeletal Pain	[58]
rs6269	intronic	*COMT*	Musculoskeletal Pain	[58]
rs737865	intronic	*COMT*	Musculoskeletal Pain	[58]
rs4680	exonic	*COMT*	Musculoskeletal Pain	[59]
rs4680	exonic	*COMT*	Musculoskeletal Pain	[59]
rs4633	exonic	*COMT*	Neuraxial Pain	[60]
rs4633	exonic	*COMT*	Neuraxial Pain	[60]
rs4633	exonic	*COMT*	Neuraxial Pain	[60]
rs4680	exonic	*COMT*	Post-operative Pain	[60]
rs4680	exonic	*COMT*	Neuraxial Pain	[60]
rs4680	exonic	*COMT*	Neuraxial Pain	[60]
rs4680	exonic	*COMT*	Neuraxial Pain	[60]
rs4680	exonic	*COMT*	Neuraxial Pain	[60]
rs4818	exonic	*COMT*	Neuraxial Pain	[60]
rs4818	exonic	*COMT*	Neuraxial Pain	[60]
rs4818	exonic	*COMT*	Neuraxial Pain	[60]
rs4818	exonic	*COMT*	Neuraxial Pain	[60]
rs6269	intronic	*COMT*	Neuraxial Pain	[60]
rs6269	intronic	*COMT*	Neuraxial Pain	[60]
rs2072100	intronic	*TAC1*	Temporomandibular Disorder	[61]
rs4680	exonic	*COMT*	Other Clinical Pain	[62]
rs1800587	UTR5	*IL1A*	Nociception	[63]
rs1800587	UTR5	*IL1A*	Neuraxial Pain	[63]
rs4680	exonic	*COMT*	Fibromyalgia	[64]
rs165774	intronic	*COMT*	Nociception	[65]
rs4633	exonic	*COMT*	Nociception	[66]
rs4633	exonic	*COMT*	Nociception	[66]
rs4680	exonic	*COMT*	Nociception	[66]
rs4680	exonic	*COMT*	Nociception	[66]

rs4818	exonic	*COMT*	Nociception	[66]
rs4818	exonic	*COMT*	Nociception	[66]
rs6269	intronic	*COMT*	Nociception	[66]
rs6269	intronic	*COMT*	Nociception	[66]
rs4633	exonic	*COMT*	Post-operative Pain	[67]
rs4633	exonic	*COMT*	Post-operative Pain	[67]
rs4633	exonic	*COMT*	Post-operative Pain	[67]
rs4680	exonic	*COMT*	Post-operative Pain	[67]
rs4680	exonic	*COMT*	Post-operative Pain	[67]
rs4818	exonic	*COMT*	Post-operative Pain	[67]
rs4818	exonic	*COMT*	Post-operative Pain	[67]
rs6269	intronic	*COMT*	Post-operative Pain	[67]
rs6269	intronic	*COMT*	Post-operative Pain	[67]
rs16944	upstream	*IL1B*	Musculoskeletal Pain	[68]
rs1143627	upstream	*IL1B*	Musculoskeletal Pain	[68]
rs1143634	exonic	*IL1B*	Neuraxial Pain	[69]
rs1143634	exonic	*IL1B*	Cancer Pain	[70]
rs4680	exonic	*COMT*	Fibromyalgia	[71]
rs6267	exonic	*COMT*	Other Clinical Pain	[72]
rs10915437	intergenic	*LINC01346;LINC01777*	Migraine	[73]
rs12134493	intergenic	*TSPAN2;NGF*	Migraine	[73]
rs4379368	intronic	*SUGCT*	Migraine	[73]
rs4633	exonic	*COMT*	Neuraxial Pain	[74]
rs165656	intronic	*COMT*	Neuraxial Pain	[74]
rs4680	exonic	*COMT*	Fibromyalgia	[75]
rs4680	exonic	*COMT*	Other Clinical Pain	[76]
rs4680	exonic	*COMT*	Migraine	[77]
rs4680	exonic	*COMT*	Analgesia	[78]
rs1800587	UTR5	*IL1A*	Neuraxial Pain	[79]
rs2234677	UTR5	*IL1RN*	Neuraxial Pain	[79]
rs4680	exonic	*COMT*	Musculoskeletal Pain	[80]

rs4680	exonic	*COMT*	Temporomandibular Disorder	[80]
rs737865	intronic	*COMT*	Musculoskeletal Pain	[80]
rs12134493	intergenic	*TSPAN2;NGF*	Migraine	[81]
rs4633	exonic	*COMT*	Analgesia	[82]
rs4680	exonic	*COMT*	Analgesia	[82]
rs4818	exonic	*COMT*	Analgesia	[82]
rs6269	intronic	*COMT*	Analgesia	[82]
rs4633	exonic	*COMT*	Post-operative Pain	[83]
rs4633	exonic	*COMT*	Post-operative Pain	[83]
rs4818	exonic	*COMT*	Post-operative Pain	[83]
rs6269	intronic	*COMT*	Post-operative Pain	[83]
rs6269	intronic	*COMT*	Post-operative Pain	[83]
rs2952768	intergenic	*METTL21A;LINC01857*	Analgesia	[84]
rs12134493	intergenic	*TSPAN2;NGF*	Migraine	[85]
rs143383	intronic	*GDF5*	Temporomandibular Disorder	[86]
rs4633	exonic	*COMT*	Neuraxial Pain	[87]
rs4680	exonic	*COMT*	Neuraxial Pain	[87]
rs4818	exonic	*COMT*	Neuraxial Pain	[87]
rs6269	intronic	*COMT*	Neuraxial Pain	[87]
rs4633	exonic	*COMT*	Analgesia	[88]
rs4680	exonic	*COMT*	Analgesia	[88]
rs4818	exonic	*COMT*	Analgesia	[88]
rs2234918	exonic	*OPRD1*	Analgesia	[89]
rs419335	intronic	*OPRD1*	Analgesia	[89]
rs4633	exonic	*COMT*	Temporomandibular Disorder	[90]
rs4680	exonic	*COMT*	Temporomandibular Disorder	[90]
rs4818	exonic	*COMT*	Temporomandibular Disorder	[90]
rs6269	intronic	*COMT*	Temporomandibular Disorder	[90]

rs165774	intronic	*COMT*	Temporomandibular Disorder	[91]
rs543844	intergenic	*CDC5L;LOC105375075*	Migraine	[92]
rs4379368	intronic	*SUGCT*	Migraine	[92]
rs6478241	intronic	*ASTN2*	Migraine	[92]
rs7068341	UTR3	*RSU1*	Migraine	[92]
rs4379368	intronic	*SUGCT*	Migraine	[93]
rs4680	exonic	*COMT*	Nociception	[94]
rs4680	exonic	*COMT*	Analgesia	[95]
rs4633	exonic	*COMT*	Cancer Pain	[96]
rs4680	exonic	*COMT*	Cancer Pain	[96]
rs4680	exonic	*COMT*	Cancer Pain	[96]
rs4818	exonic	*COMT*	Cancer Pain	[96]
rs6269	intronic	*COMT*	Cancer Pain	[96]
rs1676303	intergenic	*ESRRB;VASH1*	Temporomandibular Disorder	[97]
rs2860216	intergenic	*ESRRB;VASH1*	Temporomandibular Disorder	[97]
rs4903399	intergenic	*GPATCH2L;ESRRB*	Temporomandibular Disorder	[97]
rs4903419	intergenic	*ESRRB;VASH1*	Temporomandibular Disorder	[97]
rs745011	intronic	*ESRRB*	Temporomandibular Disorder	[97]
rs1077430	intronic	*ESRRB*	Temporomandibular Disorder	[97]
rs6574293	intronic	*ESRRB*	Temporomandibular Disorder	[97]
rs10132091	intronic	*ESRRB*	Temporomandibular Disorder	[97]
rs4680	exonic	*COMT*	Migraine	[98]
rs4818	exonic	*COMT*	Fibromyalgia	[99]
rs4680	exonic	*COMT*	Analgesia	[100]
rs2078371	intergenic	*TSPAN2;NGF*	Migraine	[101]
rs6693567	intergenic	*FALEC;ADAMTSL4*	Migraine	[101]

rs6478241	intronic	*ASTN2*	Migraine	[101]
rs186166891	intronic	*SUGCT*	Migraine	[101]
rs4814864	intronic	*SLC24A3*	Migraine	[101]
rs10786156	intronic	*PLCE1*	Migraine	[101]
rs4680	exonic	*COMT*	Post-operative Pain	[102]
rs4680	exonic	*COMT*	Post-operative Pain	[103]
rs1143634	exonic	*IL1B*	Analgesia	[103]
rs6269	intronic	*COMT*	Temporomandibular Disorder	[104]
rs165774	intronic	*COMT*	Temporomandibular Disorder	[104]
rs4379368	intronic	*SUGCT*	Migraine	[105]
rs6478241	intronic	*ASTN2*	Migraine	[105]
rs17561	exonic	*IL1A*	Neuraxial Pain	[106]
rs1304037	UTR3	*IL1A*	Neuraxial Pain	[106]
rs2856836	UTR3	*IL1A*	Neuraxial Pain	[106]
rs1800587	UTR5	*IL1A*	Neuraxial Pain	[106]
rs4680	exonic	*COMT*	Analgesia	[107]
rs2234918	exonic	*OPRD1*	Analgesia	[107]
rs533123	intronic	*OPRD1*	Analgesia	[107]
rs4680	exonic	*COMT*	Cancer Pain	[108]
rs4818	exonic	*COMT*	Cancer Pain	[108]
rs4680	exonic	*COMT*	Analgesia	[109]
rs1676303	intergenic	*ESRRB;VASH1*	Temporomandibular Disorder	[110]
rs165774	intronic	*COMT*	Nociception	[111]
rs4633	exonic	*COMT*	Other Clinical Pain	[112]
rs4633	exonic	*COMT*	Other Clinical Pain	[112]
rs4633	exonic	*COMT*	Other Clinical Pain	[112]
rs4680	exonic	*COMT*	Other Clinical Pain	[112]
rs4680	exonic	*COMT*	Other Clinical Pain	[112]
rs4818	exonic	*COMT*	Other Clinical Pain	[112]
rs4818	exonic	*COMT*	Other Clinical Pain	[112]

rs6269	intronic	*COMT*	Other Clinical Pain	[112]
rs6269	intronic	*COMT*	Other Clinical Pain	[112]
rs165599	UTR3	*COMT*	Other Clinical Pain	[112]
rs165599	UTR3	*COMT*	Other Clinical Pain	[112]
rs165599	UTR3	*COMT*	Other Clinical Pain	[112]
rs4680	exonic	*COMT*	Neuropathic pain	[113]
rs4818	exonic	*COMT*	Neuropathic pain	[113]
rs6269	intronic	*COMT*	Neuropathic pain	[113]
rs1277441	intronic	*TAOK3*	Post-operative pain	[114]
rs4680	exonic	*COMT*	Temporomandibular disorders	[115]
rs4680	exonic	*COMT*	Analgesia	[116]
rs4633	exonic	*COMT*	Post-operative pain	[117]
rs4633	exonic	*COMT*	Post-operative pain	[117]
rs4680	exonic	*COMT*	Post-operative pain	[117]
rs4680	exonic	*COMT*	Post-operative pain	[117]
rs4818	exonic	*COMT*	Post-operative pain	[117]
rs4818	exonic	*COMT*	Post-operative pain	[117]
rs569356	intergenic	*YTHDF2;OPRD1*	Post-operative pain	[117]
rs6269	intronic	*COMT*	Post-operative pain	[117]
rs6269	intronic	*COMT*	Post-operative pain	[117]
rs740603	intronic	*COMT*	Post-operative pain	[117]
rs4818	exonic	*COMT*	Temporomandibular disorders	[118]
rs4633	exonic	*COMT*	Post-operative pain	[119]
rs4680	exonic	*COMT*	Post-operative pain	[119]
rs4818	exonic	*COMT*	Post-operative pain	[119]
rs6269	intronic	*COMT*	Post-operative pain	[119]
rs4633	exonic	*COMT*	Cancer Pain	[120]
rs4680	exonic	*COMT*	Cancer Pain	[120]
rs165656	intronic	*COMT*	Cancer Pain	[120]
rs165774	intronic	*COMT*	Cancer Pain	[120]

rs5993882	intronic	*COMT*	Cancer Pain	[120]
rs165599	UTR3	*COMT*	Cancer Pain	[120]
rs4680	exonic	*COMT*	Other Clinical Pain	[121]
rs4818	exonic	*COMT*	Post-operative pain	[122]
rs6269	intronic	*COMT*	Post-operative pain	[122]
rs4680	exonic	*COMT*	Post-operative pain	[123]
rs9332377	intronic	*COMT*	Temporomandibular disorders	[124]
rs4680	exonic	*COMT*	Analgesia	[125]
rs614230	downstream	*CX3CL1*	Post-operative pain	[126]
rs4680	exonic	*COMT*	Temporomandibular disorders	[127]
rs4379368	intronic	*SUGCT*	Migraine	[128]
rs2078371	intergenic	*TSPAN2;NGF*	Migraine	[129]
rs6693567	intergenic	*FALEC;ADAMTSL4*	Migraine	[129]
rs12134493	intergenic	*TSPAN2;NGF*	Migraine	[129]
rs4379368	intronic	*SUGCT*	Migraine	[129]
rs6478241	intronic	*ASTN2*	Migraine	[129]
rs186166891	intronic	*SUGCT*	Migraine	[129]
rs4814864	intronic	*SLC24A3*	Migraine	[129]
rs10786156	intronic	*PLCE1*	Migraine	[129]
rs6693567	intergenic	*FALEC;ADAMTSL4*	Migraine	[130]
rs1861881	intronic	*ASTN2*	Migraine	[130]
rs17171710	intronic	*SUGCT*	Migraine	[130]
rs4814864	intronic	*SLC24A3*	Migraine	[130]
rs6035355	intronic	*SLC24A3*	Migraine	[130]
rs6515020	intronic	*SLC24A3*	Migraine	[130]
rs57866767	intronic	*PLCE1*	Migraine	[130]

Supplementary data 3: Details for Mouse Pain Genetics Database for overlapped genes with genes identified in this review.

Gene	Number of Papers in the mouse database	Publication	Protein Name	Protein Acronym	Mouse Knockout Type	Tissue	Cellular Process	Function	Subfunction
ABCC4	1	[131]	ATP-binding cassette, sub-family C (CFTR/MRP), member 4	MRP4	Conventional	Whole Body	Metabolism	Transporter	Xenobiotic transport
COMT1	3	[132-134]	catechol-O-methyltransferase 1	COMT	Conventional	Whole Body	Metabolism	Neurotransmitter metabolism	G-protein coupled receptor
DTNBP1	1	[135]	dystrobrevin binding protein 1	DTNBP-1	Spontaneous	Whole Body	Cell structure	Intracellular trafficking	Synaptic vesicles
EFNB2	1	[136]	ephrin B2	ephB2	Cre/Lox	Nociceptor-specific	Cell signaling	Tyrosine kinase receptor	Neurotrophin
EGR1	1	[137]	early growth response 1	Zenk	Conventional	Whole Body	Gene regulation	Transcriptional regulation	Immediate-early gene
GFRA2	1	[138]	glial cell line derived neurotrophic factor family receptor alpha 2	GDNFa2	Conventional	Whole Body	Cell signaling	Tyrosine kinase receptor	Neurotrophin
HDAC4	1	[139]	histone deacetylase 4	HDAC4	Gene Trap	Whole Body	Cell signaling	Intracellular trafficking	
IL1	3	[140-142]	interleukin 1 complex	IL-1	Conventional	Whole Body	Cell signaling	Peptide signaling molecule	Cytokine
MAPK9	1	[143]	mitogen activated protein kinase 9	JNK2	Conventional	Whole Body	Cell signaling	Intracellular signal transduction	
NGF	1	[144]	nerve growth factor	NGFb	Conventional	Whole Body	Cell signaling	Peptide signaling molecule	Neurotrophin
OPRD1	8	[145-152]	opioid receptor, delta 1	DOR	Conventional	Whole Body	Cell signaling	G-protein coupled receptor	Opioid
PMP22	1	[153]	peripheral myelin protein 22	PMP22	Conventional	Whole Body	Cell structure	Myelination	
PPP1R9B	2	[154, 155]	protein phosphatase 1, regulatory subunit 9B		Conventional	Whole Body	Cell structure	Synaptic scaffolding	
PRKCA	1	[156]	protein kinase C, alpha	PKCa	Conventional	Whole Body	Cell signaling	Intracellular signal transduction	Protein kinase
TAC1	12	[157-168]	tachykinin 1	NK1	Conventional	Whole Body	Cell signaling	Peptide signaling molecule	Neuromodulator

Supplementary data legend

Supplementary data 1: full list of reported SNPs from all included papers. CHR: Chromosome. POS: Position, CHR_ band: Chromosome band, EA: Effect allele, EAF: Effect allele frequency, BETA (SE): Effect size (standard error).

Supplementary data 2: Publication list in the Human Pain Genetics Database (HPGDB) of overlapping genes between genes identified in this review and the HPGDB.

Supplementary data 3: Details for Mouse Pain Genetics Database for overlapped genes with genes identified in this review.

Reference

1. Rainero, I., et al., *A polymorphism in the interleukin-1alpha gene influences the clinical features of migraine.* Headache, 2002. **42**(5): p. 337-40.
2. Kim, H., et al., *Genetic influence on variability in human acute experimental pain sensitivity associated with gender, ethnicity and psychological temperament.* Pain, 2004. **109**(3): p. 488-496.
3. Diatchenko, L., et al., *Genetic basis for individual variations in pain perception and the development of a chronic pain condition.* Hum Mol Genet, 2005. **14**(1): p. 135-43.
4. Rakvåg, T.T., et al., *The Val158Met polymorphism of the human catechol-O-methyltransferase (COMT) gene may influence morphine requirements in cancer pain patients.* Pain, 2005. **116**(1-2): p. 73-8.
5. Diatchenko, L., et al., *Catechol-O-methyltransferase gene polymorphisms are associated with multiple pain-evoking stimuli.* Pain, 2006. **125**(3): p. 216-224.
6. Kim, H., et al., *Genetic predictors for acute experimental cold and heat pain sensitivity in humans.* J Med Genet, 2006. **43**(8): p. e40.
7. Reyes-Gibby, C.C., et al., *Exploring joint effects of genes and the clinical efficacy of morphine for cancer pain: OPRM1 and COMT gene.* Pain, 2007. **130**(1-2): p. 25-30.
8. Erdal, N., et al., *The A218C polymorphism of tryptophan hydroxylase gene and migraine.* J Clin Neurosci, 2007. **14**(3): p. 249-51.
9. Mishra, B.K., et al., *Do motor control genes contribute to interindividual variability in decreased movement in patients with pain?* Mol Pain, 2007. **3**: p. 20.
10. Vargas-Alarcón, G., et al., *Catechol-O-methyltransferase gene haplotypes in Mexican and Spanish patients with fibromyalgia.* Arthritis Res Ther, 2007. **9**(5): p. R110.
11. Ross, J.R., et al., *Genetic variation and response to morphine in cancer patients: catechol-O-methyltransferase and multidrug resistance-1 gene polymorphisms are associated with central side effects.* Cancer, 2008. **112**(6): p. 1390-403.
12. Nyholt, D.R., et al., *A high-density association screen of 155 ion transport genes for involvement with common migraine.* Hum Mol Genet, 2008. **17**(21): p. 3318-31.
13. Rakvåg, T.T., et al., *Genetic variation in the catechol-O-methyltransferase (COMT) gene and morphine requirements in cancer patients with pain.* Mol Pain, 2008. **4**: p. 64.
14. Jensen, K.B., et al., *Increased sensitivity to thermal pain following a single opiate dose is influenced by the COMT val(158)met polymorphism.* PLoS One, 2009. **4**(6): p. e6016.
15. Cohen, H., et al., *The relationship between a common catechol-O-methyltransferase (COMT) polymorphism val(158) met and fibromyalgia.* Clin Exp Rheumatol, 2009. **27**(5 Suppl 56): p. S51-6.
16. Yilmaz, I.A., et al., *Cytokine polymorphism in patients with migraine: some suggestive clues of migraine and inflammation.* Pain Med, 2010. **11**(4): p. 492-7.
17. Tchivileva, I.E., et al., *Effect of catechol-O-methyltransferase polymorphism on response to propranolol therapy in chronic musculoskeletal pain: a randomized, double-blind, placebo-controlled, crossover pilot study.* Pharmacogenet Genomics, 2010. **20**(4): p. 239-48.
18. Finan, P.H., et al., *Genetic influences on the dynamics of pain and affect in fibromyalgia.* Health Psychol, 2010. **29**(2): p. 134-42.
19. Mobascher, A., et al., *The val158met polymorphism of human catechol-O-methyltransferase (COMT) affects anterior cingulate cortex activation in response to painful laser stimulation.* Mol Pain, 2010. **6**: p. 32.
20. Fijal, B., et al., *The association of single nucleotide polymorphisms in the catechol-O-methyltransferase gene and pain scores in female patients with major depressive disorder.* J Pain, 2010. **11**(9): p. 910-5, 915.e1-9.
21. McLean, S.A., et al., *Catechol O-methyltransferase haplotype predicts immediate musculoskeletal neck pain and psychological symptoms after motor vehicle collision.* J Pain, 2011. **12**(1): p. 101-7.
22. Lee, P.J., et al., *Catecholamine-o-methyltransferase polymorphisms are associated with postoperative pain intensity.* Clin J Pain, 2011. **27**(2): p. 93-101.
23. Vossen, H., et al., *The genetic influence on the cortical processing of experimental pain and the moderating effect of pain status.* PLoS One, 2010. **5**(10): p. e13641.

24. Barbosa, F.R., et al., *Influence of catechol-O-methyltransferase (COMT) gene polymorphisms in pain sensibility of Brazilian fibromialgia patients.* Rheumatol Int, 2012. **32**(2): p. 427-30.
25. Matsuda, J.B., et al., *Serotonin receptor (5-HT 2A) and catechol-O-methyltransferase (COMT) gene polymorphisms: triggers of fibromyalgia?* Rev Bras Reumatol, 2010. **50**(2): p. 141-9.
26. Kolesnikov, Y., et al., *Combined catechol-O-methyltransferase and mu-opioid receptor gene polymorphisms affect morphine postoperative analgesia and central side effects.* Anesth Analg, 2011. **112**(2): p. 448-53.
27. Finan, P.H., et al., *COMT moderates the relation of daily maladaptive coping and pain in fibromyalgia.* Pain, 2011. **152**(2): p. 300-307.
28. Ligthart, L., et al., *Meta-analysis of genome-wide association for migraine in six population-based European cohorts.* Eur J Hum Genet, 2011. **19**(8): p. 901-7.
29. Laugsand, E.A., et al., *Clinical and genetic factors associated with nausea and vomiting in cancer patients receiving opioids.* Eur J Cancer, 2011. **47**(11): p. 1682-91.
30. Chasman, D.I., et al., *Genome-wide association study reveals three susceptibility loci for common migraine in the general population.* Nat Genet, 2011. **43**(7): p. 695-8.
31. Paz Aparicio, J., et al., *The IL-1β (+3953 T/C) gene polymorphism associates to symptomatic lumbar disc herniation.* Eur Spine J, 2011. **20 Suppl 3**(Suppl 3): p. 383-9.
32. Desmeules, J., et al., *Psychological distress in fibromyalgia patients: a role for catechol-O-methyl-transferase Val158met polymorphism.* Health Psychol, 2012. **31**(2): p. 242-249.
33. Fernández-de-las-Peñas, C., et al., *Catechol-O-methyltransferase genotype (Val158met) modulates cancer-related fatigue and pain sensitivity in breast cancer survivors.* Breast Cancer Res Treat, 2012. **133**(2): p. 405-12.
34. Smith, S.B., et al., *Potential genetic risk factors for chronic TMD: genetic associations from the OPPERA case control study.* J Pain, 2011. **12**(11 Suppl): p. T92-101.
35. Loggia, M.L., et al., *The catechol-O-methyltransferase (COMT) val158met polymorphism affects brain responses to repeated painful stimuli.* PLoS One, 2011. **6**(11): p. e27764.
36. Orrey, D.C., et al., *Catechol-O-methyltransferase genotype predicts pain severity in hospitalized burn patients.* J Burn Care Res, 2012. **33**(4): p. 518-23.
37. Schwahn, C., et al., *The effect of catechol-O-methyltransferase polymorphisms on pain is modified by depressive symptoms.* Eur J Pain, 2012. **16**(6): p. 878-89.
38. Jacobsen, L.M., et al., *The COMT rs4680 Met allele contributes to long-lasting low back pain, sciatica and disability after lumbar disc herniation.* Eur J Pain, 2012. **16**(7): p. 1064-9.
39. Sloan, J.A., et al., *Genetic variations and patient-reported quality of life among patients with lung cancer.* J Clin Oncol, 2012. **30**(14): p. 1699-704.
40. Martínez-Jauand, M., et al., *Pain sensitivity in fibromyalgia is associated with catechol-O-methyltransferase (COMT) gene.* Eur J Pain, 2013. **17**(1): p. 16-27.
41. Omair, A., et al., *Genetic contribution of catechol-O-methyltransferase variants in treatment outcome of low back pain: a prospective genetic association study.* BMC Musculoskelet Disord, 2012. **13**: p. 76.
42. Freilinger, T., et al., *Genome-wide association analysis identifies susceptibility loci for migraine without aura.* Nat Genet, 2012. **44**(7): p. 777-82.
43. Henker, R.A., et al., *The associations between OPRM 1 and COMT genotypes and postoperative pain, opioid use, and opioid-induced sedation.* Biol Res Nurs, 2013. **15**(3): p. 309-17.
44. Tammimäki, A. and P.T. Männistö, *Catechol-O-methyltransferase gene polymorphism and chronic human pain: a systematic review and meta-analysis.* Pharmacogenet Genomics, 2012. **22**(9): p. 673-91.
45. Fernández-de-Las-Peñas, C., et al., *Catechol-O-methyltransferase Val158Met polymorphism influences anxiety, depression, and disability, but not pressure pain sensitivity, in women with fibromyalgia syndrome.* J Pain, 2012. **13**(11): p. 1068-74.
46. Ahlers, S.J., et al., *The Val158Met polymorphism of the COMT gene is associated with increased pain sensitivity in morphine-treated patients undergoing a painful procedure after cardiac surgery.* Br J Clin Pharmacol, 2013. **75**(6): p. 1506-15.

47. Mamie, C., et al., *First evidence of a polygenic susceptibility to pain in a pediatric cohort.* Anesth Analg, 2013. **116**(1): p. 170-7.
48. Esserlind, A.L., et al., *Replication and meta-analysis of common variants identifies a genome-wide significant locus in migraine.* Eur J Neurol, 2013. **20**(5): p. 765-72.
49. Landau, R., et al., *The effect of OPRM1 and COMT genotypes on the analgesic response to intravenous fentanyl labor analgesia.* Anesth Analg, 2013. **116**(2): p. 386-91.
50. Michelotti, A., et al., *Catechol-O-methyltransferase (COMT) gene polymorphisms as risk factor in temporomandibular disorders patients from Southern Italy.* Clin J Pain, 2014. **30**(2): p. 129-33.
51. Omair, A., et al., *Treatment outcome of chronic low back pain and radiographic lumbar disc degeneration are associated with inflammatory and matrix degrading gene variants: a prospective genetic association study.* BMC Musculoskelet Disord, 2013. **14**: p. 105.
52. De Gregori, M., et al., *Genetic variability at COMT but not at OPRM1 and UGT2B7 loci modulates morphine analgesic response in acute postoperative pain.* Eur J Clin Pharmacol, 2013. **69**(9): p. 1651-8.
53. Belfer, I., et al., *Pain modality- and sex-specific effects of COMT genetic functional variants.* Pain, 2013. **154**(8): p. 1368-76.
54. Mu, J., et al., *Analysis of association between IL-1β, CASP-9, and GDF5 variants and low-back pain in Chinese male soldier: clinical article.* J Neurosurg Spine, 2013. **19**(2): p. 243-7.
55. Cargnin, S., et al., *An opposite-direction modulation of the COMT Val158Met polymorphism on the clinical response to intrathecal morphine and triptans.* J Pain, 2013. **14**(10): p. 1097-106.
56. Anttila, V., et al., *Genome-wide meta-analysis identifies new susceptibility loci for migraine.* Nat Genet, 2013. **45**(8): p. 912-917.
57. Reyes-Gibby, C.C., et al., *Symptom clusters of pain, depressed mood, and fatigue in lung cancer: assessing the role of cytokine genes.* Support Care Cancer, 2013. **21**(11): p. 3117-25.
58. Bortsov, A.V., L. Diatchenko, and S.A. McLean, *Complex multilocus effects of catechol-O-methyltransferase haplotypes predict pain and pain interference 6 weeks after motor vehicle collision.* Neuromolecular Med, 2014. **16**(1): p. 83-93.
59. Fernández-de-las-Peñas, C., et al., *Catechol-O-methyltransferase Val158Met polymorphism is associated with pain and disability, but not widespread pressure pain sensitivity, in women with carpal Tunnel syndrome.* Pain Physician, 2013. **16**(5): p. E591-600.
60. Rut, M., et al., *Influence of variation in the catechol-O-methyltransferase gene on the clinical outcome after lumbar spine surgery for one-level symptomatic disc disease: a report on 176 cases.* Acta Neurochir (Wien), 2014. **156**(2): p. 245-52.
61. Smith, S.B., et al., *Genetic variants associated with development of TMD and its intermediate phenotypes: the genetic architecture of TMD in the OPPERA prospective cohort study.* J Pain, 2013. **14**(12 Suppl): p. T91-101.e1-3.
62. Fernández-de-las-Peñas, C., et al., *Catechol-O-methyltransferase Val158Met polymorphism (rs4680) is associated with pain in multiple sclerosis.* J Pain, 2013. **14**(12): p. 1719-23.
63. Schistad, E.I., et al., *The interleukin-1α gene C>T polymorphism rs1800587 is associated with increased pain intensity and decreased pressure pain thresholds in patients with lumbar radicular pain.* Clin J Pain, 2014. **30**(10): p. 869-74.
64. Desmeules, J., et al., *Central pain sensitization, COMT Val158Met polymorphism, and emotional factors in fibromyalgia.* J Pain, 2014. **15**(2): p. 129-35.
65. Kambur, O., et al., *Effect of catechol-o-methyltransferase-gene (COMT) variants on experimental and acute postoperative pain in 1,000 women undergoing surgery for breast cancer.* Anesthesiology, 2013. **119**(6): p. 1422-33.
66. George, S.Z., et al., *Biopsychosocial influence on exercise-induced injury: genetic and psychological combinations are predictive of shoulder pain phenotypes.* J Pain, 2014. **15**(1): p. 68-80.
67. Sadhasivam, S., et al., *Genetics of pain perception, COMT and postoperative pain management in children.* Pharmacogenomics, 2014. **15**(3): p. 277-84.

68. George, S.Z., et al., *Inflammatory genes and psychological factors predict induced shoulder pain phenotype.* Med Sci Sports Exerc, 2014. **46**(10): p. 1871-81.
69. Loncar, Z., et al., *Do IL-1B and IL-1RN modulate chronic low back pain in patients with post-traumatic stress disorder?* Coll Antropol, 2013. **37**(4): p. 1237-44.
70. Oliveira, A., et al., *Interleukin-1β genotype and circulating levels in cancer patients: metastatic status and pain perception.* Clin Biochem, 2014. **47**(13-14): p. 1209-13.
71. Inanir, A., et al., *Clinical symptoms in fibromyalgia are associated to catechol-O-methyltransferase (COMT) gene Val158Met polymorphism.* Xenobiotica, 2014. **44**(10): p. 952-6.
72. Li, W., et al., *Pain in Parkinson's disease associated with COMT gene polymorphisms.* Behav Neurol, 2014. **2014**: p. 304203.
73. Chasman, D.I., et al., *Selectivity in genetic association with sub-classified migraine in women.* PLoS Genet, 2014. **10**(5): p. e1004366.
74. Gruber, H.E., et al., *A novel catechol-O-methyltransferase variant associated with human disc degeneration.* Int J Med Sci, 2014. **11**(7): p. 748-53.
75. Lee, Y.H., J.H. Kim, and G.G. Song, *Association between the COMT Val158Met polymorphism and fibromyalgia susceptibility and fibromyalgia impact questionnaire score: a meta-analysis.* Rheumatol Int, 2015. **35**(1): p. 159-66.
76. Jhun, E., et al., *Dopamine D3 receptor Ser9Gly and catechol-o-methyltransferase Val158Met polymorphisms and acute pain in sickle cell disease.* Anesth Analg, 2014. **119**(5): p. 1201-7.
77. Louter, M.A., et al., *Candidate-gene association study searching for genetic factors involved in migraine chronification.* Cephalalgia, 2015. **35**(6): p. 500-7.
78. Candiotti, K.A., et al., *Catechol-o-methyltransferase polymorphisms predict opioid consumption in postoperative pain.* Anesth Analg, 2014. **119**(5): p. 1194-200.
79. Moen, A., et al., *Role of IL1A rs1800587, IL1B rs1143627 and IL1RN rs2234677 genotype regarding development of chronic lumbar radicular pain; a prospective one-year study.* PLoS One, 2014. **9**(9): p. e107301.
80. Smith, S.B., et al., *Epistasis between polymorphisms in COMT, ESR1, and GCH1 influences COMT enzyme activity and pain.* Pain, 2014. **155**(11): p. 2390-9.
81. Sintas, C., et al., *Replication study of previous migraine genome-wide association study findings in a Spanish sample of migraine with aura.* Cephalalgia, 2015. **35**(9): p. 776-82.
82. Zhang, F., et al., *COMT gene haplotypes are closely associated with postoperative fentanyl dose in patients.* Anesth Analg, 2015. **120**(4): p. 933-40.
83. Belfer, I., et al., *Association of functional variations in COMT and GCH1 genes with postherniotomy pain and related impairment.* Pain, 2015. **156**(2): p. 273-279.
84. Yoshida, K., et al., *Prediction formulas for individual opioid analgesic requirements based on genetic polymorphism analyses.* PLoS One, 2015. **10**(1): p. e0116885.
85. Esserlind, A.L., et al., *The association between candidate migraine susceptibility loci and severe migraine phenotype in a clinical sample.* Cephalalgia, 2016. **36**(7): p. 615-23.
86. Xiao, J.L., et al., *Association of GDF5, SMAD3 and RUNX2 polymorphisms with temporomandibular joint osteoarthritis in female Han Chinese.* J Oral Rehabil, 2015. **42**(7): p. 529-36.
87. Omair, A., et al., *Catechol-O-methyltransferase (COMT) gene polymorphisms are associated with baseline disability but not long-term treatment outcome in patients with chronic low back pain.* Eur Spine J, 2015. **24**(11): p. 2425-31.
88. Tan, E.C., et al., *Common variants of catechol-O-methyltransferase influence patient-controlled analgesia usage and postoperative pain in patients undergoing total hysterectomy.* Pharmacogenomics J, 2016. **16**(2): p. 186-92.
89. Olesen, A.E., et al., *The genetic influences on oxycodone response characteristics in human experimental pain.* Fundam Clin Pharmacol, 2015. **29**(4): p. 417-25.
90. Slade, G.D., et al., *COMT Diplotype Amplifies Effect of Stress on Risk of Temporomandibular Pain.* J Dent Res, 2015. **94**(9): p. 1187-95.
91. Meloto, C.B., et al., *COMT gene locus: new functional variants.* Pain, 2015. **156**(10): p. 2072-2083.
92. Rodriguez-Acevedo, A.J., et al., *Common polygenic variation contributes to risk of migraine in the Norfolk Island population.* Hum Genet, 2015. **134**(10): p. 1079-87.
93. Lin, Q.F., et al., *Association of genetic loci for migraine susceptibility in the she people of China.* J Headache Pain, 2015. **16**: p. 553.

94. Yao, P., et al., *Effect of gene polymorphism of COMT and OPRM1 on the preoperative pain sensitivity in patients with cancer.* Int J Clin Exp Med, 2015. **8**(6): p. 10036-9.
95. Potapov, A.L. and A.V. Boiarkina, *[M1-OPIOID RECEPTOR AND CATECHOL-O-METILTRANSFERASE GENES POLYMORPHISM EFFECTS ON PERIOPERATIVE PSYCHOLOGICAL CONDITION OF THE PATIENTS AND THE EFFECTIVENESS OF POSTOPERATIVE ANALGESIA WITH OPIOIDS].* Anesteziol Reanimatol, 2015. **60**(3): p. 48-51.
96. Wang, X.S., et al., *Association of single nucleotide polymorphisms of ABCB1, OPRM1 and COMT with pain perception in cancer patients.* J Huazhong Univ Sci Technolog Med Sci, 2015. **35**(5): p. 752-758.
97. Bonato, L.L., et al., *ESRRB polymorphisms are associated with comorbidity of temporomandibular disorders and rotator cuff disease.* Int J Oral Maxillofac Surg, 2016. **45**(3): p. 323-31.
98. Chen, H., et al., *Association Between Polymorphisms of DRD2, COMT, DBH, and MAO-A Genes and Migraine Susceptibility: A Meta-Analysis.* Medicine (Baltimore), 2015. **94**(47): p. e2012.
99. Park, D.J., et al., *Association between catechol-O-methyl transferase gene polymorphisms and fibromyalgia in a Korean population: A case-control study.* Eur J Pain, 2016. **20**(7): p. 1131-9.
100. De Gregori, M., et al., *Human Genetic Variability Contributes to Postoperative Morphine Consumption.* J Pain, 2016. **17**(5): p. 628-36.
101. Gormley, P., et al., *Meta-analysis of 375,000 individuals identifies 38 susceptibility loci for migraine.* Nat Genet, 2016. **48**(8): p. 856-66.
102. Thomazeau, J., et al., *Predictive Factors of Chronic Post-Surgical Pain at 6 Months Following Knee Replacement: Influence of Postoperative Pain Trajectory and Genetics.* Pain Physician, 2016. **19**(5): p. E729-41.
103. Somogyi, A.A., et al., *Ethnicity-dependent influence of innate immune genetic markers on morphine PCA requirements and adverse effects in postoperative pain.* Pain, 2016. **157**(11): p. 2458-2466.
104. Mladenovic, I., et al., *Genetic Polymorphisms of Catechol-O-Methyltransferase: Association with Temporomandibular Disorders and Postoperative Pain.* J Oral Facial Pain Headache, 2016. **30**(4): p. 302-310.
105. An, X.K., et al., *Multilocus analysis reveals three candidate genes for Chinese migraine susceptibility.* Clin Genet, 2017. **92**(2): p. 143-149.
106. Perera, R.S., et al., *Single Nucleotide Variants of Candidate Genes in Aggrecan Metabolic Pathway Are Associated with Lumbar Disc Degeneration and Modic Changes.* PLoS One, 2017. **12**(1): p. e0169835.
107. Nielsen, L.M., et al., *Genetic Influences of OPRM1, OPRD1 and COMT on Morphine Analgesia in a Multi-Modal, Multi-Tissue Human Experimental Pain Model.* Basic Clin Pharmacol Toxicol, 2017. **121**(1): p. 6-12.
108. Young, E.E., et al., *Variations in COMT and NTRK2 Influence Symptom Burden in Women Undergoing Breast Cancer Treatment.* Biol Res Nurs, 2017. **19**(3): p. 318-328.
109. Matsuoka, H., et al., *Prospective replication study implicates the catechol-O-methyltransferase Val(158)Met polymorphism as a biomarker for the response to morphine in patients with cancer.* Biomed Rep, 2017. **7**(4): p. 380-384.
110. Quinelato, V., et al., *Association Between Polymorphisms in the Genes of Estrogen Receptors and the Presence of Temporomandibular Disorders and Chronic Arthralgia.* J Oral Maxillofac Surg, 2018. **76**(2): p. 314.e1-314.e9.
111. Mladenovic, I., et al., *Pulp Sensitivity: Influence of Sex, Psychosocial Variables, COMT Gene, and Chronic Facial Pain.* J Endod, 2018. **44**(5): p. 717-721.e1.
112. Zhang, Y., et al., *Association of genetic variation in COMT gene with pain related to sickle cell disease in patients from the walk-PHaSST study.* J Pain Res, 2018. **11**: p. 537-543.

113. Knisely, M.R., et al., *Associations Between Catecholaminergic and Serotonergic Genes and Persistent Breast Pain Phenotypes After Breast Cancer Surgery.* J Pain, 2018. **19**(10): p. 1130-1146.
114. Gutteridge, T., et al., *Single-Nucleotide Polymorphisms in TAOK3 Are Associated With High Opioid Requirement for Pain Management in Patients With Advanced Cancer Admitted to a Tertiary Palliative Care Unit.* J Pain Symptom Manage, 2018. **56**(4): p. 560-566.
115. Fernández-de-Las-Peñas, C., et al., *Catechol-O-Methyltransferase Val158Met Polymorphism Is Associated with Anxiety, Depression, and Widespread Pressure Pain Sensitivity in Women with Chronic, but Not Episodic, Migraine.* Pain Med, 2019. **20**(7): p. 1409-1417.
116. Lucenteforte, E., et al., *Opioid response in paediatric cancer patients and the Val158Met polymorphism of the human catechol-O-methyltransferase (COMT) gene: an Italian study on 87 cancer children and a systematic review.* BMC Cancer, 2019. **19**(1): p. 113.
117. Li, J., et al., *Candidate gene analyses for acute pain and morphine analgesia after pediatric day surgery: African American versus European Caucasian ancestry and dose prediction limits.* Pharmacogenomics J, 2019. **19**(6): p. 570-581.
118. Brancher, J.A., et al., *The association of genetic polymorphisms in serotonin transporter and catechol-O-methyltransferase on temporomandibular disorders and anxiety in adolescents.* J Oral Rehabil, 2019. **46**(7): p. 597-604.
119. Machoy-Mokrzyńska, A., et al., *Association of COMT gene variability with pain intensity in patients after total hip replacement.* Scand J Clin Lab Invest, 2019. **79**(3): p. 202-207.
120. Knisely, M.R., et al., *Associations Between Catecholaminergic and Serotonergic Genes and Persistent Arm Pain Severity Following Breast Cancer Surgery.* J Pain, 2019. **20**(9): p. 1100-1111.
121. Xu, J., et al., *Catechol-O-methyltransferase polymorphism Val158Met is associated with distal neuropathic pain in HIV-associated sensory neuropathy.* Aids, 2019. **33**(10): p. 1575-1582.
122. Hoofwijk, D.M.N., et al., *Genetic polymorphisms and prediction of chronic post-surgical pain after hysterectomy-a subgroup analysis of a multicenter cohort study.* Acta Anaesthesiol Scand, 2019. **63**(8): p. 1063-1073.
123. Margarit, C., et al., *Genetic Contribution in Low Back Pain: A Prospective Genetic Association Study.* Pain Pract, 2019. **19**(8): p. 836-847.
124. de Souza Tesch, R., et al., *Evaluation of genetic risk related to catechol-O-methyltransferase (COMT) and β2-adrenergic receptor (ADRB2) activity in different diagnostic subgroups of temporomandibular disorder in Brazilian patients.* Int J Oral Maxillofac Surg, 2020. **49**(2): p. 237-243.
125. Colloca, L., et al., *OPRM1 rs1799971, COMT rs4680, and FAAH rs324420 genes interact with placebo procedures to induce hypoalgesia.* Pain, 2019. **160**(8): p. 1824-1834.
126. Ma, G., et al., *Correlation between CCL2, CALCA, and CX3CL1 gene polymorphisms and chronic pain after cesarean section in Chinese Han women: A case control study.* Medicine (Baltimore), 2019. **98**(34): p. e16706.
127. Nascimento, T.D., et al., *μ-Opioid Activity in Chronic TMD Pain Is Associated with COMT Polymorphism.* J Dent Res, 2019. **98**(12): p. 1324-1331.
128. Kaur, S., et al., *Could rs4379368 be a genetic marker for North Indian migraine patients with aura?: Preliminary evidence by a replication study.* Neurosci Lett, 2019. **712**: p. 134482.
129. Zhao, Y., et al., *Genetic variants in migraine: a field synopsis and systematic re-analysis of meta-analyses.* J Headache Pain, 2020. **21**(1): p. 13.
130. Guo, Y., et al., *A genome-wide cross-phenotype meta-analysis of the association of blood pressure with migraine.* Nat Commun, 2020. **11**(1): p. 3368.
131. Lin, Z.P., et al., *Disruption of cAMP and prostaglandin E2 transport by multidrug resistance protein 4 deficiency alters cAMP-mediated signaling and nociceptive response.* Mol Pharmacol, 2008. **73**(1): p. 243-51.
132. Papaleo, F., et al., *Genetic dissection of the role of catechol-O-methyltransferase in cognition and stress reactivity in mice.* J Neurosci, 2008. **28**(35): p. 8709-23.

133. Kambur, O., et al., *Stress-induced analgesia and morphine responses are changed in catechol-O-methyltransferase-deficient male mice.* Basic Clin Pharmacol Toxicol, 2008. **103**(4): p. 367-73.
134. Walsh, J., et al., *Disruption of thermal nociceptive behaviour in mice mutant for the schizophrenia-associated genes NRG1, COMT and DISC1.* Brain Res, 2010. **1348**: p. 114-9.
135. Bhardwaj, S.K., et al., *Behavioral characterization of dysbindin-1 deficient sandy mice.* Behav Brain Res, 2009. **197**(2): p. 435-41.
136. Zhao, J., et al., *Nociceptor-expressed ephrin-B2 regulates inflammatory and neuropathic pain.* Mol Pain, 2010. **6**: p. 77.
137. Ko, S.W., et al., *Selective contribution of Egr1 (zif/268) to persistent inflammatory pain.* J Pain, 2005. **6**(1): p. 12-20.
138. Lindfors, P.H., et al., *Deficient nonpeptidergic epidermis innervation and reduced inflammatory pain in glial cell line-derived neurotrophic factor family receptor alpha2 knock-out mice.* J Neurosci, 2006. **26**(7): p. 1953-60.
139. Rajan, I., et al., *Loss of the putative catalytic domain of HDAC4 leads to reduced thermal nociception and seizures while allowing normal bone development.* PLoS One, 2009. **4**(8): p. e6612.
140. Honore, P., et al., *Interleukin-1alphabeta gene-deficient mice show reduced nociceptive sensitivity in models of inflammatory and neuropathic pain but not post-operative pain.* Behav Brain Res, 2006. **167**(2): p. 355-64.
141. Honore, P., et al., *The antihyperalgesic activity of a selective P2X7 receptor antagonist, A-839977, is lost in IL-1alphabeta knockout mice.* Behav Brain Res, 2009. **204**(1): p. 77-81.
142. Nadeau, S., et al., *Functional recovery after peripheral nerve injury is dependent on the pro-inflammatory cytokines IL-1β and TNF: implications for neuropathic pain.* J Neurosci, 2011. **31**(35): p. 12533-42.
143. Manassero, G., et al., *Role of JNK isoforms in the development of neuropathic pain following sciatic nerve transection in the mouse.* Mol Pain, 2012. **8**: p. 39.
144. Crowley, C., et al., *Mice lacking nerve growth factor display perinatal loss of sensory and sympathetic neurons yet develop basal forebrain cholinergic neurons.* Cell, 1994. **76**(6): p. 1001-11.
145. Zhu, Y., et al., *Retention of supraspinal delta-like analgesia and loss of morphine tolerance in delta opioid receptor knockout mice.* Neuron, 1999. **24**(1): p. 243-52.
146. Filliol, D., et al., *Mice deficient for delta- and mu-opioid receptors exhibit opposing alterations of emotional responses.* Nat Genet, 2000. **25**(2): p. 195-200.
147. Martin, M., et al., *Acute antinociceptive responses in single and combinatorial opioid receptor knockout mice: distinct mu, delta and kappa tones.* Eur J Neurosci, 2003. **17**(4): p. 701-8.
148. Scherrer, G., et al., *The delta agonists DPDPE and deltorphin II recruit predominantly mu receptors to produce thermal analgesia: a parallel study of mu, delta and combinatorial opioid receptor knockout mice.* Eur J Neurosci, 2004. **19**(8): p. 2239-48.
149. Nadal, X., et al., *Neuropathic pain is enhanced in delta-opioid receptor knockout mice.* Eur J Neurosci, 2006. **23**(3): p. 830-4.
150. Contet, C., et al., *Dissociation of analgesic and hormonal responses to forced swim stress using opioid receptor knockout mice.* Neuropsychopharmacology, 2006. **31**(8): p. 1733-44.
151. Benbouzid, M., et al., *Delta-opioid receptors are critical for tricyclic antidepressant treatment of neuropathic allodynia.* Biol Psychiatry, 2008. **63**(6): p. 633-6.
152. Gavériaux-Ruff, C., et al., *Inflammatory pain is enhanced in delta opioid receptor-knockout mice.* Eur J Neurosci, 2008. **27**(10): p. 2558-67.
153. Samsam, M., et al., *Impaired sensory function in heterozygous P0 knockout mice is associated with nodal changes in sensory nerves.* J Neurosci Res, 2002. **67**(2): p. 167-73.
154. Charlton, J.J., et al., *Multiple actions of spinophilin regulate mu opioid receptor function.* Neuron, 2008. **58**(2): p. 238-47.
155. Nag, S., et al., *Knockout of spinophilin, an endogenous antagonist of arrestin-dependent alpha2-adrenoceptor functions, enhances receptor-mediated antinociception yet does not eliminate sex-related differences.* Behav Brain Res, 2009. **197**(2): p. 457-61.

156. Zhao, C., M. Leitges, and R.W.t. Gereau, *Isozyme-specific effects of protein kinase C in pain modulation.* Anesthesiology, 2011. **115**(6): p. 1261-70.
157. Cao, Y.Q., et al., *Primary afferent tachykinins are required to experience moderate to intense pain.* Nature, 1998. **392**(6674): p. 390-4.
158. Zimmer, A., et al., *Hypoalgesia in mice with a targeted deletion of the tachykinin 1 gene.* Proc Natl Acad Sci U S A, 1998. **95**(5): p. 2630-5.
159. Inoue, M., et al., *Nociceptin/orphanin FQ-induced nociceptive responses through substance P release from peripheral nerve endings in mice.* Proc Natl Acad Sci U S A, 1998. **95**(18): p. 10949-53.
160. Mansikka, H., et al., *Nerve injury-induced mechanical but not thermal hyperalgesia is attenuated in neurokinin-1 receptor knockout mice.* Exp Neurol, 2000. **162**(2): p. 343-9.
161. Vergnolle, N., et al., *Proteinase-activated receptor-2 and hyperalgesia: A novel pain pathway.* Nat Med, 2001. **7**(7): p. 821-6.
162. Bilkei-Gorzo, A., et al., *Increased morphine analgesia and reduced side effects in mice lacking the tac1 gene.* Br J Pharmacol, 2010. **160**(6): p. 1443-52.
163. Dubois, D. and L. Gendron, *Delta opioid receptor-mediated analgesia is not altered in preprotachykinin A knockout mice.* Eur J Neurosci, 2010. **32**(11): p. 1921-9.
164. Lin, C.C., et al., *An antinociceptive role for substance P in acid-induced chronic muscle pain.* Proc Natl Acad Sci U S A, 2012. **109**(2): p. E76-83.
165. Sahbaie, P., et al., *Preprotachykinin-A gene disruption attenuates nociceptive sensitivity after opioid administration and incision by peripheral and spinal mechanisms in mice.* J Pain, 2012. **13**(10): p. 997-1007.
166. Botz, B., et al., *Role of Pituitary Adenylate-Cyclase Activating Polypeptide and Tac1 gene derived tachykinins in sensory, motor and vascular functions under normal and neuropathic conditions.* Peptides, 2013. **43**: p. 105-12.
167. Guo, T.Z., et al., *Neuropeptide deficient mice have attenuated nociceptive, vascular, and inflammatory changes in a tibia fracture model of complex regional pain syndrome.* Mol Pain, 2012. **8**: p. 85.
168. Borbély, E., et al., *Role of tachykinin 1 and 4 gene-derived neuropeptides and the neurokinin 1 receptor in adjuvant-induced chronic arthritis of the mouse.* PLoS One, 2013. **8**(4): p. e61684.
